# A mapping review of sacrococcygeal pilonidal sinus disease

**DOI:** 10.1007/s10151-021-02432-9

**Published:** 2021-03-16

**Authors:** M. Kumar, W. H. Clay, M. J. Lee, S. R. Brown, D. Hind

**Affiliations:** 1 The Medical School, University of Sheffield, Sheffield, UK; 2 Academic Directorate of General Surgery, Sheffield Teaching Hospitals NHS Foundation Trust, Sheffield, UK; 3 Department of Oncology and Metabolism, FU32, F Floor, Medical School, University of Sheffield, Sheffield, S10 2RX UK; 4 Clinical Trials Research Unit, School of Health and Related Research, University of Sheffield, Sheffield, UK

**Keywords:** Pilonidal sinus, Mapping review, Systematic map, Surgery, Wound care

## Abstract

**Background:**

Pilonidal sinus is a hole in the natal cleft which may cause severe pain and become infected. The evidence base for management of pilonidal sinus is said to be poor quality, poorly focused and rapidly proliferating. We undertook a systematic mapping review to provide a broad overview of the field and support the identification of research priorities.

**Methods:**

We searched MEDLINE, CINAHL, and EMBASE from inception to 22nd Nov 2020 for primary research studies focused on the management of pilonidal sinus. We extracted data on study design and categorised studies under five major headings (‘non-surgical treatment’, ‘surgical treatment’, ‘aftercare’ and ‘other’), producing frequency counts for different study designs. Gaps in research were identified from published systematic reviews and tabulated.

**Results:**

We identified 983 eligible studies, of which 36 were systematic reviews and/or meta-analyses; 121 were randomised controlled trials), and 826 observational studies of various design. The majority of studies evaluated surgical techniques (*n* = 665), or adjuvant medical interventions (*n* = 98). The literature on wound care has developed most recently, and the evidence base includes 30% randomised controlled trials. Gaps analysis highlighted comparison of surgical techniques including flaps, laser depilation, and wound care interventions as potential areas for randomised controlled trials.

**Conclusions:**

This mapping review summarises eight decades of research on the management of pilonidal sinus. Further research is needed to identify front-running interventions, understand variation in practice and patient values, and to prioritise future research.

**Supplementary Information:**

The online version contains supplementary material available at 10.1007/s10151-021-02432-9.

## Introduction

Pilonidal sinus disease predominantly refers to the condition of in-growing hair in the natal cleft. It typically affects younger, and economically active, men, and can cause pain and local sepsis [1]. It is often recurrent and challenging to treat [2]. Even where healing of the primary cause is achieved, there can be longer-term challenges related to wound healing [3]. The absence of a front-running intervention reliably associated with long-term healing, and concerns about research deficits [4] have led to widespread variation in practice [5] and concern that practice is not evidence based [6]. The rapidly proliferating scientific literature on pilonidal sinus is said to be poor and to comprise principally of single-centre case series and non-randomised comparative studies, leading to calls for more focused, better-quality research [4].

Mapping reviews, or systematic maps, use systematic searches on a broad topic, to make large bodies of literature “accessible, digestible and useable” [7]. Rather than addressing the findings of studies, they assess activity in an area of research. This allows researchers to identify which aspects of a problem have publications associated with them [8]. Where activity cannot be identified related to an aspect of a disease or condition, it might be considered a gap. Mapping reviews are an increasingly used method of identifying research gaps and priorities in a broad field [8, 9]. In surgery, mapping reviews have previously summarised the literature, and clinical uncertainty, in broad fields such as oral and maxillofacial surgery [10] and tele-orthopaedics [11]. In this paper, we report a mapping review of sacrococcygeal pilonidal sinus.

The aim of the study was to categorise the literature using accessible graphical formats to highlight the most robust current evidence. This will allow healthcare professionals to understand what areas of the disease are well or less well researched, improving decision-making and guiding future research.

## Materials and methods

### Search strategy

Searches of the MEDLINE, EMBASE, and CINAHL databases were performed, from inception of database to 22nd Nov 2020 for citations indexed with the MeSH term “pilonidal sinus”, without date or language restrictions.

### Study selection

Eligible citations were systematic reviews, controlled trials, cohort studies, case–control studies; cross-sectional studies; ecological studies; case series and case reports. Citations for which no abstract was available, case reports based on a single patient, non-systematic review articles, comments and letters were all ineligible for inclusion. Articles for which the primary focus was not sacrococcygeal pilonidal sinus were also excluded from this mapping review.

### Data extraction

From the study abstracts and full-text articles where necessary (i.e., where abstract was not available), we extracted the date of publication, type of study, intervention, comparator (if any), outcome measures, MeSH terms identified as the focus of the article, and the number of patients.

### Categorisation

The study group agreed on a clinical categorisation system which included four major headings (Table 1): non-surgical treatment, surgical treatment, aftercare, and other. ‘Non-surgical treatment’ was subcategorised into hair removal and other. ‘Surgery’ was subcategorised into surgical techniques and chemicals or drugs. ‘Surgical techniques’ was further broken down with the assistance of two clinicians into flaps; off-midline closure; midline closure; excision only; minimal excision only; marsupialisation; drainage; endoscopic; laser; radiofrequency and other. ‘Chemicals and drugs’ was further broken down into phenol; fibrin; methylene blue; platelet-rich plasma; pre/intraoperative antibiotics and other. ‘Aftercare’ was subcategorised into wound care and non-wound care. The subcategory of ‘wound care’ was further broken down into negative pressure wound therapy; foam dressing; alginate dressing; hydrocolloid dressing; hydrofibre dressing; hydrogel dressing; gauze; platelet-rich plasma; postoperative antibiotics and other.

**Table 1:** Taxonomy of studies

Major heading	Minor heading	Sub-group
Non-surgical treatment	Hair removal	–
	Other	–
Surgical treatment	Surgical techniques	Flaps
		Off midline closure
		Midline closure
		Excision only
		Minimal excision
		Marsupialisation
		drainage
		endoscopic treatment
		Laser treatment
		radiofrequency treatment
		Other
	Chemicals and drugs	Phenol
		Fibrin
		Methylene blue
		Platelet rich plasma
		Pre/intraoperative antibiotics
		Other
Aftercare	Wound-care	Negative pressure wound therapy
		Foam dressing
		Alginate dressing
		Hydrocolloid dressing
		Hydrofibre dressing
		Hydrogel dressing
		Gauze
		Platelet rich plasma
		Post-operative antibiotics
	Other	–
Other	Aetiology and complications	Patient risk factors
		Post-operative complications
		Family history
		Hair analysis
		Microbiology
		Cellular/chemical factors
		Coexisting conditions
		Anatomical factors
	Epidemiology	–
	Qualitative	–
	Health utility	–
	Other	–

The category of ‘other’ was further subcategorised into aetiology and complications; epidemiology; health utility and other. The subcategory aetiology and complications was further broken down into patient risk factors; postoperative complications; family history; hair analysis; microbiology; cellular/chemical factors; coexisting conditions, and anatomical factors. Since one study may investigate more than one intervention, some of the studies overlap between the categories and subcategories. After the studies had been categorised and subcategorised, a master table was created, with frequency counts (numbers of studies) for clinical categories, and subcategories, as well as for each type of study design.

### Gaps analysis

We accessed the full texts of systematic reviews identified through the searches, and tabulated explicit recommendations, for further research. In areas where there were not yet systematic reviews, we noted whether there was more than one, one, or no randomised controlled trials (RCTs) yet.

## Results

Searches identified 3202 citations indexed with the MeSH heading ‘pilonidal sinus’ and in Embase, CINAHL, and Central. At screening, 2156 were excluded. Of the remaining 983 studies, published between 1945 and 2020 36 were systematic reviews and/or meta-analyses; 121 were RCTs, and 826 observational studies of various design.

### Categorisation

**Fig. 1:**
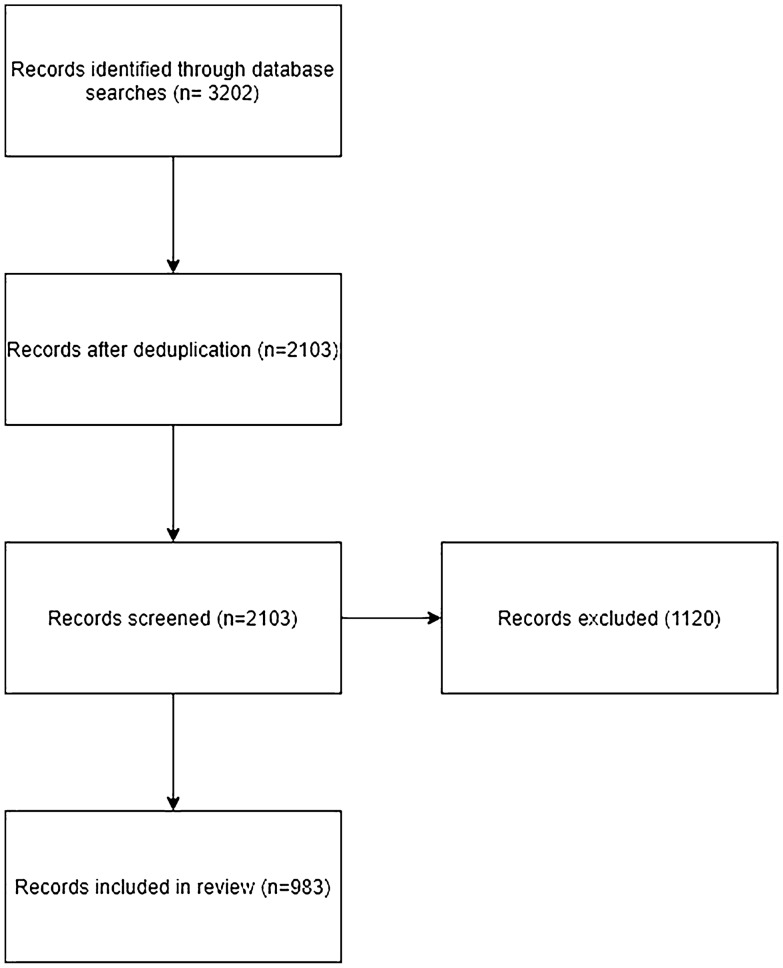
PRISMA flow chart

**Fig. 2:**
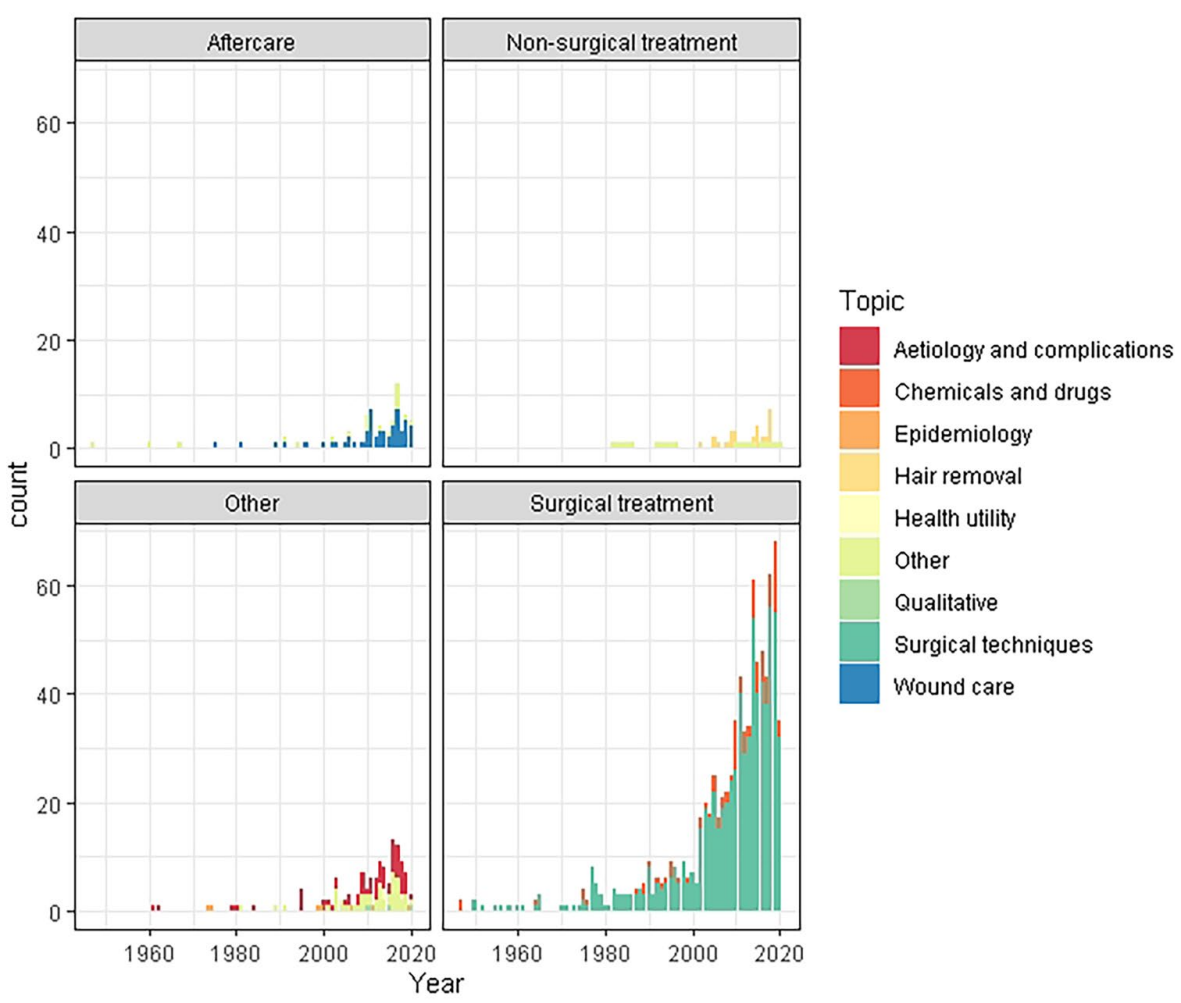
Major and minor domains identified by year published

**Fig. 3:**
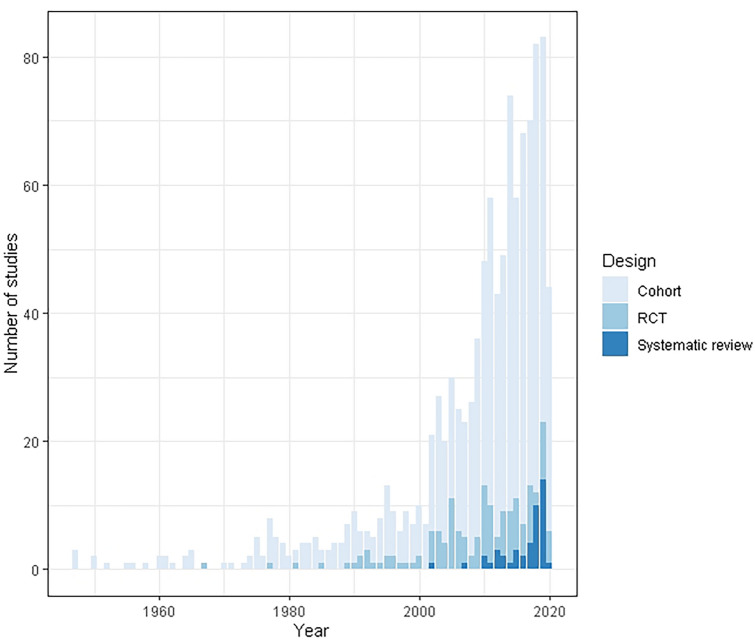
Study design used by year published

The number of studies included in each category, and the relevant study design are presented in Fig. 1 and in the web-only Appendix. Figure 2 demonstrates the coverage of domains by year of publication, and Fig. 3 demonstrates the study designs reported each year. These demonstrate that the majority of the literature addresses surgical techniques, and is dominated by cohort studies.

### Non-surgical treatment

In the category of non-surgical *treatment,* there were 4 systematic reviews, 2 RCTs and 29 observational studies of various design. Topics included: hair removal (4 systematic reviews; 1 RCT and 28 observational studies); conservative treatment (1 RCT and 3 observational studies).

### Surgery

In the category of surgery, there were 26 systematic reviews/meta-analyses, 80 RCTs and 622 observational studies of various designs.

Under the subheading surgical techniques, the topics included: flap (14 systematic reviews/meta-analyses; 38 RCTs and 222 observational studies); midline closure (10 systematic reviews/meta-analyses; 30 RCTs and 163 observational studies); off-midline closure (12 systematic reviews/meta-analyses; 21 RCTs and 116 observational studies); excision only (9 systematic reviews/meta-analyses; 28 RCTs and 121 observational studies); minimal excision only (6 systematic reviews/meta-analyses; 3 RCTs and 61 observational studies); marsupialisation (2 systematic reviews/meta-analyses; 6 RCTs and 31 observational studies); endoscopic (4 systematic reviews/meta-analyses; 1 RCTs and 47 observational studies); drainage (1 systematic reviews/meta-analyses and 8 RCTs); laser (2 systematic reviews/meta-analyses and 23 observational studies); radiofrequency (1 systematic reviews/meta-analyses; 3 RCTs and 3 observational studies); other (2 systematic review/meta-analyses; 4 RCTs and 32 observational studies) (Fig. 2).

Under the subheading chemical and drugs, the topics included: phenol (4 systematic reviews/meta-analyses; 3 RCTs and 31 observational studies); fibrin glue (4 systematic reviews/meta-analyses; 3 RCTs and 11 observational studies); pre/intraoperative antibiotics (2 systematic reviews/meta-analyses; 16 RCTs and 5 observational studies); methylene blue (7 observational studies); platelet-rich plasma (2 RCTs and 2 observational study); other (1 RCT and 6 observational studies) (Fig. 3).

### Aftercare

In the category of aftercare, there were: 3 systematic reviews; 20 RCTs and 51 observational studies of various designs.

Under the subheading of wound care topics included: negative pressure wound therapy (1 systematic review; 2 RCTs and 15 observational studies); foam (1 systematic review; 2 RCTs and 3 observational studies); alginate (1 systematic review; 3 RCTs and 2 observational studies); hydrocolloid (2 RCTs and 2 observational studies); hydrogel (1 RCT and 1 observational study); hydrofibre (1 observational study); gauze (2 RCTs and 6 observational study); collagenase dressing (1 RCT and 1 observational study); silver dressing (2 observational studies); hydrophilic dressing (1 observational study); platelet-rich plasma therapy (1 systematic review; 1 RCT and 2 observational studies); postoperative antibiotics (1 systematic review; 3 RCTs and 8 observational studies); other (1 systematic review; 9 RCT and 17 observational studies). Under the subheading non-wound care*,* there were 2 RCTs.

### Other

In the category of other*,* there was: 1 systematic review; 10 RCTs and 119 observational studies of various designs.

The subgroup aetiology and complications included: patient risk factors (*n* = 28); risk factors for postoperative complications (*n* = 17); family history (*n* = 3); hair analysis (*n* = 5); microbiology (*n* = 4); cellular/chemical factors (*n* = 11); coexisting conditions (*n* = 5); anatomical factors (*n* = 6). The epidemiology descriptor was assigned to 8 studies, and quality of life descriptor was used for four studies. All studies in this group were cohort studies. There were four qualitative research studies.

### Gaps analysis

Most systematic reviews recommended RCTs of non-specific design with longer follow-up and improved methods, especially in approaches to aftercare (Table 2). For surgical techniques, there is a need for trials comparing different types of flaps [12, 13], and comparing midline vs off-centre closure [14, 15]. There is also a need for trials comparing minimally invasive procedures with standard care [16–18]. Reviews noted a lack of trials addressing emergency treatment of pilonidal sinus, the role of lateral excision only, cryosurgery, or use of setons. There were gaps in data addressing the preservation or obliteration of natal cleft, and in the use of photodynamic therapy. In the use of chemicals and drugs, there is a need for the economic evaluation of gentamicin collagen sponges and implants [19]. Systematic reviews also called for RCTs addressing the use of methylene blue. There were also gaps in data exploring laser depilation to prevent recurrence [20, 21]. There was a clear need for further data exploring the roles of wound care adjuncts in the aftercare setting [22, 23].

**Table 2:** Gaps analysis

Field	Recommendation
Surgery	Systematic reviews call for: trials comparing Limberg versus Karydakis flaps [12, 13]; trials of primary tension-free midline closure in the absence of lateral pits [14]; trials of flap with off-midline repairs [15]; trials evaluating off-midline closure [46]; trials of minimally invasive techniques [16]; trials of standardised endoscopic techniques versus other minimally invasive or conventional procedures [17, 18]; prospective studies on—particularly minimally invasive—techniques in paediatrics [47, 48]; economic studies comparing flap repair and laying open [49]; and need for core outcome sets [47, 50] Topic with more than one RCT possibly meriting systematic review: diathermy Topic with insufficient RCTs to warrant systematic reviews: Bascoms 1 Topics with no RCTS: grafts; emergency care; lateral excision only; cryosurgery; setons, preservation or obliteration of natal cleft; photodynamic therapy
Chemicals and drugs	Systematic review calls for: economic studies on gentamicin collagen sponges and implants [19] Topics with no RCTs: methylene blue
Non-surgical	Systematic reviews call for: trials on laser hair depilation to prevent recurrence [20, 21] Topic with insufficient RCTs to warrant systematic reviews: conservative treatment
Aftercare	Systematic reviews call for: trials of platelet-rich plasma therapy [22]; further trials on gentamicin collagen sponges [23] Topics with more than one RCT, meriting systematic review: hydrocolloid; gauze Topics with insufficient RCTs to warrant systematic reviews: hydrogel Topics with no RCTs: hydrofibre, silver dressing; hydrophilic dressing

*RCT* randomised controlled trial

## Discussion

We undertook a mapping review of the published evidence on the management of pilonidal sinus. The published literature covered in this review spans eight decades. The findings show that, for most topics on which there are two or more randomised controlled trials, there is a relatively up-to-date systematic review, the exceptions being in the study of the effects of platelet-rich plasma, hydrocolloid dressings, gauze and postoperative antibiotics (Appendix).

The literature currently focuses on the management of pilonidal sinus using surgical treatments and adjuvant therapy in the form of supplementary chemicals and drugs. Only 12% of the 983 identified primary research articles were randomised trials. This suggests a reliance on cohort studies which, while appropriate for studies of natural history, risk factors, and early evaluation of novel approaches, cannot provide us with trustworthy estimates of treatment effects. In this regard, the wound care literature is more robust than other areas, with RCTs accounting for 25/83 (30.1%) of all primary research in this study.

Our aim was to provide a broad overview of the published literature rather than a more fine-grained analysis of a more specific and focused body of work, but we acknowledge several other limitations besides. The justification for drawing the gaps analysis on the recommendations of systematic reviews is that Cochrane reviews tend to concentrate on necessary methodological improvements, rather than providing explicit recommendations for further RCTs which may in fact be merited. On the other hand, readers should check trial registries for ongoing trials before acting on research recommendations from non-Cochrane reviews, which sometimes make the case for research the authors intend to undertake. While the involvement of a second reviewer is de rigueur in systematic reviews of therapeutic effectiveness [24], it is considerably more costly and only marginally more effective [25]. For this reason, we consider it defensible in an exercise intended to characterise a broad field, rather than inform policy on specific decision problems.

This mapping review confirms the absence of clear, front-running surgical interventions for pilonidal sinus [4]. In addition, the literature likely has problems relating to heterogeneity in the definition [26–31] and measurement [28, 32] of clinical outcomes. For this reason, calls to standardise endpoints within specialty areas are becoming increasingly common [30, 33, 34]. In addition to definitional harmonisation, core outcome sets can also help to reduce the selective reporting of outcomes (outcome reporting bias [35]).

A mapping review is not without its limitations. Notably, it takes a high-level approach in the assessment of the literature. This means the underlying studies have not been robustly quality assessed. As such, the recognition of ‘gaps’ in the literature relates to tallies of coverage rather than quantity and quality of studies. The categories selected were generated by the research team, and could be considered broad. The study classification system and allocations were reviewed by surgeons and researchers in the field, which should support their validity. The authors also noted the use of ‘standard care’ as a comparator. This may have changed over the decades but was not extracted here, so should be interpreted with caution. However, the broad search terms across multiple databases, and extensive numbers of citations reviewed can reassure readers that this is a fairly exhaustive list of studies in the field.

The publication of the Idea, Development, Exploration, Assessment, Long-term Follow-up (IDEAL) Framework [36] for improving the quality of surgical research has stimulated the development of large, multicentre, prospective longitudinal cohorts designed to understand variations in practice and their effects on outcomes [37–39]. The ongoing Pilonidal sinus Treatment—Studying the Options (PiTStOp) study aims to recruit 800 people with the aim of identifying the most common combinations of excision and closure techniques used in UK practice (ISRCTN95551898) [40]. While PiTStOP will report healing, recurrence and re-intervention rates, stratified by severity of disease, a principal objective is to inform a large nominal group technique consensus exercise, currently scheduled for Jul 2021, on optimal management and research priorities. Published qualitative research already describes the lived experience of pilonidal sinus [41, 42], the frequent disconnect between provision and the expectations of service-users [43], as well as the use of poorly evidenced interventions [6]. The PiTStOp programme of research will improve our understanding of the context of pilonidal sinus management through: large-scale surveys about shared decision-making [44] and decision regret [45]; a discrete choice experiment to assess which interventions patients would rather avoid and which outcomes they most value; semi-structured interviews aimed at understanding service-user decision-making and practical coping strategies.

This study provides a useful starting point for researchers. It has collated, in one place, a summary of the knowledge of the management of pilonidal sinus disease. It demonstrates the range and nature of interventions explored, albeit at a high level, and the types of studies underpinning these. These data, plus the gaps analysis, should provide researchers with a springboard for further research in the area.

## Conclusions

This systematic mapping review provides an accessible overview of eight decades of research on the management of pilonidal sinus. It confirms the presence of a range of interventions and adjuncts for the care of pilonidal sinus, many of which have not been explored in RCTs. Further research is needed to understand variation in practice and patient values, as well as to prioritise future research.

## Electronic supplementary material

Below is the link to the electronic supplementary material.

Supplementary file 1 (DOCX 1052kb)
