# Supplementary material for: A mapping review of sacrococcygeal pilonidal sinus disease
Source: Tech Coloproctol. 2021 Mar 16;25(6):675–82. doi: 10.1007/s10151-021-02432-9 (PMC8124060; doi:10.1007/s10151-021-02432-9)
Supplement: Supplementary file 1 — (DOCX 1052kb) [file 10151_2021_2432_MOESM1_ESM.docx]

Surgery

| Surgical Technique | Evidence | Citations |
| --- | --- | --- |
| Flaps | 10 systematic reviews | (1–10) |
|  | 4 meta-analyses | (11–14) |
|  | 38 RCTs | (15–52) |
|  | 222 observational studies | (53–274) |
| Off Midline closure | 9 systematic reviews | (1,3–8,10,275) |
|  | 3 meta-analyses | (11,13,276) |
|  | 21 RCTs | (17,18,21,24,27,30,43–45,52,277–287) |
|  | 116 observational studies | (55,116,127,137,139,143,149,150,152,153,159,163,166,179,208,210,223,224,226,242,246,250,253,288–380) |
| Midline closure | 5 systematic reviews | (6,7,275,381,382) |
|  | 5 meta-analyses | (11–14,276) |
|  | 30 RCTs | (15,19,22,23,26,34,35,37,42,47,49,278,383–400) |
|  | 163 observational studies | (60,92,100,106,109,110,112,124,129,131,141,149,152,154,155,161,166,174,178,189,191,193,195,197,201,210,214,215,217,219,221–223,228,242,245,253,254,274,289,292,293,296,307,308,318,321,323,368,373,375,401–512) |
| Excision only | 7 systematic reviews | (2,6,7,275,381,382,513) |
|  | 2 meta-analysis | (11,276) |
|  | 28 RCTs | (29,32,38,41,46,48,279,384–386,388,389,392,393,395–400,514–521) |
|  | 121 observational studies | (60,92,99,106,109,125,127,137,149,155–157,161,178,189,192,199,201,214,224,228,294,306,311,313,317,326,358,362,366,367,375,380,391,415,418,419,421,424,427,431,437,440,445,448,449,452,465,468,471–476,478,480–482,484,485,487,491,495,497,499,519,522–574) |
| Minimal excision only | 3 systematic reviews | (6,7,275) |
|  | 3 meta-analysis | (11,276,575) |
|  | 3 RCTs | (286,394,576) |
|  | 61 observational studies | (106,152,180,183,320,425,446,451,468,470,472,482,498,524,538,541,542,547,550,555,568,577–616) |
| Marsupialisation | 2 systematic reviews | (7,275) |
|  | 6 RCTs | (28,40,520,576,617,618) |
|  | 31 observational studies | (106,154,159,193,197,242,289,375,413,418,425,427,437,445,471,477,480,484,544,545,552,560,619–627) |
| Drainage | 1 systematic review | (628) |
|  | 8 RCTs | (20,25,39,281,283,387,629,630) |
| Endoscopic | 4 systematic reviews | (6,631–633) |
|  | 1 RCT | (634) |
|  | 47 observational studies | (181,366,426,486,578,579,635–674) |
| Laser | 2 systematic reviews | (6,7) |
|  | 23 observational studies | (364,377,541,597,612,675–692) |
| Radiofrequency ablation | 1 systematic review | (6) |
|  | 3 RCTs | (517,617,618) |
|  | 3 observational studies | (626,693,694) |
| Graft | 4 observational studies | (527,695–697) |
| Bascoms 1 | 1 RCT | (282) |
|  | 7 observational studies | (454,698–703) |
| Emergency | 3 observational studies | (704–706) |
| Electrocautery | 1 RCT | (516) |
|  | 1 observational study | (546) |
| Lateral excision only | 1 observational study | (559) |
| Cryosurgery | 2 observational studies | (707,708) |
| Seton | 1 observational study | (709) |
| Preserve natal cleft | 1 observational study | (710) |
| Obliterate natal cleft | 1 observational study | (710) |
| Vacuum assisted closure | 1 systematic review | (275) |
|  | 4 observational study | (290,424,445,711) |
| Scalpel | 2 RCT | (51,712) |
|  | 1 observational study | (531) |
| Diathermy | 2 RCT | (51,712) |
|  | 1 observational study | (531) |
| Multiple closure technique | 1 observational study | (713) |
| Antibiotic sutures | 1 observational study | (714) |
| Photodynamic therapy | 1 observational study | (715) |
| Complications | 1 observational study | (716) |
| All surgical techniques | 1 systematic review | (717) |
|  | 2 observational studies | (718,719) |

| Chemicals and Drugs | Evidence | Citations |
| --- | --- | --- |
| Phenol | 4 systematic reviews | (6,7,275,717) |
|  | 3 RCTs | (514,515,521) |
|  | 31 observational studies | (88,120,183,359,447,603,655,673,720–741) |
| Fibrin | 4 systematic reviews | (6,275,742,743) |
|  | 3 RCTs | (31,281,744) |
|  | 11 observational studies | (620,701,745–753) |
| Pre/intraoperative antibiotics | 2 systematic review | (754,755) |
|  | 16 RCTs | (386,393,756–769) |
|  | 5 observational studies | (538,770–773) |
| Methylene blue | 7 observational studies | (564,606,774–778) |
| Platelet rich plasma | 2 RCTs | (744,779) |
|  | 2 observational study | (465,682) |
| Other | 1 RCT | (779) |
|  | 6 observational studies | (441,527,772,780–782) |

Non-surgical treatment

| Category | Evidence | Citations |
| --- | --- | --- |
| Hair removal | 4 systematic reviews | (6,783–785) |
|  | 1 RCT | (786) |
|  | 28 observational studies | (270,377,487,541,721,787–809) |
| Conservative treatment | 1 RCT | (519) |
|  | 3 observational studies | (809–811) |

Aftercare

| Category | Type | Evidence | Citations |
| --- | --- | --- | --- |
| Woundcare | Negative pressure | 1 systematic review | (812) |
|  |  | 2 RCTs | (813,814) |
|  |  | 15 observational studies | (290,424,445,532,815–825) |
|  | Foam | 1 systematic review | (812) |
|  |  | 2 RCTs | (826,827) |
|  |  | 3 observational studies | (828–830) |
|  | Alginate | 1 systematic review | (812) |
|  |  | 3 RCTs | (826,831,832) |
|  |  | 2 observational studies | (820,833) |
|  | Hydrocolloid | 2 RCTs | (831,834) |
|  |  | 2 observational studies | (833,835) |
|  | Hydrogel | 1 RCT | (831) |
|  |  | 1 observational study | (836) |
|  | Hydrofibre | 1 observational study | (837) |
|  | Gauze | 2 RCT | (831,834) |
|  |  | 6 observational studies | (815,820,824,836–838) |
|  | Collagenase | 1 RCT | (839) |
|  |  | 1 observational study | (838) |
|  | Silver dressing | 2 observational studies | (668,830) |
|  | Hydrophilic dressing | 1 observational study | (840) |
|  | Platelet Rich plasma | 1 systematic review | (841) |
|  |  | 1 RCTs | (842) |
|  |  | 2 observational studies | (561,843) |
|  | Postoperative antibiotics | 1 systematic review | (844) |
|  |  | 3 RCTs | (765,767,769) |
|  |  | 8 observational studies | (460,845–851) |
|  | Other | 1 systematic review | (841) |
|  |  | 9 RCT | (814,827,832,842,852–856) |
|  |  | 17 observational studies | (448,558,668,772,857–869) |
| Non-woundcare |  | 2 RCTs | (870,871) |

Other

| Category | Type | Evidence | Citations |
| --- | --- | --- | --- |
| Aetiology and Complications | Patient risk factors for disease | 28 observational studies | (208,236,314,319,493,524,872–893) |
|  | Risk factors for postoperative complications | 17 observational studies | (208,236,304,314,319,371,375,376,453,493,534,540,878,891,894–896) |
|  | Family history | 3 observational studies | (877,882,892) |
|  | Hair analysis | 5 observational studies | (897–901) |
|  | Microbiology | 4 observational studies | (902–905) |
|  | Cellular/chemical level | 11 observational studies | (872,883,906–914) |
|  | Coexisting conditions | 5 observational studies | (873,915–918) |
|  | Anatomical risk factors | 5 observational studies | (919–923) |
| Epidemiology |  | 8 observational studies | (880,924–930) |
| QOL |  | 4 observational studies | (110,470,931,932) |
| Qualitative research |  | 4 observational studies | (933–936) |
| Other | Process | 2 RCT | (937,938) |
|  |  | 8 observational studies | (939–946) |
|  | Classification | 1 systematic review | (947) |
|  |  | 1 observational study | (948) |
|  | Surgeon grade | 3 observational studies | (250,949,950) |
|  | Investigation | 12 observational studies | (50,951–961) |
|  | Anaesthetics and pre-op | 7 RCTs | (962–968) |
|  |  | 9 observational studies | (969–977) |
|  | Cancer | 4 observational studies | (978–981) |
|  | Cost | 2 observational study | (109,982) |

1. Sahebally SM, McMahon G, Walsh SR, Burke JP. Classical Limberg versus classical Karydakis flaps for pilonidal disease- an updated systematic review and meta-analysis of randomized controlled trials. Surgeon [Internet]. 2019;17(5):300–8. Available from: http://ovidsp.ovid.com/ovidweb.cgi?T=JS&PAGE=reference&D=medl&NEWS=N&AN=30145045

2. Berthier C, Berard E, Meresse T, Grolleau J-L, Herlin C, Chaput B. A comparison of flap reconstruction vs the laying open technique or excision and direct suture for pilonidal sinus disease: A meta-analysis of randomised studies. Int Wound J [Internet]. 2019;16(5):1119–35. Available from: http://ovidsp.ovid.com/ovidweb.cgi?T=JS&PAGE=reference&D=medl&NEWS=N&AN=31230414

3. Gavriilidis P, Bota E. Limberg flap versus Karydakis flap for treating pilonidal sinus disease: a systematic review and meta-analysis. Can J Surg [Internet]. 2019;62(2):131–8. Available from: http://ovidsp.ovid.com/ovidweb.cgi?T=JS&PAGE=reference&D=medl&NEWS=N&AN=30697992

4. Prassas D, Rolfs T-M, Schumacher F-J, Krieg A. Karydakis flap reconstruction versus Limberg flap transposition for pilonidal sinus disease: a meta-analysis of randomized controlled trials. Langenbeck’s Arch Surg [Internet]. 2018;403(5):547–54. Available from: http://ovidsp.ovid.com/ovidweb.cgi?T=JS&PAGE=reference&D=med15&NEWS=N&AN=30066108

5. Boshnaq M, Phan YC, Martini I, Harilingam M, Akhtar M, Tsavellas G. Limberg flap in management of pilonidal sinus disease: systematic review and a local experience. Acta Chir Belg [Internet]. 2018;118(2):78–84. Available from: http://ovidsp.ovid.com/ovidweb.cgi?T=JS&PAGE=reference&D=med15&NEWS=N&AN=29390948

6. Grabowski J, Oyetunji TA, Goldin AB, Baird R, Gosain A, Lal DR, et al. The management of pilonidal disease: A systematic review. J Pediatr Surg [Internet]. 2019;54(11):2210–21. Available from: http://ovidsp.ovid.com/ovidweb.cgi?T=JS&PAGE=reference&D=medl&NEWS=N&AN=30948198

7. Stauffer VK, Luedi MM, Kauf P, Schmid M, Diekmann M, Wieferich K, et al. Common surgical procedures in pilonidal sinus disease: A meta-analysis, merged data analysis, and comprehensive study on recurrence. Sci Rep [Internet]. 2018;8(1):3058. Available from: http://ovidsp.ovid.com/ovidweb.cgi?T=JS&PAGE=reference&D=med15&NEWS=N&AN=29449548

8. M. A, S. H, W. M, P. S. Limberg flap for the management of pilonidal sinus reduces disease recurrence compared to Karydakis/Bascom procedure: A systematic review and meta-analysis of randomized controlled trials. Br J Surg [Internet]. 2019;106(Supplement 5):110. Available from: http://ovidsp.ovid.com/ovidweb.cgi?T=JS&PAGE=reference&D=emexb&NEWS=N&AN=631722080

9. Cai LZ, Chang J, Weiser TG, Forrester JD. Surgical Site Infections after Tissue Flaps Performed in Low- and Middle-Human Development Index Countries: A Systematic Review. Surg Infect (Larchmt) [Internet]. 2017 Oct;18(7):765–73. Available from: http://www.liebertonline.com/sur

10. Bi S, Sun K, Chen S, Gu J. Surgical procedures in the pilonidal sinus disease: a systematic review and network meta-analysis. Sci Rep [Internet]. 2020 Dec 13;10(1):13720. Available from: http://ovidsp.ovid.com/ovidweb.cgi?T=JS&PAGE=reference&D=emexb&NEWS=N&AN=632611496

11. Enriquez-Navascues JM, Emparanza JI, Alkorta M, Placer C. Meta-analysis of randomized controlled trials comparing different techniques with primary closure for chronic pilonidal sinus. Tech Coloproctol [Internet]. 2014;18(10):863–72. Available from: http://ovidsp.ovid.com/ovidweb.cgi?T=JS&PAGE=reference&D=med11&NEWS=N&AN=24845110

12. Horwood J, Hanratty D, Chandran P, Billings P. Primary closure or rhomboid excision and Limberg flap for the management of primary sacrococcygeal pilonidal disease? A meta-analysis of randomized controlled trials. Colorectal Dis [Internet]. 2012;14(2):143–51. Available from: http://ovidsp.ovid.com/ovidweb.cgi?T=JS&PAGE=reference&D=med9&NEWS=N&AN=20969718

13. Petersen S, Koch R, Stelzner S, Wendlandt T-P, Ludwig K. Primary closure techniques in chronic pilonidal sinus: a survey of the results of different surgical approaches. Dis Colon Rectum [Internet]. 2002;45(11):1458–67. Available from: http://ovidsp.ovid.com/ovidweb.cgi?T=JS&PAGE=reference&D=med4&NEWS=N&AN=12432292

14. J. H, D. H, P. C. Primary suture or limberg flap for primary sacrococcygeal pilonidal disease: Meta analysis of randomised controlled trials (RCT). Color Dis [Internet]. 2010;12(SUPPL. 1):40–1. Available from: http://ovidsp.ovid.com/ovidweb.cgi?T=JS&PAGE=reference&D=emed11&NEWS=N&AN=70236488

15. Arnous M, Elgendy H, Thabet W, Emile SH, Elbaz SA, Khafagy W. Excision with primary midline closure compared with Limberg flap in the treatment of sacrococcygeal pilonidal disease: a randomised clinical trial. Ann R Coll Surg Engl [Internet]. 2019;101(1):21–9. Available from: http://ovidsp.ovid.com/ovidweb.cgi?T=JS&PAGE=reference&D=medc&NEWS=N&AN=30286636

16. Saydam M, Ozturk B, Sinan H, Balta AZ, Demir P, Ozer MT, et al. Comparison of modified Limberg flap transposition and lateral advancement flap transposition with Burow’s triangle in the treatment of pilonidal sinus disease. Am J Surg [Internet]. 2015;210(4):772–7. Available from: http://ovidsp.ovid.com/ovidweb.cgi?T=JS&PAGE=reference&D=med12&NEWS=N&AN=26138521

17. Guner A, Boz A, Ozkan OF, Ileli O, Kece C, Reis E. Limberg flap versus Bascom cleft lift techniques for sacrococcygeal pilonidal sinus: prospective, randomized trial. World J Surg [Internet]. 2013;37(9):2074–80. Available from: http://ovidsp.ovid.com/ovidweb.cgi?T=JS&PAGE=reference&D=med10&NEWS=N&AN=23732258

18. Bessa SS. Comparison of short-term results between the modified Karydakis flap and the modified Limberg flap in the management of pilonidal sinus disease: a randomized controlled study. Dis Colon Rectum [Internet]. 2013;56(4):491–8. Available from: http://ovidsp.ovid.com/ovidweb.cgi?T=JS&PAGE=reference&D=med10&NEWS=N&AN=23478617

19. Okus A, Sevinc B, Karahan O, Eryilmaz MA. Comparison of Limberg flap and tension-free primary closure during pilonidal sinus surgery. World J Surg [Internet]. 2012;36(2):431–5. Available from: http://ovidsp.ovid.com/ovidweb.cgi?T=JS&PAGE=reference&D=med9&NEWS=N&AN=22057753

20. Kirkil C, Boyuk A, Bulbuller N, Aygen E, Karabulut K, Coskun S. The effects of drainage on the rates of early wound complications and recurrences after Limberg flap reconstruction in patients with pilonidal disease. Tech Coloproctol [Internet]. 2011;15(4):425–9. Available from: http://ovidsp.ovid.com/ovidweb.cgi?T=JS&PAGE=reference&D=med8&NEWS=N&AN=22033544

21. Ates M, Dirican A, Sarac M, Aslan A, Colak C. Short and long-term results of the Karydakis flap versus the Limberg flap for treating pilonidal sinus disease: a prospective randomized study. Am J Surg [Internet]. 2011;202(5):568–73. Available from: http://ovidsp.ovid.com/ovidweb.cgi?T=JS&PAGE=reference&D=med8&NEWS=N&AN=21788003

22. Tavassoli A, Noorshafiee S, Nazarzadeh R. Comparison of excision with primary repair versus Limberg flap. Int J Surg [Internet]. 2011;9(4):343–6. Available from: http://ovidsp.ovid.com/ovidweb.cgi?T=JS&PAGE=reference&D=med8&NEWS=N&AN=21354343

23. Muzi MG, Milito G, Cadeddu F, Nigro C, Andreoli F, Amabile D, et al. Randomized comparison of Limberg flap versus modified primary closure for the treatment of pilonidal disease. Am J Surg [Internet]. 2010;200(1):9–14. Available from: http://ovidsp.ovid.com/ovidweb.cgi?T=JS&PAGE=reference&D=med8&NEWS=N&AN=20637332

24. Can MF, Sevinc MM, Hancerliogullari O, Yilmaz M, Yagci G. Multicenter prospective randomized trial comparing modified Limberg flap transposition and Karydakis flap reconstruction in patients with sacrococcygeal pilonidal disease. Am J Surg [Internet]. 2010;200(3):318–27. Available from: http://ovidsp.ovid.com/ovidweb.cgi?T=JS&PAGE=reference&D=med8&NEWS=N&AN=20122682

25. Colak T, Turkmenoglu O, Dag A, Akca T, Aydin S. A randomized clinical study evaluating the need for drainage after Limberg flap for pilonidal sinus. J Surg Res [Internet]. 2010;158(1):127–31. Available from: http://ovidsp.ovid.com/ovidweb.cgi?T=JS&PAGE=reference&D=med8&NEWS=N&AN=19394643

26. Nursal TZ, Ezer A, Caliskan K, Torer N, Belli S, Moray G. Prospective randomized controlled trial comparing V-Y advancement flap with primary suture methods in pilonidal disease. Am J Surg [Internet]. 2010;199(2):170–7. Available from: http://ovidsp.ovid.com/ovidweb.cgi?T=JS&PAGE=reference&D=med8&NEWS=N&AN=19362290

27. Bali I, Aziret M, Sozen S, Emir S, Erdem H, Cetinkunar S, et al. Effectiveness of Limberg and Karydakis flap in recurrent pilonidal sinus disease. Clinics (Sao Paulo) [Internet]. 2015;70(5):350–5. Available from: http://ovidsp.ovid.com/ovidweb.cgi?T=JS&PAGE=reference&D=med12&NEWS=N&AN=26039952

28. Karakayali F, Karagulle E, Karabulut Z, Oksuz E, Moray G, Haberal M. Unroofing and marsupialization vs. rhomboid excision and Limberg flap in pilonidal disease: a prospective, randomized, clinical trial. Dis Colon Rectum [Internet]. 2009;52(3):496–502. Available from: http://ovidsp.ovid.com/ovidweb.cgi?T=JS&PAGE=reference&D=med7&NEWS=N&AN=19333052

29. Jamal A, Shamim M, Hashmi F, Qureshi MI. Open excision with secondary healing versus rhomboid excision with Limberg transposition flap in the management of sacrococcygeal pilonidal disease. J Pak Med Assoc [Internet]. 2009;59(3):157–60. Available from: http://ovidsp.ovid.com/ovidweb.cgi?T=JS&PAGE=reference&D=med7&NEWS=N&AN=19288942

30. Ersoy E, Devay AO, Aktimur R, Doganay B, Ozdogan M, Gundogdu RH. Comparison of the short-term results after Limberg and Karydakis procedures for pilonidal disease: randomized prospective analysis of 100 patients. Colorectal Dis [Internet]. 2009;11(7):705–10. Available from: http://ovidsp.ovid.com/ovidweb.cgi?T=JS&PAGE=reference&D=med7&NEWS=N&AN=18637924

31. Altinli E, Koksal N, Onur E, Celik A, Sumer A. Impact of fibrin sealant on Limberg flap technique: results of a randomized controlled trial. Tech Coloproctol [Internet]. 2007;11(1):22–5. Available from: http://ovidsp.ovid.com/ovidweb.cgi?T=JS&PAGE=reference&D=med6&NEWS=N&AN=17357862

32. Fazeli MS, Adel MG, Lebaschi AH. Comparison of outcomes in Z-plasty and delayed healing by secondary intention of the wound after excision of the sacral pilonidal sinus: results of a randomized, clinical trial. Dis Colon Rectum [Internet]. 2006;49(12):1831–6. Available from: http://ovidsp.ovid.com/ovidweb.cgi?T=JS&PAGE=reference&D=med6&NEWS=N&AN=17080281

33. Cihan A, Ucan BH, Comert M, Cesur A, Cakmak GK, Tascilar O. Superiority of asymmetric modified Limberg flap for surgical treatment of pilonidal disease. Dis Colon Rectum [Internet]. 2006;49(2):244–9. Available from: http://ovidsp.ovid.com/ovidweb.cgi?T=JS&PAGE=reference&D=med6&NEWS=N&AN=16322964

34. Ertan T, Koc M, Gocmen E, Aslar AK, Keskek M, Kilic M. Does technique alter quality of life after pilonidal sinus surgery?. Am J Surg [Internet]. 2005;190(3):388–92. Available from: http://ovidsp.ovid.com/ovidweb.cgi?T=JS&PAGE=reference&D=med6&NEWS=N&AN=16105524

35. Akca T, Colak T, Ustunsoy B, Kanik A, Aydin S. Randomized clinical trial comparing primary closure with the Limberg flap in the treatment of primary sacrococcygeal pilonidal disease. Br J Surg [Internet]. 2005;92(9):1081–4. Available from: http://ovidsp.ovid.com/ovidweb.cgi?T=JS&PAGE=reference&D=med6&NEWS=N&AN=16078300

36. Berkem H, Topaloglu S, Ozel H, Avsar FM, Yildiz Y, Yuksel BC, et al. V-Y advancement flap closures for complicated pilonidal sinus disease. Int J Colorectal Dis [Internet]. 2005;20(4):343–8. Available from: http://ovidsp.ovid.com/ovidweb.cgi?T=JS&PAGE=reference&D=med6&NEWS=N&AN=15747127

37. Abu Galala KH, Salam IM, Abu Samaan KR, El Ashaal YI, Chandran VP, Sabastian M, et al. Treatment of pilonidal sinus by primary closure with a transposed rhomboid flap compared with deep suturing: a prospective randomised clinical trial. Eur J Surg [Internet]. 1999;165(5):468–72. Available from: http://ovidsp.ovid.com/ovidweb.cgi?T=JS&PAGE=reference&D=med4&NEWS=N&AN=10391165

38. Kaser SA, Zengaffinen R, Uhlmann M, Glaser C, Maurer CA. Primary wound closure with a Limberg flap vs. secondary wound healing after excision of a pilonidal sinus: a multicentre randomised controlled study. Int J Colorectal Dis [Internet]. 2015;30(1):97–103. Available from: http://ovidsp.ovid.com/ovidweb.cgi?T=JS&PAGE=reference&D=med12&NEWS=N&AN=25367184

39. Erdem E, Sungurtekin U, Nessar M. Are postoperative drains necessary with the Limberg flap for treatment of pilonidal sinus?. Dis Colon Rectum [Internet]. 1998;41(11):1427–31. Available from: http://ovidsp.ovid.com/ovidweb.cgi?T=JS&PAGE=reference&D=med4&NEWS=N&AN=9823811

40. Hodgson WJ, Greenstein RJ. A comparative study between Z-plasty and incision and drainage or excision with marsupialization for pilonidal sinuses. Surg Gynecol Obstet [Internet]. 1981;153(6):842–4. Available from: http://ovidsp.ovid.com/ovidweb.cgi?T=JS&PAGE=reference&D=med2&NEWS=N&AN=7029758

41. N. N, K.M. A, M.Z. M, F. Z. Comparison of outcome of open excision with secondary healing versus rhomboid excision with limberg transposition flap in the management of sacrococcygeal pilonidal disease. Pakistan J Med Heal Sci [Internet]. 2015;9(3):879–81. Available from: http://pjmhsonline.com/july_sep_2015/pdf/879COM_1.pdf

42. Khan PS, Hayat H, Hayat G. Limberg Flap Versus Primary Closure in the Treatment of Primary Sacrococcygeal Pilonidal Disease; A Randomized Clinical Trial. Indian J Surg [Internet]. 2013 Jun 16;75(3):192–4. Available from: http://search.ebscohost.com/login.aspx?direct=true&db=cin20&AN=104186474&site=ehost-live

43. Caliskan M, Kosmaz K, Subasi IE, Acar A, Evren I, Bas G, et al. Comparison of Common Surgical Procedures in Non-complicated Pilonidal Sinus Disease, a 7-Year Follow-Up Trial. World J Surg [Internet]. 2020 Apr 17;44(4):1091–8. Available from: http://ovidsp.ovid.com/ovidweb.cgi?T=JS&PAGE=reference&D=emexb&NEWS=N&AN=630306516

44. A. R, A. A. Comparison of Limberg flap with karydakis repair in pilonidal sinus disease. Med Forum Mon [Internet]. 2019;30(10):91–5. Available from: http://medforum.pk/images/pdf/2019/october2019.pdf

45. K.J. K, A. G, S. C. Comparison of early outcome between modified limberg and karydakis flap procedures in patients with sacrococcygeal pilonidal sinus. Pakistan J Med Heal Sci [Internet]. 2016;10(2):631–4. Available from: http://pjmhsonline.com/2016/april_june/pdf/631.pdf

46. Jabbar MS, Bhutta MM, Puri N. Comparison between primary closure with Limberg Flap versus open procedure in treatment of pilonidal sinus, in terms of frequency of post-operative wound infection. Pakistan J Med Sci [Internet]. 2018 Jan 16;34(1):49–53. Available from: http://www.pjms.com.pk/index.php/pjms/article/download/13929/5865

47. Shabbir F, Ayyaz M, Farooka MW, Toor AA, Sarwar H, Malik AA. Modified Limberg’s flap versus primary closure for treatment of pilonidal sinus disease: a comparative study. J Pak Med Assoc [Internet]. 2014;64(11):1270–3. Available from: http://ovidsp.ovid.com/ovidweb.cgi?T=JS&PAGE=reference&D=med11&NEWS=N&AN=25831644

48. Rashidian N, Vahedian-Ardakani J, Baghai-Wadji M, Keramati MR, Saraee A, Ansari K, et al. How to repair the surgical defect after excision of sacrococcygeal pilonidal sinus: a dilemma. J Wound Care [Internet]. 2014;23(12):630–3. Available from: http://ovidsp.ovid.com/ovidweb.cgi?T=JS&PAGE=reference&D=med11&NEWS=N&AN=25492279

49. Enshaei A, Motearefi S. Comparison of two surgical methods, primary closure and rotational flap, in patients with chronic pilonidal sinus. Glob J Health Sci [Internet]. 2014;6(7 Spec No):18–22. Available from: http://ovidsp.ovid.com/ovidweb.cgi?T=JS&PAGE=reference&D=med11&NEWS=N&AN=25363174

50. Boulanger G, Abet E, Brau-Weber AG, Leclair F, Denimal F, Jean MH, et al. Is histological analysis of pilonidal sinus useful? Retrospective analysis of 731 resections. J Visc Surg [Internet]. 2018;155(3):191–4. Available from: http://ovidsp.ovid.com/ovidweb.cgi?T=JS&PAGE=reference&D=medc&NEWS=N&AN=29146394

51. Das K, Uzun AS, Bozkurt H, Karateke F, Menekse E, Nacar H, et al. Diathermy versus scalpel in Limberg flap in pilonidal sinus surgery. A prospective randomized trial. Ann Ital Chir [Internet]. 2014;85(2):148–52. Available from: http://ovidsp.ovid.com/ovidweb.cgi?T=JS&PAGE=reference&D=med11&NEWS=N&AN=24902075

52. Arslan K, Said Kokcam S, Koksal H, Turan E, Atay A, Dogru O. Which flap method should be preferred for the treatment of pilonidal sinus? A prospective randomized study. Tech Coloproctol [Internet]. 2014;18(1):29–37. Available from: http://ovidsp.ovid.com/ovidweb.cgi?T=JS&PAGE=reference&D=med11&NEWS=N&AN=23430349

53. Sabuncuoglu MZ, Sabuncuoglu A, Dandin O, Benzin MF, Celik G, Sozen I, et al. Eyedrop-shaped, modified Limberg transposition flap in the treatment of pilonidal sinus disease. Asian J Surg [Internet]. 2015;38(3):161–7. Available from: http://ovidsp.ovid.com/ovidweb.cgi?T=JS&PAGE=reference&D=med12&NEWS=N&AN=25912103

54. Afsarlar CE, Yilmaz E, Karaman A, Karaman I, Ozguner IF, Erdogan D, et al. Treatment of adolescent pilonidal disease with a new modification to the Limberg flap: symmetrically rotated rhomboid excision and lateralization of the Limberg flap technique. J Pediatr Surg [Internet]. 2013;48(8):1744–9. Available from: http://ovidsp.ovid.com/ovidweb.cgi?T=JS&PAGE=reference&D=med10&NEWS=N&AN=23932616

55. Koca YS, Yildiz I, Okur SK, Saricik B, Ugur M, Bulbul MT, et al. Comparison of Unilateral Fasciocutaneous V-Y Flap Technique with Cleft Lift Procedure in the Treatment of Recurrent Pilonidal Sinus Disease: A Retrospective Clinical Study. Med Sci Monit [Internet]. 2018;24:711–7. Available from: http://ovidsp.ovid.com/ovidweb.cgi?T=JS&PAGE=reference&D=med15&NEWS=N&AN=29397396

56. Lahooti M, Taheri PA, Nezami BG, Assa S. Sacrococcygeal pilonidal sinus treated by a new fascio-cutaneous flap. Dis Colon Rectum [Internet]. 2008;51(5):588–92. Available from: http://ovidsp.ovid.com/ovidweb.cgi?T=JS&PAGE=reference&D=med7&NEWS=N&AN=18286337

57. Arumugam PJ, Chandrasekaran T V, Morgan AR, Beynon J, Carr ND. The rhomboid flap for pilonidal disease. Colorectal Dis [Internet]. 2003;5(3):218–21. Available from: http://ovidsp.ovid.com/ovidweb.cgi?T=JS&PAGE=reference&D=med5&NEWS=N&AN=12780881

58. Mentes O, Bagci M, Bilgin T, Ozgul O, Ozdemir M. Limberg flap procedure for pilonidal sinus disease: results of 353 patients. Langenbeck’s Arch Surg [Internet]. 2008;393(2):185–9. Available from: http://ovidsp.ovid.com/ovidweb.cgi?T=JS&PAGE=reference&D=med7&NEWS=N&AN=17899165

59. Milito G, Gargiani M, Gallinela MM, Crocoli A, Spyrou M, Farinon AM. Modified Limberg’s transposition flap for pilonidal sinus. Long term follow up of 216 cases. Ann Ital Chir [Internet]. 2007;78(3):227–31. Available from: http://ovidsp.ovid.com/ovidweb.cgi?T=JS&PAGE=reference&D=med6&NEWS=N&AN=17722498

60. De Falco M, Ragusa M, Oliva G, Miranda A, Giudicianni C, Sperlongano P, et al. [Surgical treatment of sinus pilonidalis by Dufourmentel’s flap technique]. Tratt Chir del sinus pilonidalis con trasposizione di lembo Second Dufourmentel [Internet]. 2007;28(3):93–7. Available from: http://ovidsp.ovid.com/ovidweb.cgi?T=JS&PAGE=reference&D=med6&NEWS=N&AN=17419905

61. Turan A, Isler C, Bas SC, Genc B, Ozsoy Z. A new flap for reconstruction of pilonidal sinus: lumbar adipofascial turnover flap. Ann Plast Surg [Internet]. 2007;58(4):411–5. Available from: http://ovidsp.ovid.com/ovidweb.cgi?T=JS&PAGE=reference&D=med6&NEWS=N&AN=17413884

62. Salvi PF, Midiri G, Tucci G, Lombardi A, Coppola M, Conte S, et al. [Rhomboid flap in radical surgical treatment of sacrococcygeal fistulas: technical notes]. lembo di rotazione romboide nel trattameno Chir Radic delle fistole sacrocogginee note di Tec [Internet]. 2006;27(8–9):335–8. Available from: http://ovidsp.ovid.com/ovidweb.cgi?T=JS&PAGE=reference&D=med6&NEWS=N&AN=17064496

63. Faux W, Pillai SCB, Gold DM. Limberg flap for pilonidal disease: the “no-protractor” approach, 3 steps to success. Tech Coloproctol [Internet]. 2005;9(2):153–5. Available from: http://ovidsp.ovid.com/ovidweb.cgi?T=JS&PAGE=reference&D=med6&NEWS=N&AN=16007356

64. Mentes BB, Leventoglu S, Cihan A, Tatlicioglu E, Akin M, Oguz M. Modified Limberg transposition flap for sacrococcygeal pilonidal sinus. Surg Today [Internet]. 2004;34(5):419–23. Available from: http://ovidsp.ovid.com/ovidweb.cgi?T=JS&PAGE=reference&D=med5&NEWS=N&AN=15108080

65. Eryilmaz R, Sahin M, Alimoglu O, Dasiran F. Surgical treatment of sacrococcygeal pilonidal sinus with the Limberg transposition flap. Surgery [Internet]. 2003;134(5):745–9. Available from: http://ovidsp.ovid.com/ovidweb.cgi?T=JS&PAGE=reference&D=med5&NEWS=N&AN=14639351

66. Lebo PB, Dahmann S, Sinkovits E, Meyer-Marcotty M. [Pilonidal sinus: Secondary wound closure vs. Limberg flap : Cost and satisfaction analysis]. Sinus pilonidalis Sekundare Wundheilung vs Limberg-Lappenplastik Eine Zufriedenheits- und Kostenanalyse [Internet]. 2017;88(3):226–32. Available from: http://ovidsp.ovid.com/ovidweb.cgi?T=JS&PAGE=reference&D=med14&NEWS=N&AN=27629695

67. Jaschke CW, Mahrlein R, Mangold G. [Results of the Limberg transposition flap in the treatment of pilonidal sinus]. Ergebnisse der Behandlung des Sinus pilonidalis durch Schwenklappenplastik nach Limberg [Internet]. 2002;127(8):712–5. Available from: http://ovidsp.ovid.com/ovidweb.cgi?T=JS&PAGE=reference&D=med4&NEWS=N&AN=12200736

68. Kapan M, Kapan S, Pekmezci S, Durgun V. Sacrococcygeal pilonidal sinus disease with Limberg flap repair. Tech Coloproctol [Internet]. 2002;6(1):27–32. Available from: http://ovidsp.ovid.com/ovidweb.cgi?T=JS&PAGE=reference&D=med4&NEWS=N&AN=12077638

69. Saray A, Dirlik M, Caglikulekci M, Turkmenoglu O. Gluteal V-Y advancement fasciocutaneous flap for treatment of chronic pilonidal sinus disease. Scand J Plast Reconstr Surg hand Surg [Internet]. 2002;36(2):80–4. Available from: http://ovidsp.ovid.com/ovidweb.cgi?T=JS&PAGE=reference&D=med4&NEWS=N&AN=12038210

70. Urhan MK, Kucukel F, Topgul K, Ozer I, Sari S. Rhomboid excision and Limberg flap for managing pilonidal sinus: results of 102 cases. Dis Colon Rectum [Internet]. 2002;45(5):656–9. Available from: http://ovidsp.ovid.com/ovidweb.cgi?T=JS&PAGE=reference&D=med4&NEWS=N&AN=12004216

71. Jonas J, Blaich S, Bahr R. [The Limberg transposition flap in surgical therapy of chronic pilonidal sinus]. Der Transpositionslappen nach Limb der Oper Ther des chronischen Sinus pilonidalis [Internet]. 2000;125(12):976–81. Available from: http://ovidsp.ovid.com/ovidweb.cgi?T=JS&PAGE=reference&D=med4&NEWS=N&AN=11190616

72. Dylek ON, Bekereciodlu M. Role of simple V-Y advancement flap in the treatment of complicated pilonidal sinus. Eur J Surg [Internet]. 1998;164(12):961–4. Available from: http://ovidsp.ovid.com/ovidweb.cgi?T=JS&PAGE=reference&D=med4&NEWS=N&AN=10029392

73. Milito G, Cortese F, Casciani CU. Rhomboid flap procedure for pilonidal sinus: results from 67 cases. Int J Colorectal Dis [Internet]. 1998;13(3):113–5. Available from: http://ovidsp.ovid.com/ovidweb.cgi?T=JS&PAGE=reference&D=med4&NEWS=N&AN=9689559

74. Hasse FM, Rademacher C, Bingham K, Lohlein D. [The Dufourmentel flap-plasty for treatment of chronic pilonidal sinus]. Die Dufourmentel-Lappenplastik zur Behandlung des chronischen Sinus pilonidalis [Internet]. 1998;69(6):663–6. Available from: http://ovidsp.ovid.com/ovidweb.cgi?T=JS&PAGE=reference&D=med4&NEWS=N&AN=9676371

75. Bozkurt MK, Tezel E. Management of pilonidal sinus with the Limberg flap. Dis Colon Rectum [Internet]. 1998;41(6):775–7. Available from: http://ovidsp.ovid.com/ovidweb.cgi?T=JS&PAGE=reference&D=med4&NEWS=N&AN=9645748

76. Schoeller T, Wechselberger G, Otto A, Papp C. Definite surgical treatment of complicated recurrent pilonidal disease with a modified fasciocutaneous V-Y advancement flap. Surgery [Internet]. 1997;121(3):258–63. Available from: http://ovidsp.ovid.com/ovidweb.cgi?T=JS&PAGE=reference&D=med4&NEWS=N&AN=9068667

77. Oz B, Akcan A, Emek E, Akyuz M, Sozuer E, Akyldiz H, et al. A comparison of surgical outcome of fasciocutaneous V-Y advancement flap and Limberg transposition flap for recurrent sacrococcygeal pilonidal sinus disease. Asian J Surg [Internet]. 2017;40(3):197–202. Available from: http://ovidsp.ovid.com/ovidweb.cgi?T=JS&PAGE=reference&D=med14&NEWS=N&AN=26621004

78. Rosen W, Davidson JS. Gluteus maximus musculocutaneous flap for the treatment of recalcitrant pilonidal disease. Ann Plast Surg [Internet]. 1996;37(3):293–7. Available from: http://ovidsp.ovid.com/ovidweb.cgi?T=JS&PAGE=reference&D=med4&NEWS=N&AN=8883728

79. Ozgultekin R, Ersan Y, Ozcan M, Ozcelik F, Celik V, Cercel A, et al. [Therapy of pilonidal sinus with the Limberg transposition flap]. Die Ther des Sinus pilonidalis mit dem Transpositionslappen nach Limberg [Internet]. 1995;66(3):192–5. Available from: http://ovidsp.ovid.com/ovidweb.cgi?T=JS&PAGE=reference&D=med3&NEWS=N&AN=7750389

80. Khatri VP, Espinosa MH, Amin AK. Management of recurrent pilonidal sinus by simple V-Y fasciocutaneous flap. Dis Colon Rectum [Internet]. 1994;37(12):1232–5. Available from: http://ovidsp.ovid.com/ovidweb.cgi?T=JS&PAGE=reference&D=med3&NEWS=N&AN=7995149

81. Manterola C, Barroso M, Araya JC, Fonseca L. Pilonidal disease: 25 cases treated by the Dufourmentel technique. Dis Colon Rectum [Internet]. 1991;34(8):649–52. Available from: http://ovidsp.ovid.com/ovidweb.cgi?T=JS&PAGE=reference&D=med3&NEWS=N&AN=1855420

82. Jimenez Romero C, Alcalde M, Martin F, Pulido A, Rico P. Treatment of pilonidal sinus by excision and rhomboid flap. Int J Colorectal Dis [Internet]. 1990;5(4):200–2. Available from: http://ovidsp.ovid.com/ovidweb.cgi?T=JS&PAGE=reference&D=med3&NEWS=N&AN=2286802

83. Tschudi J, Ris HB. [Morbidity of Z-plasty in the treatment of pilonidal sinus]. Die Morb der Z-Plastik der Behandlung des Pilonidalsinus [Internet]. 1988;59(7):486–90. Available from: http://ovidsp.ovid.com/ovidweb.cgi?T=JS&PAGE=reference&D=med3&NEWS=N&AN=3063455

84. Gwynn BR. Use of the rhomboid flap in pilonidal sinus. Ann R Coll Surg Engl [Internet]. 1986;68(1):40–1. Available from: http://ovidsp.ovid.com/ovidweb.cgi?T=JS&PAGE=reference&D=med2&NEWS=N&AN=3947013

85. Azab AS, Kamal MS, el Bassyoni F. The rationale of using the rhomboid fasciocutaneous transposition flap for the radical cure of pilonidal sinus. J Dermatol Surg Oncol [Internet]. 1986;12(12):1295–9. Available from: http://ovidsp.ovid.com/ovidweb.cgi?T=JS&PAGE=reference&D=med2&NEWS=N&AN=3782603

86. Sabiani P, Le Treut YP, Bouloudnine G, Maillet B, Bricot R. [Treatment of pilonidal disease using an LLL-plasty technic. Apropos of 27 cases]. Le Trait la Mal pilonidale par la Tech d’exerese-plastie “LLL” A Propos 27 cas [Internet]. 1985;21(5):287–90. Available from: http://ovidsp.ovid.com/ovidweb.cgi?T=JS&PAGE=reference&D=med2&NEWS=N&AN=4083815

87. Azab AS, Kamal MS, Saad RA, Abou al Atta KA, Ali NA. Radical cure of pilonidal sinus by a transposition rhomboid flap. Br J Surg [Internet]. 1984;71(2):154–5. Available from: http://ovidsp.ovid.com/ovidweb.cgi?T=JS&PAGE=reference&D=med2&NEWS=N&AN=6692112

88. Bayhan Z, Zeren S, Duzgun SA, Ucar BI, Alparslan Yumun HN, Mestan M. Crystallized phenol application and modified Limberg flap procedure in treatment of pilonidal sinus disease: A comparative retrospective study. Asian J Surg [Internet]. 2016;39(3):172–7. Available from: http://ovidsp.ovid.com/ovidweb.cgi?T=JS&PAGE=reference&D=med13&NEWS=N&AN=26883556

89. Mansoory A, Dickson D. Z-plasty for treatment of disease of the pilonidal sinus. Surg Gynecol Obstet [Internet]. 1982;155(3):409–11. Available from: http://ovidsp.ovid.com/ovidweb.cgi?T=JS&PAGE=reference&D=med2&NEWS=N&AN=7112368

90. Fishbein RH, Handelsman JC. A method for primary reconstruction following radical excision of sacrococcygeal pilonidal disease. Ann Surg [Internet]. 1979;190(2):231–5. Available from: http://ovidsp.ovid.com/ovidweb.cgi?T=JS&PAGE=reference&D=med1&NEWS=N&AN=380484

91. Stroosma OC. Gluteal fasciaplasty as a method of primary closure in the treatment of pilonidal sinus. Arch Chir Neerl [Internet]. 1978;30(1):61–4. Available from: http://ovidsp.ovid.com/ovidweb.cgi?T=JS&PAGE=reference&D=med1&NEWS=N&AN=655733

92. Sood SC, Green JR, Parui R. Results of various operations for sacrococcygeal pilonidal disease. Plast Reconstr Surg [Internet]. 1975;56(5):559–66. Available from: http://ovidsp.ovid.com/ovidweb.cgi?T=JS&PAGE=reference&D=med1&NEWS=N&AN=1187884

93. Bose B, Candy J. Radical cure of pilonidal sinus by Z-plasty. Am J Surg [Internet]. 1970;120(6):783–6. Available from: http://ovidsp.ovid.com/ovidweb.cgi?T=JS&PAGE=reference&D=med1&NEWS=N&AN=5488333

94. Ozcan B, Ilkgul O. Contralateral Limberg flap reconstruction for pilonidal disease recurrence. Asian J Surg [Internet]. 2019;42(8):787–91. Available from: http://ovidsp.ovid.com/ovidweb.cgi?T=JS&PAGE=reference&D=medl&NEWS=N&AN=30711442

95. Pomazkin VI. [Modified Limberg plasty in treatment of epithelial coccygeal passage]. Khirurgiia (Sofiia) [Internet]. 2015;(5):51–5. Available from: http://ovidsp.ovid.com/ovidweb.cgi?T=JS&PAGE=reference&D=med12&NEWS=N&AN=26271324

96. Sungur N, Kocer U, Uysal A, Arslan C, Cologlu H, Ulusoy G. V-Y rotation advancement fasciocutaneous flap for excisional defects of pilonidal sinus. Plast Reconstr Surg [Internet]. 2006;117(7):2448–54. Available from: http://ovidsp.ovid.com/ovidweb.cgi?T=JS&PAGE=reference&D=med6&NEWS=N&AN=16772955

97. Tuncbilek G, Nasir S, Ozkan O, Kayikcioglu A, Mavili E. Partially de-epithelialised and buried V-Y advancement flap for reconstruction of sacrococcygeal and ischial defects. Scand J Plast Reconstr Surg hand Surg [Internet]. 2004;38(2):94–9. Available from: http://ovidsp.ovid.com/ovidweb.cgi?T=JS&PAGE=reference&D=med5&NEWS=N&AN=15202666

98. Nessar G, Kayaalp C, Seven C. Elliptical rotation flap for pilonidal sinus. Am J Surg [Internet]. 2004;187(2):300–3. Available from: http://ovidsp.ovid.com/ovidweb.cgi?T=JS&PAGE=reference&D=med5&NEWS=N&AN=14769325

99. Dahmann S, Lebo PB, Meyer-Marcotty M V. [Comparison of Treatments for an Infected Pilonidal Sinus: Differences in Scar Quality and Outcome Between Secondary Wound Healing and Limberg Flap in a Prospective Study]. Ther bei infiziertem Sinus pilonidalis - Unterschiede von Narbenqualitat und Outcome nach Sekundarheilung oder Limberglappenplastik im Rahmen einer prospektiven Stud [Internet]. 2016;48(2):111–9. Available from: http://ovidsp.ovid.com/ovidweb.cgi?T=JS&PAGE=reference&D=med13&NEWS=N&AN=27096210

100. HIRSHOWITZ B, WEXLER MR. THE TREATMENT OF PILONIDAL SINUS: PLASTIC SURGERY APPROACH. Plast Reconstr Surg [Internet]. 1964;34:521–8. Available from: http://ovidsp.ovid.com/ovidweb.cgi?T=JS&PAGE=reference&D=med1&NEWS=N&AN=14223253

101. DWIGHT RW. Pilonidal sinus; an evaluation of plastic closure using flaps of gluteal muscle. AMA Arch Surg [Internet]. 1952;64(4):438–42. Available from: http://ovidsp.ovid.com/ovidweb.cgi?T=JS&PAGE=reference&D=med1&NEWS=N&AN=14902244

102. Nelson R, Lalonde D. Treatment of the chronic pilonidal sinus wound with a local perforator-assisted transposition flap. Plast Reconstr Surg [Internet]. 2008;122(1):47e-49e. Available from: http://ovidsp.ovid.com/ovidweb.cgi?T=JS&PAGE=reference&D=med7&NEWS=N&AN=18594381

103. Garrido A, Ali R, Ramakrishnan V, Spyrou G, Stanley PRW. Reconstruction of the natal cleft with a perforator-based flap. Br J Plast Surg [Internet]. 2002;55(8):671–4. Available from: http://ovidsp.ovid.com/ovidweb.cgi?T=JS&PAGE=reference&D=med4&NEWS=N&AN=12550122

104. Onishi K, Maruyama Y. Sacral adipofascial turn-over flap for the excisional defect of pilonidal sinus. Plast Reconstr Surg [Internet]. 2001;108(7):2006–10. Available from: http://ovidsp.ovid.com/ovidweb.cgi?T=JS&PAGE=reference&D=med4&NEWS=N&AN=11743392

105. Roth RF, Moorman WL. Treatment of pilonidal sinus and cyst by conservative excision and W-plasty closure. Plast Reconstr Surg [Internet]. 1977;60(3):412–5. Available from: http://ovidsp.ovid.com/ovidweb.cgi?T=JS&PAGE=reference&D=med1&NEWS=N&AN=896998

106. Lee HC, Ho YH, Seow CF, Eu KW, Nyam D. Pilonidal disease in Singapore: clinical features and management. Aust N Z J Surg [Internet]. 2000;70(3):196–8. Available from: http://ovidsp.ovid.com/ovidweb.cgi?T=JS&PAGE=reference&D=med4&NEWS=N&AN=10765903

107. Akin M, Gokbayir H, Kilic K, Topgul K, Ozdemir E, Ferahkose Z. Rhomboid excision and Limberg flap for managing pilonidal sinus: long-term results in 411 patients. Colorectal Dis [Internet]. 2008;10(9):945–8. Available from: http://ovidsp.ovid.com/ovidweb.cgi?T=JS&PAGE=reference&D=med7&NEWS=N&AN=18462233

108. H. O, Z.U. O, M. S, I.T. S. Is remaining natal cleft responsible for recurrences in pilonidal disease. Eur Surg Res [Internet]. 2013;50(SUPPL. 1):95. Available from: http://ovidsp.ovid.com/ovidweb.cgi?T=JS&PAGE=reference&D=emed14&NEWS=N&AN=71094732

109. E. D, A. M, A. A. Socioeconomic cost of elective surgery for pilonidal disease in a district general hospital. Color Dis [Internet]. 2017;19(Supplement 4):63. Available from: http://ovidsp.ovid.com/ovidweb.cgi?T=JS&PAGE=reference&D=emed18&NEWS=N&AN=618722341

110. Duman K, Ozdemir Y, Yucel E, Akin ML. Comparison of depression, anxiety and long-term quality of health in patients with a history of either primary closure or Limberg flap reconstruction for pilonidal sinus. Clinics (Sao Paulo) [Internet]. 2014;69(6):384–7. Available from: http://ovidsp.ovid.com/ovidweb.cgi?T=JS&PAGE=reference&D=med11&NEWS=N&AN=24964301

111. K. S, N. G, H. Z, N. Z. Patterns of recurrence of pilonidal cyst after excision and reconstruction with rhomboid Limberg flap. Color Dis [Internet]. 2019;21(Supplement 3):121. Available from: http://ovidsp.ovid.com/ovidweb.cgi?T=JS&PAGE=reference&D=emexb&NEWS=N&AN=631603846

112. S.R. A, S.M. S, A.A. R. Modified limberg’s flap or excision and primary closure what should one choose: Our experience at tertiary care hospital, Srinagar. JK Sci [Internet]. 2018;20(2):95–9. Available from: http://www.jkscience.org/archives/volume202/10-Original

113. Sahsamanis G, Samaras S, Mitsopoulos G, Deverakis T, Dimitrakopoulos G, Pinialidis D. Semi-closed surgical technique for treatment of pilonidal sinus disease. Ann Med Surg [Internet]. 2017 Mar;15:47–51. Available from: http://www.elsevier.com/journals/annals-of-medicine-and-surgery/2049-0801

114. Gezer HÖ, Ezer SS, İnce E, Temiz A. Treatment of young patients with pilonidal sinus disease with the original (unmodified) Limberg flap standardized for the first time. Pediatr Int [Internet]. 2020 Oct 21;62(10):1171–6. Available from: http://ovidsp.ovid.com/ovidweb.cgi?T=JS&PAGE=reference&D=emexb&NEWS=N&AN=631803482

115. Sarı R, Akbaba S, Gündoğdu RH, Yazıcıoğlu MÖ. Comparison of the V-Y Flap and Limberg Flap Operations in Pilonidal Sinus Surgery. Turkish J Color Dis [Internet]. 2019 Jun 1;29(2):69–74. Available from: http://search.ebscohost.com/login.aspx?direct=true&db=cin20&AN=136795166&site=ehost-live

116. Alvandipour M, Zamani MS, Ghorbani M, Charati JY, Karami MY. Comparison of Limberg Flap and Karydakis Flap Surgery for the Treatment of Patients With Pilonidal Sinus Disease: A Single-Blinded Parallel Randomized Study. Ann Coloproctol [Internet]. 2019 Dec 31;35(6):313–8. Available from: https://coloproctol.org/journal/view.php?number=1623

117. A. A, B. O, H. U, M. A, H. A, E. S. A comparison of surgical outcome of fasciocutaneous V-Y advancement flap and limberg transposition flap for complicated sacrococcygeal pilonidal sinus disease. Eur Surg Res [Internet]. 2013;50(SUPPL. 1):137. Available from: http://ovidsp.ovid.com/ovidweb.cgi?T=JS&PAGE=reference&D=emed14&NEWS=N&AN=71094837

118. Ozdemir H, Unal Ozdemir Z, Sunamak O. Complete natal cleft removal with kite incision in the treatment of extensive sacrococcygeal pilonidal sinus. ANZ J Surg [Internet]. 2020 Apr 7;90(4):533–7. Available from: http://ovidsp.ovid.com/ovidweb.cgi?T=JS&PAGE=reference&D=emexb&NEWS=N&AN=629802454

119. E. K, C. R. Total excision with fascial mobilization and primary closure of non-infected pilonidal fistulas. Eur Surg - Acta Chir Austriaca [Internet]. 2012;44(SUPPL. 245):11–2. Available from: http://ovidsp.ovid.com/ovidweb.cgi?T=JS&PAGE=reference&D=emed13&NEWS=N&AN=71644806

120. Şentürk M, Yavuz Y. In Patients with Recurrent Pilonidal Sinus, Should the First Approach be Crystallised Phenol or Limberg Flap? Turkish J Color Dis [Internet]. 2020 Jun 10;30(2):112–6. Available from: http://search.ebscohost.com/login.aspx?direct=true&db=cin20&AN=144395531&site=ehost-live

121. Yabanoglu H, Karagulle E, Belli S, Turk E. Results of modified Dufourmentel rhomboid flap in patients with extensive Sacrococcygeal pilonidal disease. Acta Chir Belg [Internet]. 2014;114(1):52–7. Available from: http://ovidsp.ovid.com/ovidweb.cgi?T=JS&PAGE=reference&D=med11&NEWS=N&AN=24720139

122. Azizi R, Alemrajabi M, Naderan M, Shoar S. Efficacy of modified Limberg flap in surgical treatment of infected pilonidal abscess: a case–control study. Eur Surg [Internet]. 2014 Aug 23;46(4):144–7. Available from: http://www.springerlink.com/content/1682-8631

123. M. A, M. A. Comparison between efficacy of Limberg flap in treatment of infected pilonidal abscess and simple pilonidal fistula. Color Dis [Internet]. 2012;14(SUPPL. 2):33. Available from: http://ovidsp.ovid.com/ovidweb.cgi?T=JS&PAGE=reference&D=emed13&NEWS=N&AN=70926302

124. Carti EB. Is a flap procedure necessary for every pilonidal sinus case? Ann Clin Anal Med [Internet]. 2019 Sep 1;10(5):541–3. Available from: http://www.bayrakol.org/en/2019/september2019/originalarticle/item/download/2011_8e1cb38a44cd7ef564396f6a3fd9a81b

125. A. F. Analysis of 171 consecutive patients with sacrococcygeal pilonidal disease. Dis Colon Rectum [Internet]. 2016;59(5):e140. Available from: http://ovidsp.ovid.com/ovidweb.cgi?T=JS&PAGE=reference&D=emed17&NEWS=N&AN=72308548

126. Yucel E, Tezcan L, Yilmaz OC, Akin ML. “Flag Excision and Flap” Procedure: a Novel Modification for Off-Midline Closure After Pilonidal Sinus Excision. Indian J Surg [Internet]. 2015 Dec 7;77(S3):1191–5. Available from: http://search.ebscohost.com/login.aspx?direct=true&db=cin20&AN=117360734&site=ehost-live

127. S.H. Y. Pilonidal sinus disease: Comparison of outcomes between different surgical techniques. Color Dis [Internet]. 2019;21(Supplement 2):52–3. Available from: http://ovidsp.ovid.com/ovidweb.cgi?T=JS&PAGE=reference&D=emexb&NEWS=N&AN=631602645

128. Volkan Tumay L, Serhat Guner O, Gurluler E. Comparison of classical versus modified Limberg flap techniques in pilonidal sinus surgery. Chirurgia (Bucur) [Internet]. 2020 Mar;33(1):30–5. Available from: https://www.minervamedica.it/en/journals/chirurgia/article.php?cod=R20Y2020N01A0030

129. Galal Elshazly W, Said K. Clinical trial comparing excision and primary closure with modified Limberg flap in the treatment of uncomplicated sacrococcygeal pilonidal disease. Alexandria J Med [Internet]. 2012 Mar 1;48(1):13–8. Available from: http://ovidsp.ovid.com/ovidweb.cgi?T=JS&PAGE=reference&D=emed13&NEWS=N&AN=364207084

130. Rao J, Deora H, Mandia R. A Retrospective Study of 40 Cases of Pilonidal Sinus with Excision of Tract and Z-plasty as Treatment of Choice for Both Primary and Recurrent Cases. Indian J Surg [Internet]. 2015 Dec 2;77(S2):691–3. Available from: http://search.ebscohost.com/login.aspx?direct=true&db=cin20&AN=111984480&site=ehost-live

131. G. D, Y.N. Y, U. G, M. A. Comparison of primary closure and rhomboid flap transposition techniques in the treatment of pilonidal sinus disease. Turkish J Surg [Internet]. 2004;20(3):127–31. Available from: http://www.turkjsurg.com/

132. Yildar M, Cavdar F. Comparison of the Limberg flap and bilateral gluteus maximus advancing flap following oblique excision for the treatment of pilonidal sinus disease. Surg Today [Internet]. 2014;44(10):1828–33. Available from: http://ovidsp.ovid.com/ovidweb.cgi?T=JS&PAGE=reference&D=med11&NEWS=N&AN=24150098

133. Z.A. S, K.H. Q. Rhomboid flap in the treatment of pilonidal sinus disease. Med Forum Mon [Internet]. 2012;23(11):99–103. Available from: http://ovidsp.ovid.com/ovidweb.cgi?T=JS&PAGE=reference&D=emed13&NEWS=N&AN=368163243

134. E. Z, M. T, A.C. Y, M. Z, F. C, M. D. The use of the triangular closure technique for defect coverage in pilonidal sinus treatment. Int J Clin Exp Med [Internet]. 2016;9(7):12804–10. Available from: http://www.ijcem.com/files/ijcem0018562.pdf

135. Limberg flap versus bascom cleft lift techniques for pilonidal sinus. Eur Surg Res [Internet]. 2014;52(3–4):176. Available from: http://ovidsp.ovid.com/ovidweb.cgi?T=JS&PAGE=reference&D=emed15&NEWS=N&AN=71493356

136. T. P, A. O, E.A. M, B. U, C. A. Orifice location guided excision and flap procedures for treatment of sacrococcygeal pilonidal disease. Adv Clin Exp Med [Internet]. 2011;20(4):481–8. Available from: http://ovidsp.ovid.com/ovidweb.cgi?T=JS&PAGE=reference&D=emed12&NEWS=N&AN=362633640

137. J.M. P, K. I, N. L, C.R. R, M.B. L. Retrospective review of recurrence of pilonidal disease after treatment with limberg flap versus other surgical management. Dis Colon Rectum [Internet]. 2018;61(5):e243. Available from: http://ovidsp.ovid.com/ovidweb.cgi?T=JS&PAGE=reference&D=emed19&NEWS=N&AN=622082321

138. Casula G, Uccheddu A, Figus M. [Long term results of the Z-plasty method in the treatment of pilonidal sinus]. Minerva Chir [Internet]. 1983 May 15;38(9):617–20. Available from: http://ovidsp.ovid.com/ovidweb.cgi?T=JS&PAGE=reference&D=emed3&NEWS=N&AN=13068559

139. Ahmadinejad M, Ahmadi K, Ahmadinejad I, Masoud Hashemian A, Khademhoseini P. A Comparison between the Tie-over and Closed Suction Drainage Therapeutic Strategies in Patients Suffering from Sacral Pilonidal Sinus. Int J Biomed Sci [Internet]. 2016 Dec;12(4):149–54. Available from: http://www.ijbs.org/User/ContentFullText.aspx?VolumeNO=12&StartPage=149&Type=pdf

140. M.L. A, L. T, C. Y. “Flag excision and flap” procedure: A novel modification for off-midline closure after pilonidal sinus excision. Color Dis [Internet]. 2011;13(SUPPL. 6):45–6. Available from: http://ovidsp.ovid.com/ovidweb.cgi?T=JS&PAGE=reference&D=emed12&NEWS=N&AN=70567336

141. I. T, D. S, Y. K, D. M. Gluteus maximus fascia plasty flap allows minimising recurrence after excision of pilonidal sinus. Color Dis [Internet]. 2017;19(Supplement 2):137–8. Available from: http://ovidsp.ovid.com/ovidweb.cgi?T=JS&PAGE=reference&D=emed18&NEWS=N&AN=618607427

142. G. G, A. S, M. M, C. T, B. B. Complete excision and primary closure using flap reconstruction for recurrent and chronic pilonidal sinus: 5-year follow-up. Dis Colon Rectum [Internet]. 2013;56(4):e159. Available from: http://ovidsp.ovid.com/ovidweb.cgi?T=JS&PAGE=reference&D=emed14&NEWS=N&AN=71045605

143. Sit M, Aktas G, Yilmaz EE. Comparison of the three surgical flap techniques in pilonidal sinus surgery. Am Surg [Internet]. 2013;79(12):1263–8. Available from: http://ovidsp.ovid.com/ovidweb.cgi?T=JS&PAGE=reference&D=med10&NEWS=N&AN=24351353

144. L. A, T. B. An experience of limberg flap for pilonidal sinus. Pakistan J Med Heal Sci [Internet]. 2015;9(3):923–5. Available from: http://pjmhsonline.com/july_sep_2015/pdf/923

145. M. O, H. S, M. S, H.K. S, E. D, A.Z. B. Early period analysis of 310 patients treated with modified limberg flap method. Eur Surg Res [Internet]. 2013;50(SUPPL. 1):73. Available from: http://ovidsp.ovid.com/ovidweb.cgi?T=JS&PAGE=reference&D=emed14&NEWS=N&AN=71094674

146. S. B, A. L. Reconstructive plastic surgery for complex pilonidal sinuses. Tech Coloproctol [Internet]. 2014;18(4):416. Available from: http://ovidsp.ovid.com/ovidweb.cgi?T=JS&PAGE=reference&D=emed15&NEWS=N&AN=71437570

147. T. H, Y. E, N. S, A. K, M. A-G. Excision of pilonidal sinus and repair with rhomboid flap - Our experience. Tech Coloproctol [Internet]. 2010;14(1):84. Available from: http://ovidsp.ovid.com/ovidweb.cgi?T=JS&PAGE=reference&D=emed11&NEWS=N&AN=70127337

148. H.K. S. V-Y advancement flap versus primary midline closure in chronic pilonidal disease. Eur Surg Res [Internet]. 2013;50(SUPPL. 1):86–7. Available from: http://ovidsp.ovid.com/ovidweb.cgi?T=JS&PAGE=reference&D=emed14&NEWS=N&AN=71094709

149. NCT04017260. Combined Open and Closed Approach for Management of Pilonidal Sinus by Special U-shaped Sutures Without Drain. https://clinicaltrials.gov/show/NCT04017260 [Internet]. 2019; Available from: https://www.cochranelibrary.com/central/doi/10.1002/central/CN-01953259/full

150. Comparison between bascom cleft lift procedure and modified limberg flap reconstruction in the management of complex or recurrent pilonidal disease. Tumori [Internet]. 2019;105(3 Supplement):13–4. Available from: http://ovidsp.ovid.com/ovidweb.cgi?T=JS&PAGE=reference&D=emexa&NEWS=N&AN=629085369

151. Semerdzhiev A, Marinov K. [Our experience in the treatment of epithelial coccygeal cysts and fistulas with rotation flap method of Hirchowitz-Ron Wexler]. Khirurgiia (Sofiia) [Internet]. 1974;27(1):69–73. Available from: http://ovidsp.ovid.com/ovidweb.cgi?T=JS&PAGE=reference&D=emed2&NEWS=N&AN=5095415

152. B. T. Retrospective review of limberg flap versus other surgical management of pilonidal diseases in active duty military personnel. Dis Colon Rectum [Internet]. 2016;59(5):e146--e147. Available from: http://ovidsp.ovid.com/ovidweb.cgi?T=JS&PAGE=reference&D=emed17&NEWS=N&AN=72308560

153. Dass TA, Zaz M, Rather A, Bari S. Elliptical Excision with Midline Primary Closure Versus Rhomboid Excision with Limberg Flap Reconstruction in Sacrococcygeal Pilonidal Disease: A Prospective, Randomized Study. Indian J Surg [Internet]. 2012 Aug 7;74(4):305–8. Available from: http://search.ebscohost.com/login.aspx?direct=true&db=cin20&AN=104416482&site=ehost-live

154. Osmanoglu G, Yetisir F. Limberg flap is better for the surgical treatment of pilonidal sinus. Results of a 767 patients series with an at least five years follow-up period. Chirurgia (Bucur) [Internet]. 2011;106(4):491–4. Available from: http://ovidsp.ovid.com/ovidweb.cgi?T=JS&PAGE=reference&D=med8&NEWS=N&AN=21991875

155. S. J, F. R, M. T. Clinical and therapeutic aspects of the pilonidal sinus in a moroccan population: A retrospective study about 234 cases. Turkish J Gastroenterol [Internet]. 2019;30(Supplement 3):S657. Available from: https://www.turkjgastroenterol.org/en/proceedings-of-the-world-congress-of-gastroenterology-1627423

156. Yang Y, Yu L, Wang Y, Shi J, Li J, Shang F, et al. Comparative analysis on the effect of Z‐plasty versus conventional simple excision for the treatment of sacrococcygeal pilonidal sinus: A retrospective randomised clinical study. Int Wound J [Internet]. 2020 Jun 23;17(3):555–61. Available from: http://search.ebscohost.com/login.aspx?direct=true&db=cin20&AN=142926232&site=ehost-live

157. N. AT, B. AT. Comparative study for treatment of sacrococcygeal pilonidal sinus with simple wide excision versus limberg flap. Pakistan J Med Heal Sci [Internet]. 2018;12(3):911–3. Available from: http://pjmhsonline.com/2018/july_sep/pdf/911.pdf

158. S. S, O. T, M. K, M. T, O. O. The use of fibrin glue in surgical treatment of pilonidal sinus disease: A prospective study in the limberg flap procedure. Pakistan J Med Sci [Internet]. 2011;27(3):537–40. Available from: http://pjms.com.pk/index.php/pjms/article/view/544/173

159. A. K, K. S, Z. K, A. S, I. K. Postoperative wound closure using flaps after excision of pilonidal sinus. Color Dis [Internet]. 2009;11(SUPPL. 2):61. Available from: http://ovidsp.ovid.com/ovidweb.cgi?T=JS&PAGE=reference&D=emed11&NEWS=N&AN=70204793

160. T.B. R, K.L. P, S.L. W, N.E. B. Outcomes following Limberg flap surgery for complex primary or recurrent pilonidal sinus disease at a large teaching hospital: A retrospective review of 123 consecutive cases. Color Dis [Internet]. 2018;20(Supplement 7):51. Available from: http://ovidsp.ovid.com/ovidweb.cgi?T=JS&PAGE=reference&D=emed19&NEWS=N&AN=626682989

161. A. M, K. A, M. T, S. B. Surgical treatment of pilonidal sinus in a district general hospital: Review of surgical procedures. Br J Surg [Internet]. 2019;106(Supplement 5):112. Available from: http://ovidsp.ovid.com/ovidweb.cgi?T=JS&PAGE=reference&D=emexb&NEWS=N&AN=631722266

162. Sinnott CJ, Glickman LT. Limberg flap reconstruction for sacrococcygeal pilonidal sinus disease with and without acute abscess: Our experience and a review of the literature. Arch Plast Surg [Internet]. 2019 May 15;46(3):235–40. Available from: http://www.e-aps.org/upload/pdf/aps-2018-01312.pdf

163. I. E, S. Y, H. Y, H. A. Which rotation could you prefer in surgical intervention? Comparison of limberg flap and karydakis flap techniques in pilonidal sinus disease. Eur Surg - Acta Chir Austriaca [Internet]. 2015;47(SUPPL. 1):S240. Available from: http://ovidsp.ovid.com/ovidweb.cgi?T=JS&PAGE=reference&D=emed16&NEWS=N&AN=71913986

164. Sahasrabudhe P, Panse N, Waghmare C, Waykole P. V-Y Advancement Flap Technique in Resurfacing Postexcisional Defect in Cases with Pilonidal Sinus Disease—Study of 25 Cases. Indian J Surg [Internet]. 2012 Oct 19;74(5):364–70. Available from: http://search.ebscohost.com/login.aspx?direct=true&db=cin20&AN=104432498&site=ehost-live

165. El-Shaer WM. The modified gluteal sliding plication closure in the treatment of chronic pilonidal sinus. Int J Colorectal Dis [Internet]. 2010;25(7):887–94. Available from: http://ovidsp.ovid.com/ovidweb.cgi?T=JS&PAGE=reference&D=med8&NEWS=N&AN=20221765

166. Saylam B, Balli DN, Duzgun AP, Ozer MV, Coskun F. Which surgical procedure offers the best treatment for pilonidal disease?. Langenbeck’s Arch Surg [Internet]. 2011;396(5):651–8. Available from: http://ovidsp.ovid.com/ovidweb.cgi?T=JS&PAGE=reference&D=med8&NEWS=N&AN=21384188

167. Farag A, Nasr SE, Farag AA, Elbarmelgi MY. The Use of Paraspinal Transposition Flap for Recurrent Pilonidal Sinus, a New Histological Basis for Management of Pilonidal Sinus Disease. Indian J Surg [Internet]. 2020 Aug 11;82(4):514–9. Available from: http://search.ebscohost.com/login.aspx?direct=true&db=cin20&AN=144745945&site=ehost-live

168. Gemici K, Şentürk S. Surgical Treatment of Expansive Sacrococcygeal Pilonidal Sinus with the Spider Procedure. Eur J Gen Med [Internet]. 2015 Oct;12(3):203–7. Available from: http://search.ebscohost.com/login.aspx?direct=true&db=cin20&AN=110787548&site=ehost-live

169. K. M. Limberg flap reconstruction for treatment of recurrent pilonidal sinus disease: Is it time to declare victory? Dis Colon Rectum [Internet]. 2009;52(4):858. Available from: http://ovidsp.ovid.com/ovidweb.cgi?T=JS&PAGE=reference&D=emed11&NEWS=N&AN=70343003

170. R. Z, C. S, A. L, M. U, S. K, A. A, et al. Bilateral gluteal fasciocutaneous advancement flaps with and without compressing tie-over sutures in treatment of recurrent pilonidal disease. Dis Colon Rectum [Internet]. 2019;62(6):e282--e283. Available from: http://ovidsp.ovid.com/ovidweb.cgi?T=JS&PAGE=reference&D=emexa&NEWS=N&AN=628086752

171. Kokosis G, Barbas A, Ong C, Levinson H, Erdmann D, Mantyh CR. Flap repair of complex pilonidal sinus: a single institution experience. Eur J Plast Surg [Internet]. 2018 Apr 16;41(2):217–22. Available from: http://link.springer.de/link/service/journals/00238/index.htm

172. I. T, D. S, Y. K, M. Z, D. M. Usage of mobilised fascial flaps in plastic reconstructions after primary pilonidal sinus excision-a modified surgical technique. Color Dis [Internet]. 2019;21(Supplement 3):117. Available from: http://ovidsp.ovid.com/ovidweb.cgi?T=JS&PAGE=reference&D=emexb&NEWS=N&AN=631602227

173. Faruk MO, Sheikh MSH, Parvin MM, Siddiquee MA, Bhuiyan MJH, Aziz MM. Limberg Flap Reconstruction in Treating Sacrococcygeal Pilonidal Sinus. Bangladesh J Med Sci [Internet]. 2019 Nov 3;19(1):105–9. Available from: https://www.banglajol.info/index.php/BJMS/article/download/43881/32384

174. TERUYA M, YAMAMOTO Y, UEZATO H, NONAKA S. A Statistical Survey of Pilonidal Sinus at the Department of Dermatology, Faculty of Medecine, University of the Ryukyus. Nishi Nihon Hifuka [Internet]. 2003;65(6):595–8. Available from: http://ovidsp.ovid.com/ovidweb.cgi?T=JS&PAGE=reference&D=emed8&NEWS=N&AN=38036169

175. K.H. AG, I.M.A. S, Y.I. EA, V.P. C. Excision of pilonidal sinus and primary closure by a rhomboid flap transposition. Asian J Surg [Internet]. 1996;19(4):305–8. Available from: http://ovidsp.ovid.com/ovidweb.cgi?T=JS&PAGE=reference&D=emed6&NEWS=N&AN=27032713

176. O. U, E. I, T. T, M.A. U, E. D, U. G, et al. Limberg flap procedure for surgical treatment of pilonidal sinus: Retrospective analyse of postoperative early complications. Eur Surg Res [Internet]. 2015;55(SUPPL. 1):115–6. Available from: http://ovidsp.ovid.com/ovidweb.cgi?T=JS&PAGE=reference&D=emed16&NEWS=N&AN=71958367

177. Akin M, Leventoglu S, Mentes BB, Bostanci H, Gokbayir H, Kilic K, et al. Comparison of the classic Limberg flap and modified Limberg flap in the treatment of pilonidal sinus disease: a retrospective analysis of 416 patients. Surg Today [Internet]. 2010;40(8):757–62. Available from: http://ovidsp.ovid.com/ovidweb.cgi?T=JS&PAGE=reference&D=med8&NEWS=N&AN=20676861

178. Hosseini M, Heidari A, Jafarnejad B. Comparison of Three Surgical Methods in Treatment of Patients with Pilonidal Sinus: Modified Excision and Repair/Wide Excision/Wide Excision and Flap in RASOUL, OMID and SADR Hospitals( 2004–2007). Indian J Surg [Internet]. 2013 Oct 18;75(5):395–400. Available from: http://search.ebscohost.com/login.aspx?direct=true&db=cin20&AN=91661570&site=ehost-live

179. Agcaoglu O, Dural AC, Ercetin C, Tezcaner T, KIRNAP M, Anuk T. Comparison of Cleft Lift and Limberg Flap Techniques for Pilonidal Sinus Surgery. East J Med [Internet]. 2019;24(3):320–4. Available from: https://www.journalagent.com/z4/download_fulltext.asp?pdir=ejm&plng=eng&un=EJM-40412

180. V.O. G, M. I, S. O, S. A. Abstracts of the 49th Congress of the European Society for Surgical Research, May 21-24, 2014, Budapest, Hungary. Eur Surg Res [Internet]. 2014;52(3–4):93–250. Available from: http://ovidsp.ovid.com/ovidweb.cgi?T=JS&PAGE=reference&D=emed15&NEWS=N&AN=71493360

181. Romaniszyn M, Swirta JS, Walega PJ. Long‐term results of endoscopic pilonidal sinus treatment vs Limberg flap for treatment of difficult cases of complicated pilonidal disease: a prospective, nonrandomized study. Color Dis [Internet]. 2020 Mar 6;22(3):319–24. Available from: http://onlinelibrary.wiley.com/journal/10.1111/(ISSN)1463-1318

182. S. R, S. Z, S.I. A, K. M, H. A. Rhomboid excision with limberg transposition flap in the management of sacrococcygeal pilonidal sinus - A reliable surgical technique. Pakistan J Med Heal Sci [Internet]. 2014;8(3):782–5. Available from: http://pjmhsonline.com/JulySep2014/rhomboid_excision_with_limberg_t.htm

183. D. G, M.L. G, O. A, Y. T, Z. O, M. K. Comparing phenol application after minimally surgical excision and flap repair for treatment of pilonidal sinus. Color Dis [Internet]. 2015;17(SUPPL. 2):53. Available from: http://ovidsp.ovid.com/ovidweb.cgi?T=JS&PAGE=reference&D=emed16&NEWS=N&AN=72057521

184. M.B. A-H, A.H. K, A.A. A, W. AB, W. F. Rhomboid flap - A primary cure for pilonidal sinus. Kuwait Med J [Internet]. 2010;42(4):282–5. Available from: http://www.kma.org.kw/KMJ/journals/December

185. Manterola C, Otzen T. Dufourmentel rhomboid flap in the radical treatment of extensive, complex or recurrent sacrococcygeal pilonidal disease: Case series with follow up. Surg Pract [Internet]. 2018 Feb;22(1):15–22. Available from: http://www.blackwell-synergy.com/loi/ash

186. J.K. S, B. D, S. G, U. S. Early experience with the Dufourmentel rhomboid flap procedure for the treatment of sacrococcygeal pilonidal disease. Eur Surg - Acta Chir Austriaca [Internet]. 2013;45(SUPPL. 2):S65. Available from: http://ovidsp.ovid.com/ovidweb.cgi?T=JS&PAGE=reference&D=emed14&NEWS=N&AN=71346479

187. Aithal SK, Rajan CS, Reddy N. Limberg Flap for Sacrococcygeal Pilonidal Sinus a Safe and Sound Procedure. Indian J Surg [Internet]. 2013 Aug 6;75(4):298–301. Available from: http://search.ebscohost.com/login.aspx?direct=true&db=cin20&AN=104204541&site=ehost-live

188. Lieto E, Castellano P, Pinto M, Zamboli A, Pignatelli C, Galizia G. Dufourmentel rhomboid flap in the radical treatment of primary and recurrent sacrococcygeal pilonidal disease. Dis Colon Rectum [Internet]. 2010;53(7):1061–8. Available from: http://ovidsp.ovid.com/ovidweb.cgi?T=JS&PAGE=reference&D=med8&NEWS=N&AN=20551760

189. Holmebakk T, Nesbakken A. Surgery for pilonidal disease. Scand J Surg [Internet]. 2005;94(1):43–6. Available from: http://ovidsp.ovid.com/ovidweb.cgi?T=JS&PAGE=reference&D=med6&NEWS=N&AN=15865116

190. A. A. Case series on modified Limberg flap technique in management of pilonidal disease. Int J Pharma Med Biol Sci [Internet]. 2013;2(2):51–6. Available from: http://www.ijpmbs.com/download.php?file=ijpmbsadmin/upload/ijpmbs_5159acfef2c72.pdf&iid=99

191. Washer JD, Smith DE, Carman ME, Blackhurst DW. Gluteal fascial advancement: an innovative, effective method for treating pilonidal disease. Am Surg [Internet]. 2010;76(2):154–6. Available from: http://ovidsp.ovid.com/ovidweb.cgi?T=JS&PAGE=reference&D=med8&NEWS=N&AN=20336891

192. Kapp T, Zadnikar M, Hahnloser D, Soll C, Hetzer FH. [New concept in the treatment of the pilonidal sinus]. Neues Ther fur den Sinus pilonidalis [Internet]. 2007;96(31–32):1171–6. Available from: http://ovidsp.ovid.com/ovidweb.cgi?T=JS&PAGE=reference&D=med6&NEWS=N&AN=17726856

193. Ersoy OF, Karaca S, Kayaoglu HA, Ozkan N, Celik A, Ozum T. Comparison of different surgical options in the treatment of pilonidal disease: retrospective analysis of 175 patients. Kaohsiung J Med Sci [Internet]. 2007;23(2):67–70. Available from: http://ovidsp.ovid.com/ovidweb.cgi?T=JS&PAGE=reference&D=med6&NEWS=N&AN=17339168

194. Singh R, Pavithran NM. Adipo-fascio-cutaneous flaps in the treatment of pilonidal sinus: experience with 50 cases. Asian J Surg [Internet]. 2005;28(3):198–201. Available from: http://ovidsp.ovid.com/ovidweb.cgi?T=JS&PAGE=reference&D=med6&NEWS=N&AN=16024316

195. Cihan A, Mentes BB, Tatlicioglu E, Ozmen S, Leventoglu S, Ucan BH. Modified Limberg flap reconstruction compares favourably with primary repair for pilonidal sinus surgery. ANZ J Surg [Internet]. 2004;74(4):238–42. Available from: http://ovidsp.ovid.com/ovidweb.cgi?T=JS&PAGE=reference&D=med5&NEWS=N&AN=15043735

196. Hegele A, Strombach FJ, Schonbach F. [Reconstructive surgical therapy of infected pilonidal sinus]. Plast Ther des infizierten Sinus pilonidalis [Internet]. 2003;74(8):749–52. Available from: http://ovidsp.ovid.com/ovidweb.cgi?T=JS&PAGE=reference&D=med5&NEWS=N&AN=12928797

197. Aydede H, Erhan Y, Sakarya A, Kumkumoglu Y. Comparison of three methods in surgical treatment of pilonidal disease. ANZ J Surg [Internet]. 2001;71(6):362–4. Available from: http://ovidsp.ovid.com/ovidweb.cgi?T=JS&PAGE=reference&D=med4&NEWS=N&AN=11409022

198. Sharma PP. Multiple Z-plasty in pilonidal sinus--a new technique under local anesthesia. World J Surg [Internet]. 2006;30(12):2261–5. Available from: http://ovidsp.ovid.com/ovidweb.cgi?T=JS&PAGE=reference&D=med6&NEWS=N&AN=17120183

199. Quinodoz PD, Chilcott M, Grolleau JL, Chavoin JP, Costagliola M. Surgical treatment of sacrococcygeal pilonidal sinus disease by excision and skin flaps: the Toulouse experience. Eur J Surg [Internet]. 1999;165(11):1061–5. Available from: http://ovidsp.ovid.com/ovidweb.cgi?T=JS&PAGE=reference&D=med4&NEWS=N&AN=10595611

200. Lamke LO, Larsson J, Nylen B. Treatment of pilonidal sinus by radical excision and reconstruction by rotation flap surgery of Z-plasty technique. Scand J Plast Reconstr Surg [Internet]. 1979;13(2):351–3. Available from: http://ovidsp.ovid.com/ovidweb.cgi?T=JS&PAGE=reference&D=med1&NEWS=N&AN=397614

201. Zagory JA, Golden J, Holoyda K, Demeter N, Nguyen NX. Excision and Primary Closure May Be the Better Option in the Surgical Management of Pilonidal Disease in the Pediatric Population. Am Surg [Internet]. 2016;82(10):964–7. Available from: http://ovidsp.ovid.com/ovidweb.cgi?T=JS&PAGE=reference&D=med13&NEWS=N&AN=27779984

202. Omer Y, Hayrettin D, Murat C, Mustafa Y, Evren D. Comparison of modified limberg flap and modified elliptical rotation flap for pilonidal sinus surgery: a retrospective cohort study. Int J Surg [Internet]. 2015;16(Pt A):74–7. Available from: http://ovidsp.ovid.com/ovidweb.cgi?T=JS&PAGE=reference&D=med12&NEWS=N&AN=25758346

203. Yazar M, Kurt Yazar S, Celet Ozden B, Guven E, Basaran K, Alyanak A, et al. Cosmetic closure of pilonidal sinus defects with bilateral transpositional adipofascial flaps. J Plast Surg Hand Surg [Internet]. 2013;47(4):292–6. Available from: http://ovidsp.ovid.com/ovidweb.cgi?T=JS&PAGE=reference&D=med10&NEWS=N&AN=23731131

204. Ekci B, Gokce O. A new flap technique to treat pilonidal sinus. Tech Coloproctol [Internet]. 2009;13(3):205–9. Available from: http://ovidsp.ovid.com/ovidweb.cgi?T=JS&PAGE=reference&D=med7&NEWS=N&AN=19597938

205. Lasheen AE, Saad K, Raslan M. Crossed triangular flaps technique for surgical treatment of chronic pilonidal sinus disease. Arch Surg [Internet]. 2008;143(5):503–5. Available from: http://ovidsp.ovid.com/ovidweb.cgi?T=JS&PAGE=reference&D=med7&NEWS=N&AN=18490562

206. Katsoulis IE, Hibberts F, Carapeti EA. Outcome of treatment of primary and recurrent pilonidal sinuses with the Limberg flap. Surgeon [Internet]. 2006;4(1):7–62. Available from: http://ovidsp.ovid.com/ovidweb.cgi?T=JS&PAGE=reference&D=med6&NEWS=N&AN=16459493

207. Ay A, Aytekin O, Aytekin A. Interdigitating fasciocutaneous gluteal V-Y advancement flaps for reconstruction of sacral defects. Ann Plast Surg [Internet]. 2003;50(6):636–8. Available from: http://ovidsp.ovid.com/ovidweb.cgi?T=JS&PAGE=reference&D=med5&NEWS=N&AN=12783019

208. Demiryas S, Donmez T. Could Early Postoperative Complications be Considered as Risk Factor for Recurrence after Pilonidal Sinus Surgery?. Chirurgia (Bucur) [Internet]. 2019;114(4):475–86. Available from: http://ovidsp.ovid.com/ovidweb.cgi?T=JS&PAGE=reference&D=med16&NEWS=N&AN=31511134

209. Moosavi SR, Kharazm P, Vaghardoost R. Surgical treatment of pilonidal sinus with a fasciocutaneous rotation flap based on an inferior pedicle. Scand J Plast Reconstr Surg hand Surg [Internet]. 2006;40(5):281–3. Available from: http://ovidsp.ovid.com/ovidweb.cgi?T=JS&PAGE=reference&D=med6&NEWS=N&AN=17065117

210. Kartal A, Aydin HO, Oduncu M, Ferhatoglu MF, Kivilcim T, Filiz AI. Comparison of Three Surgical Techniques in Pilonidal Sinus Surgery. Prague Med Rep [Internet]. 2018;119(4):148–55. Available from: http://ovidsp.ovid.com/ovidweb.cgi?T=JS&PAGE=reference&D=med15&NEWS=N&AN=30779699

211. Arpaci E, Altun S, Orhan E, Eyuboglu A, Ertas NM. A New Oval Advancement Flap Design for Reconstruction of Pilonidal Sinus Defect. World J Surg [Internet]. 2018;42(11):3568–74. Available from: http://ovidsp.ovid.com/ovidweb.cgi?T=JS&PAGE=reference&D=med15&NEWS=N&AN=29713735

212. Koca YS, Yildiz I, Ugur M, Barut I. The V-Y flap technique in complicated and recurrent pilonidal sinus disease. Ann Ital Chir [Internet]. 2018;89:66–9. Available from: http://ovidsp.ovid.com/ovidweb.cgi?T=JS&PAGE=reference&D=med15&NEWS=N&AN=29629896

213. Mackowski A, Levitt M. Outcomes of the house advancement flap for pilonidal sinus. ANZ J Surg [Internet]. 2017;87(9):692–4. Available from: http://ovidsp.ovid.com/ovidweb.cgi?T=JS&PAGE=reference&D=med14&NEWS=N&AN=25787062

214. Stosic M, Stojanovic I. HIDRADENITIS SUPPURATIVA: A CASE SERIES OF EIGHT PATIENTS. Med Pregl [Internet]. 2016;69(1–2):48–52. Available from: http://ovidsp.ovid.com/ovidweb.cgi?T=JS&PAGE=reference&D=med13&NEWS=N&AN=27498534

215. Powell BC, Webb CB, Ewing JA, Smith DE. Gluteal Fascial Advancement for Pilonidal Cyst Disease: A 10-year Review. Am Surg [Internet]. 2016;82(7):622–5. Available from: http://ovidsp.ovid.com/ovidweb.cgi?T=JS&PAGE=reference&D=med13&NEWS=N&AN=27457861

216. Braungart S, Powis M, Sutcliffe JR, Sugarman ID. Improving outcomes in pilonidal sinus disease. J Pediatr Surg [Internet]. 2016;51(2):282–4. Available from: http://ovidsp.ovid.com/ovidweb.cgi?T=JS&PAGE=reference&D=med13&NEWS=N&AN=26690708

217. Sekmenli T, Ciftci I. Surgical therapy for pilonidal sinus in adolescents: a retrospective study. Med Glas (Zenica) [Internet]. 2015;12(1):57–60. Available from: http://ovidsp.ovid.com/ovidweb.cgi?T=JS&PAGE=reference&D=med12&NEWS=N&AN=25669338

218. Ozdemir H, Unal Ozdemir Z, Tayfun Sahiner I, Senol M. Whole natal cleft excision and flap : an alternative surgical method in extensive sacrococcygeal pilonidal sinus disease. Acta Chir Belg [Internet]. 2014;114(4):266–70. Available from: http://ovidsp.ovid.com/ovidweb.cgi?T=JS&PAGE=reference&D=med11&NEWS=N&AN=26021423

219. Yildiz T, Ilce Z, Kucuk A. Modified Limberg flap technique in the treatment of pilonidal sinus disease in teenagers. J Pediatr Surg [Internet]. 2014;49(11):1610–3. Available from: http://ovidsp.ovid.com/ovidweb.cgi?T=JS&PAGE=reference&D=med11&NEWS=N&AN=25475804

220. Tekin A. A simple modification with the Limberg flap for chronic pilonidal disease. Surgery [Internet]. 2005;138(5):951–3. Available from: http://ovidsp.ovid.com/ovidweb.cgi?T=JS&PAGE=reference&D=med6&NEWS=N&AN=16291398

221. Pazdirek F, Kouda M, Jech Z, Frajer L, J JH. [Pilonidal sinus - diagnosis at the intersection of general and plastic surgery]. Pilonidalni sinus - diagnoza na pomezi Vseob a Plast Chir [Internet]. 2014;93(11):545–8. Available from: http://ovidsp.ovid.com/ovidweb.cgi?T=JS&PAGE=reference&D=med11&NEWS=N&AN=25418942

222. Jandik J. [Sinus pilonidalis - possibilities of surgical treatment]. Sinus pilonidalis - moznosti Chir lecby [Internet]. 2014;93(10):496–501. Available from: http://ovidsp.ovid.com/ovidweb.cgi?T=JS&PAGE=reference&D=med11&NEWS=N&AN=25340864

223. Orhalmi J, Sotona O, Dusek T, Ferko A. [Pilonidal sinus - possibilities surgical treatment]. Pilonidalni sinus - moznosti operacniho Res [Internet]. 2014;93(10):491–5. Available from: http://ovidsp.ovid.com/ovidweb.cgi?T=JS&PAGE=reference&D=med11&NEWS=N&AN=25340863

224. Gordon P, Grant L, Irwin T. Recurrent pilonidal sepsis. Ulster Med J [Internet]. 2014;83(1):10–2. Available from: http://ovidsp.ovid.com/ovidweb.cgi?T=JS&PAGE=reference&D=med11&NEWS=N&AN=24757262

225. Altintoprak F, Gundogdu K, Ergonenc T, Dikicier E, Cakmak G, Celebi F. Retrospective review of pilonidal sinus patients with early discharge after Limberg flap procedure. Int Surg [Internet]. 2014;99(1):28–34. Available from: http://ovidsp.ovid.com/ovidweb.cgi?T=JS&PAGE=reference&D=med11&NEWS=N&AN=24444265

226. Karaca T, Yoldas O, Bilgin BC, Ozer S, Yoldas S, Karaca NG. Comparison of short-term results of modified Karydakis flap and modified Limberg flap for pilonidal sinus surgery. Int J Surg [Internet]. 2012;10(10):601–6. Available from: http://ovidsp.ovid.com/ovidweb.cgi?T=JS&PAGE=reference&D=med9&NEWS=N&AN=23092625

227. Kaya B, Eris C, Atalay S, Bat O, Bulut NE, Mantoglu B, et al. Modified Limberg transposition flap in the treatment of pilonidal sinus disease. Tech Coloproctol [Internet]. 2012;16(1):55–9. Available from: http://ovidsp.ovid.com/ovidweb.cgi?T=JS&PAGE=reference&D=med9&NEWS=N&AN=22170253

228. Fike FB, Mortellaro VE, Juang D, Ostlie DJ, St Peter SD. Experience with pilonidal disease in children. J Surg Res [Internet]. 2011;170(1):165–8. Available from: http://ovidsp.ovid.com/ovidweb.cgi?T=JS&PAGE=reference&D=med8&NEWS=N&AN=21470629

229. Yamout SZ, Caty MG, Lee Y-H, Lau ST, Escobar MA, Glick PL. Early experience with the use of rhomboid excision and Limberg flap in 16 adolescents with pilonidal disease. J Pediatr Surg [Internet]. 2009;44(8):1586–90. Available from: http://ovidsp.ovid.com/ovidweb.cgi?T=JS&PAGE=reference&D=med7&NEWS=N&AN=19635310

230. Krand O, Yalt T, Berber I, Kara VM, Tellioglu G. Management of pilonidal sinus disease with oblique excision and bilateral gluteus maximus fascia advancing flap: result of 278 patients. Dis Colon Rectum [Internet]. 2009;52(6):1172–7. Available from: http://ovidsp.ovid.com/ovidweb.cgi?T=JS&PAGE=reference&D=med7&NEWS=N&AN=19581864

231. Daphan C, Tekelioglu MH, Sayilgan C. Limberg flap repair for pilonidal sinus disease. Dis Colon Rectum [Internet]. 2004;47(2):233–7. Available from: http://ovidsp.ovid.com/ovidweb.cgi?T=JS&PAGE=reference&D=med5&NEWS=N&AN=15043295

232. El-Khatib HA, Al-Basti HB. A perforator-based bilobed fasciocutaneous flap: an additional tool for primary reconstruction following wide excision of sacrococcygeal pilonidal disease. J Plast Reconstr Aesthet Surg [Internet]. 2009;62(4):494–8. Available from: http://ovidsp.ovid.com/ovidweb.cgi?T=JS&PAGE=reference&D=med7&NEWS=N&AN=18249047

233. Unalp HR, Derici H, Kamer E, Nazli O, Onal MA. Lower recurrence rate for Limberg vs. V-Y flap for pilonidal sinus. Dis Colon Rectum [Internet]. 2007;50(9):1436–44. Available from: http://ovidsp.ovid.com/ovidweb.cgi?T=JS&PAGE=reference&D=med6&NEWS=N&AN=17661144

234. Misiakos EP, Troupis T, Hatzikokolis S, Macheras A, Liakakos T, Patapis P, et al. Limberg flap reconstruction for the treatment of pilonidal sinus disease. Chirurgia (Bucur) [Internet]. 2006;101(5):513–7. Available from: http://ovidsp.ovid.com/ovidweb.cgi?T=JS&PAGE=reference&D=med6&NEWS=N&AN=17278644

235. Topgul K, Ozdemir E, Kilic K, Gokbayir H, Ferahkose Z. Long-term results of limberg flap procedure for treatment of pilonidal sinus: a report of 200 cases. Dis Colon Rectum [Internet]. 2003;46(11):1545–8. Available from: http://ovidsp.ovid.com/ovidweb.cgi?T=JS&PAGE=reference&D=med5&NEWS=N&AN=14605577

236. Cubukcu A, Gonullu NN, Paksoy M, Alponat A, Kuru M, Ozbay O. The role of obesity on the recurrence of pilonidal sinus disease in patients, who were treated by excision and Limberg flap transposition. Int J Colorectal Dis [Internet]. 2000;15(3):173–5. Available from: http://ovidsp.ovid.com/ovidweb.cgi?T=JS&PAGE=reference&D=med4&NEWS=N&AN=10954190

237. Abo-Ryia MH, Abd-Allah HS, Al-Shareef MM, Abdulrazek MM. Fascio-Adipo-Cutaneous Lateral Advancement Flap for Treatment of Pilonidal Sinus: A Modification of the Karydakis Operation-Cohort Study. World J Surg [Internet]. 2018;42(6):1721–6. Available from: http://ovidsp.ovid.com/ovidweb.cgi?T=JS&PAGE=reference&D=med15&NEWS=N&AN=29270650

238. Hamnett K, Nagarajan M, Iqbal A. Inferiorly based lotus petal flap & laser therapy in difficult pilonidal sinus management. J Plast Reconstr Aesthet Surg [Internet]. 2018;71(11):1631–6. Available from: http://ovidsp.ovid.com/ovidweb.cgi?T=JS&PAGE=reference&D=med15&NEWS=N&AN=30104141

239. Karakas BR, Aslaner A, Gunduz UR, Calis H, Ongen AN, Oner OZ, et al. Is the lateralization distance important in terms in patients undergoing the modified Limberg flap procedure for treatment of pilonidal sinus?. Tech Coloproctol [Internet]. 2015;19(5):309–16. Available from: http://ovidsp.ovid.com/ovidweb.cgi?T=JS&PAGE=reference&D=med12&NEWS=N&AN=25445835

240. Spychala A, Murawa D. The Limberg flap procedure in the treatment of pilonidal cyst disease of the sacrum - initial report. Pol Przegl Chir [Internet]. 2014;86(6):257–62. Available from: http://ovidsp.ovid.com/ovidweb.cgi?T=JS&PAGE=reference&D=med11&NEWS=N&AN=25205695

241. Karakas BR. Comparison of Z-plasty, limberg flap, and asymmetric modified Limberg flap techniques for the pilonidal sinus treatment: review of literature. Acta Chir Iugosl [Internet]. 2013;60(3):31–7. Available from: http://ovidsp.ovid.com/ovidweb.cgi?T=JS&PAGE=reference&D=med10&NEWS=N&AN=24669578

242. Ekici U, Kanlioz M, Ferhatoglu MF, Kartal A. A comparative analysis of four different surgical methods for treatment of sacrococcygeal pilonidal sinus. Asian J Surg [Internet]. 2019;42(10):907–13. Available from: http://ovidsp.ovid.com/ovidweb.cgi?T=JS&PAGE=reference&D=medl&NEWS=N&AN=30685149

243. Kim YH, Naidu S, Kim CY, Lee KH, Kim JT. A perforator solution for excisional defects of pilonidal sinus. J Plast Reconstr Aesthet Surg [Internet]. 2011;64(1):138–40. Available from: http://ovidsp.ovid.com/ovidweb.cgi?T=JS&PAGE=reference&D=med8&NEWS=N&AN=20634161

244. El-Tawil S, Carapeti E. Use of a double rhomboid transposition flap in the treatment of extensive complex pilonidal sinus disease. Colorectal Dis [Internet]. 2009;11(3):313–7. Available from: http://ovidsp.ovid.com/ovidweb.cgi?T=JS&PAGE=reference&D=med7&NEWS=N&AN=18513189

245. Mahdy T. Surgical treatment of the pilonidal disease: primary closure or flap reconstruction after excision. Dis Colon Rectum [Internet]. 2008;51(12):1816–22. Available from: http://ovidsp.ovid.com/ovidweb.cgi?T=JS&PAGE=reference&D=med7&NEWS=N&AN=18937009

246. Khubezov DA, Lukanin R V, Ogoreltsev AY, Puchkov DK, Serebryansky P V, Yudina EA, et al. [Selection of the method for surgical treatment of pilonidal disease without abscess formation]. Vyb Metod khirurgicheskogo lecheniia pilonidal’noi Bolezn bez abstsedirovaniia [Internet]. 2019;(8. Vyp. 2):24–31. Available from: http://ovidsp.ovid.com/ovidweb.cgi?T=JS&PAGE=reference&D=med16&NEWS=N&AN=31502590

247. Ishii N, Shimizu Y, Oji T, Kishi K. Modified Dufourmentel flap with superior pedicle: a useful technique for sacrococcygeal pilonidal sinus. J Plast Surg Hand Surg [Internet]. 2017;51(6):453–7. Available from: http://ovidsp.ovid.com/ovidweb.cgi?T=JS&PAGE=reference&D=med14&NEWS=N&AN=28417653

248. Sebastian M, Sroczynski M, Rudnicki J. The Dufourmentel modification of the limberg flap: Does it fit all?. Adv Clin Exp Med [Internet]. 2017;26(1):63–7. Available from: http://ovidsp.ovid.com/ovidweb.cgi?T=JS&PAGE=reference&D=med14&NEWS=N&AN=28397434

249. Mutaf M, Temel M, Koc MN. A New Surgical Technique for Closure of Pilonidal Sinus Defects: Triangular Closure Technique. Med Sci Monit [Internet]. 2017;23:1033–42. Available from: http://ovidsp.ovid.com/ovidweb.cgi?T=JS&PAGE=reference&D=med14&NEWS=N&AN=28238003

250. Gupta A, Anand S, Mehrotra S, Khidtta K. Learning Curve for Pilonidal Sinus Surgery: The Best Option for Budding Surgeons. World J Surg [Internet]. 2017;41(2):615–9. Available from: http://ovidsp.ovid.com/ovidweb.cgi?T=JS&PAGE=reference&D=med14&NEWS=N&AN=27464913

251. Ardelt M, Dittmar Y, Scheuerlein H, Fahrner R, Rauchfus F, Settmacher U. [Sequential intervention with primary excision and Limberg plastic surgery procedure for treatment of sacrococcygeal pilonidal sinus : Results of a pilot study]. Zweizeitiges Verfahren mit primarer Exzision und Limberg-Plastik zur Ther eines sakrokokzygealen Pilonidalsinus Ergebnisse einer Pilot [Internet]. 2015;86(8):771–5. Available from: http://ovidsp.ovid.com/ovidweb.cgi?T=JS&PAGE=reference&D=med12&NEWS=N&AN=25616747

252. Yuksel BC, Berkem H, Ozel H, Hengirmen S. A new surgical method of pilonidal sinus treatment: a bilaterally paralel elliptic fascio-cutaneous advancement flap technique. Bratisl Lek Listy [Internet]. 2012;113(12):728–31. Available from: http://ovidsp.ovid.com/ovidweb.cgi?T=JS&PAGE=reference&D=med9&NEWS=N&AN=23173633

253. Erkent M, Sahiner IT, Bala M, Kendirci M, Yildirim MB, Topcu R, et al. Comparison of Primary Midline Closure, Limberg Flap, and Karydakis Flap Techniques in Pilonidal Sinus Surgery. Med Sci Monit [Internet]. 2018;24:8959–63. Available from: http://ovidsp.ovid.com/ovidweb.cgi?T=JS&PAGE=reference&D=med15&NEWS=N&AN=30531689

254. Onder A, Girgin S, Kapan M, Toker M, Arikanoglu Z, Palanci Y, et al. Pilonidal sinus disease: risk factors for postoperative complications and recurrence. Int Surg [Internet]. 2012;97(3):224–9. Available from: http://ovidsp.ovid.com/ovidweb.cgi?T=JS&PAGE=reference&D=med9&NEWS=N&AN=23113850

255. Schrogendorfer KF, Haslik W, Aszmann OC, Vierhapper M, Frey M, Lumenta DB. Prospective evaluation of a single-sided innervated gluteal artery perforator flap for reconstruction for extensive and recurrent pilonidal sinus disease: functional, aesthetic, and patient-reported long-term outcomes. World J Surg [Internet]. 2012;36(9):2230–6. Available from: http://ovidsp.ovid.com/ovidweb.cgi?T=JS&PAGE=reference&D=med9&NEWS=N&AN=22552500

256. Venus MR, Titley OG. Outcomes in the repair of pilonidal sinus disease excision wounds using a parasacral perforator flap. Ann R Coll Surg Engl [Internet]. 2012;94(1):12–6. Available from: http://ovidsp.ovid.com/ovidweb.cgi?T=JS&PAGE=reference&D=med9&NEWS=N&AN=22524909

257. Muller K, Marti L, Tarantino I, Jayne DG, Wolff K, Hetzer FH. Prospective analysis of cosmesis, morbidity, and patient satisfaction following Limberg flap for the treatment of sacrococcygeal pilonidal sinus. Dis Colon Rectum [Internet]. 2011;54(4):487–94. Available from: http://ovidsp.ovid.com/ovidweb.cgi?T=JS&PAGE=reference&D=med8&NEWS=N&AN=21383571

258. Polat C, Gungor B, Karagul S, Buyukakincak S, Topgul K, Erzurumlu K. Is oval flap reconstruction a good modification for treating pilonidal sinuses?. Am J Surg [Internet]. 2011;201(2):192–6. Available from: http://ovidsp.ovid.com/ovidweb.cgi?T=JS&PAGE=reference&D=med8&NEWS=N&AN=20538254

259. Madbouly KM. Day-case Limberg flap for recurrent pilonidal sinus: does obesity complicate the issue?. Am Surg [Internet]. 2010;76(9):995–9. Available from: http://ovidsp.ovid.com/ovidweb.cgi?T=JS&PAGE=reference&D=med8&NEWS=N&AN=20836350

260. Kicka M, Toporcer T, Radonak J. [Pilonidal sinus--a classical plastic procedure according to Limberg (Limberg flap procedure) or its modified version?]. Pilonidalny sinus - klasicka Plast Pod Limberga alebo Modif verzia? [Internet]. 2011;90(8):482–7. Available from: http://ovidsp.ovid.com/ovidweb.cgi?T=JS&PAGE=reference&D=med8&NEWS=N&AN=22272478

261. Dizen H, Yoldas O, Yildiz M, Cilekar M, Dilektasli E. Modified elliptical rotation flap for sacrococcygeal pilonidal sinus disease. ANZ J Surg [Internet]. 2014;84(10):769–71. Available from: http://ovidsp.ovid.com/ovidweb.cgi?T=JS&PAGE=reference&D=med11&NEWS=N&AN=25143150

262. Demiryilmaz I, Yilmaz I, Peker K, Celebi F, Cimen O, Isik A, et al. Application of fasciocutaneous V-Y advancement flap in primary and recurrent sacrococcygeal pilonidal sinus disease. Med Sci Monit [Internet]. 2014;20:1263–6. Available from: http://ovidsp.ovid.com/ovidweb.cgi?T=JS&PAGE=reference&D=med11&NEWS=N&AN=25042095

263. Yildar M, Cavdar F, Yildiz MK. The evaluation of a modified Dufourmentel flap after S-type excision for pilonidal sinus disease. ScientificWorldJournal [Internet]. 2013;2013:459147. Available from: http://ovidsp.ovid.com/ovidweb.cgi?T=JS&PAGE=reference&D=med10&NEWS=N&AN=23853537

264. Abdelnaby M, Emile SH, El-Said M, AbdelMawla A, Elgendy H, Sakr A, et al. Rotational gluteal flap versus modified Limberg flap in treatment of sacrococcygeal pilonidal disease. J Surg Res [Internet]. 2018;223:174–82. Available from: http://ovidsp.ovid.com/ovidweb.cgi?T=JS&PAGE=reference&D=med15&NEWS=N&AN=29433871

265. Elalfy K, Emile S, Lotfy A, Youssef M, Elfeki H. Bilateral gluteal advancement flap for treatment of recurrent sacrococcygeal pilonidal disease: A prospective cohort study. Int J Surg [Internet]. 2016;29:1–8. Available from: http://ovidsp.ovid.com/ovidweb.cgi?T=JS&PAGE=reference&D=med13&NEWS=N&AN=26975846

266. Chaput B, Herlin C, Jacques J, Berthier C, Meresse T, Bekara F, et al. Management of Pilonidal Sinus Disease with the Aesthetically Shaped Parasacral Perforator Flap: Multicenter Evaluation of 228 Patients. Plast Reconstr Surg [Internet]. 2019;144(4):971–80. Available from: http://ovidsp.ovid.com/ovidweb.cgi?T=JS&PAGE=reference&D=medl&NEWS=N&AN=31568314

267. Darwish AMA, Hassanin A. Reconstruction following excision of sacrococcygeal pilonidal sinus with a perforator-based fasciocutaneous Limberg flap. J Plast Reconstr Aesthet Surg [Internet]. 2010;63(7):1176–80. Available from: http://ovidsp.ovid.com/ovidweb.cgi?T=JS&PAGE=reference&D=med8&NEWS=N&AN=19617016

268. Acarturk TO, Parsak CK, Sakman G, Demircan O. Superior gluteal artery perforator flap in the reconstruction of pilonidal sinus. J Plast Reconstr Aesthet Surg [Internet]. 2010;63(1):133–9. Available from: http://ovidsp.ovid.com/ovidweb.cgi?T=JS&PAGE=reference&D=med8&NEWS=N&AN=19010110

269. Aslam MN, Shoaib S, Choudhry AM. Use of Limberg flap for pilonidal sinus--a viable option. J Ayub Med Coll Abbottabad [Internet]. 2009;21(4):31–3. Available from: http://ovidsp.ovid.com/ovidweb.cgi?T=JS&PAGE=reference&D=med7&NEWS=N&AN=21067019

270. Yeo MS-W, Shim TW-H, Cheong WK, Leong APK, Lee SJ. Simultaneous laser depilation and perforator-based fasciocutaneous limberg flap for pilonidal sinus reconstruction. J Plast Reconstr Aesthet Surg [Internet]. 2010;63(11):e798-800. Available from: http://ovidsp.ovid.com/ovidweb.cgi?T=JS&PAGE=reference&D=med8&NEWS=N&AN=20708989

271. Eryilmaz R, Okan I, Coskun A, Bas G, Sahin M. Surgical treatment of complicated pilonidal sinus with a fasciocutaneous V-Y advancement flap. Dis Colon Rectum [Internet]. 2009;52(12):2036–40. Available from: http://ovidsp.ovid.com/ovidweb.cgi?T=JS&PAGE=reference&D=med7&NEWS=N&AN=19934927

272. el-Khadrawy O, Hashish M, Ismail K, Shalaby H. Outcome of the rhomboid flap for recurrent pilonidal disease. World J Surg [Internet]. 2009;33(5):1064–8. Available from: http://ovidsp.ovid.com/ovidweb.cgi?T=JS&PAGE=reference&D=med7&NEWS=N&AN=19198934

273. Basterzi Y, Canbaz H, Aksoy A, Sar A, Turkmenoglu MO, Caglkulekci M. Reconstruction of extensive pilonidal sinus defects with the use of S-GAP flaps. Ann Plast Surg [Internet]. 2008;61(2):197–200. Available from: http://ovidsp.ovid.com/ovidweb.cgi?T=JS&PAGE=reference&D=med7&NEWS=N&AN=18650614

274. Pomazkin VI, Mansurov I V. [Choice of operation for treatment of patients with pilonidal sinus]. Vestn Khir Im I I Grek [Internet]. 2008;167(1):85–7. Available from: http://ovidsp.ovid.com/ovidweb.cgi?T=JS&PAGE=reference&D=med7&NEWS=N&AN=18411678

275. Hardy EJO, Herrod PJ, Doleman B, Phillips HG, Ranat R, Lund JN. Surgical interventions for the treatment of sacrococcygeal pilonidal sinus disease in children: A systematic review and meta-analysis. J Pediatr Surg [Internet]. 2019;54(11):2222–33. Available from: http://ovidsp.ovid.com/ovidweb.cgi?T=JS&PAGE=reference&D=medl&NEWS=N&AN=30940347

276. Milone M, Velotti N, Manigrasso M, Anoldo P, Milone F, De Palma GD. Long-term follow-up for pilonidal sinus surgery: A review of literature with metanalysis. Surgeon [Internet]. 2018;16(5):315–20. Available from: http://ovidsp.ovid.com/ovidweb.cgi?T=JS&PAGE=reference&D=med15&NEWS=N&AN=29699781

277. Sewefy AM, Hassanen A, Atyia AM, Saleh SK. Karydakis Flap With Compressing Tie-over Interrupted Sutures Without Drain versus Standard Karydakis for Treatment of Sacrococcygeal Pilonidal Sinus Disease. Dis Colon Rectum [Internet]. 2017;60(5):514–20. Available from: http://ovidsp.ovid.com/ovidweb.cgi?T=JS&PAGE=reference&D=med14&NEWS=N&AN=28383451

278. Sevinc B, Karahan O, Okus A, Ay S, Aksoy N, Simsek G. Randomized prospective comparison of midline and off-midline closure techniques in pilonidal sinus surgery. Surgery [Internet]. 2016;159(3):749–54. Available from: http://ovidsp.ovid.com/ovidweb.cgi?T=JS&PAGE=reference&D=med13&NEWS=N&AN=26531235

279. Keshvari A, Keramati MR, Fazeli MS, Kazemeini A, Meysamie A, Nouritaromlou MK. Karydakis flap versus excision-only technique in pilonidal disease. J Surg Res [Internet]. 2015;198(1):260–6. Available from: http://ovidsp.ovid.com/ovidweb.cgi?T=JS&PAGE=reference&D=med12&NEWS=N&AN=26094093

280. Tokac M, Dumlu EG, Aydin MS, Yalcin A, Kilic M. Comparison of modified Limberg flap and Karydakis flap operations in pilonidal sinus surgery: prospective randomized study. Int Surg [Internet]. 2015;100(5):870–7. Available from: http://ovidsp.ovid.com/ovidweb.cgi?T=JS&PAGE=reference&D=med12&NEWS=N&AN=26011208

281. Sozen S, Emir S, Guzel K, Ozdemir CS. Are postoperative drains necessary with the Karydakis flap for treatment of pilonidal sinus? (Can fibrin glue be replaced to drains?) A prospective randomized trial. Ir J Med Sci [Internet]. 2011;180(2):479–82. Available from: http://ovidsp.ovid.com/ovidweb.cgi?T=JS&PAGE=reference&D=med8&NEWS=N&AN=20721696

282. Nordon IM, Senapati A, Cripps NPJ. A prospective randomized controlled trial of simple Bascom’s technique versus Bascom’s cleft closure for the treatment of chronic pilonidal disease. Am J Surg [Internet]. 2009;197(2):189–92. Available from: http://ovidsp.ovid.com/ovidweb.cgi?T=JS&PAGE=reference&D=med7&NEWS=N&AN=18639221

283. Gurer A, Gomceli I, Ozdogan M, Ozlem N, Sozen S, Aydin R. Is routine cavity drainage necessary in Karydakis flap operation? A prospective, randomized trial. Dis Colon Rectum [Internet]. 2005;48(9):1797–9. Available from: http://ovidsp.ovid.com/ovidweb.cgi?T=JS&PAGE=reference&D=med6&NEWS=N&AN=15981071

284. Popeskou SG, Pravini B, Panteleimonitis S, Vajana AFDT, Vanoni A, Schmalzbauer M, et al. Conservative Sinusectomy vs. excision and primary off-midline closure for pilonidal disease: a randomized controlled trial. Int J Colorectal Dis [Internet]. 2020 Jul 6;35(7):1193–9. Available from: http://link.springer.de/link/service/journals/00384/index.htm

285. Kartal A, Yalçın M, Oter V, Ferhatoğlu MF, Uzunköy A. Asymmetric sinus excision and primary closure with additional skin excision technique. Effect of reduction of dead-space with Karydakis modification. Ann Ital Chir [Internet]. 2019;90:574–9. Available from: http://ovidsp.ovid.com/ovidweb.cgi?T=JS&PAGE=reference&D=medl&NEWS=N&AN=31354153

286. Milone M, Velotti N, Manigrasso M, Vertaldi S, Di Lauro K, De Simone G, et al. Long-term results of a randomized clinical trial comparing endoscopic versus conventional treatment of pilonidal sinus. Int J Surg [Internet]. 2020 Feb;74:81–5. Available from: http://ovidsp.ovid.com/ovidweb.cgi?T=JS&PAGE=reference&D=medl&NEWS=N&AN=31926328

287. A. FDTV, B. P, A. C, M. S, A. P, R. R. Fistulectomy versus excision and paramedian primary closure for pilonidal sinus: A randomized controlled trial. Color Dis [Internet]. 2017;19(Supplement 2):32. Available from: http://ovidsp.ovid.com/ovidweb.cgi?T=JS&PAGE=reference&D=emed18&NEWS=N&AN=618608525

288. T. T, C. D, C. E, M. K. Comparison of Limberg flap and cleft lift procedure for sacrococcygeal pilonidal sinus surgery: A case control study. Eur Surg - Acta Chir Austriaca [Internet]. 2012;44(SUPPL. 247):45. Available from: http://ovidsp.ovid.com/ovidweb.cgi?T=JS&PAGE=reference&D=emed13&NEWS=N&AN=71644722

289. M. T, F. Y, A.E. S, A.B. O, M. O, D. D. Comparison of modified primary repair, marsupialisation and Limberg flap techniques for pilonidal disease: A prospective randomized study. Eur Surg - Acta Chir Austriaca [Internet]. 2012;44(SUPPL. 247):64. Available from: http://ovidsp.ovid.com/ovidweb.cgi?T=JS&PAGE=reference&D=emed13&NEWS=N&AN=71644780

290. A.Y.L. N, D. N. Patient experience of negative pressure wound therapy (NPWT) after modified Bascom’s procedure. Br J Surg [Internet]. 2019;106(Supplement 5):110. Available from: http://ovidsp.ovid.com/ovidweb.cgi?T=JS&PAGE=reference&D=emexb&NEWS=N&AN=631722026

291. O. O, N. V, J. M. Poster Abstracts. Color Dis [Internet]. 2017 Sep;19(Supplement 2):35–139. Available from: http://ovidsp.ovid.com/ovidweb.cgi?T=JS&PAGE=reference&D=emed18&NEWS=N&AN=618608398

292. Talu M, Y�cel O, User Y, Dede A, �nder H, Tolun S. Oblique excision with primary closure for the treatment of pilonidal sinus. Eur J Plast Surg [Internet]. 1996 Jul;19(4):200–3. Available from: http://ovidsp.ovid.com/ovidweb.cgi?T=JS&PAGE=reference&D=emed6&NEWS=N&AN=26261577

293. H.K. S, G. C, I. C, M. O, I.H. O, R. Y. Karydakis flap reconstruction versus primary midline closure in chronic pilonidal disease. Eur Surg Res [Internet]. 2013;50(2 SUPPL. 4):209–10. Available from: http://ovidsp.ovid.com/ovidweb.cgi?T=JS&PAGE=reference&D=emed14&NEWS=N&AN=71095212

294. E. P, J. L. Recurrent pilonidal sinus disease. Color Dis [Internet]. 2016;18(Supplement 2):74. Available from: http://ovidsp.ovid.com/ovidweb.cgi?T=JS&PAGE=reference&D=emed17&NEWS=N&AN=612841552

295. P.C. P, D. P, B. F, S. K, J. F. Retrospective analysis of surgical treatment outcomes in pilonidal disease after cleft lift repair. Dis Colon Rectum [Internet]. 2018;61(5):e243. Available from: http://ovidsp.ovid.com/ovidweb.cgi?T=JS&PAGE=reference&D=emed19&NEWS=N&AN=622082306

296. N. P, D. A, A.C. I. Comparison between karydakis flap repair and primary closure for surgical treatment of sacrococcygeal pilonidal sinus. Trak Univ Tip Fak Derg [Internet]. 2008;25(2):87–94. Available from: http://www.tutfd.org/pdf/pdf_TTF_160.pdf

297. J. Y, G. W, M. D, D. M, T. M, S.J. A, et al. Outcomes following cleft closure for pilonidal sinus disease: A single centre experience. Color Dis [Internet]. 2012;14(SUPPL. 1):32. Available from: http://ovidsp.ovid.com/ovidweb.cgi?T=JS&PAGE=reference&D=emed13&NEWS=N&AN=70922402

298. N. T, M. G. Joint meeting of Sicilian, Calabrian and Sardinian Coloproctology Units “Modern management of anorectal sepsis.” Tech Coloproctol [Internet]. 2014 Apr 14;18(4):411–7. Available from: http://ovidsp.ovid.com/ovidweb.cgi?T=JS&PAGE=reference&D=emed15&NEWS=N&AN=71437568

299. Bulus H. Is Asymmetric Excision Primary Closure at Treatment of Pylonidal Sinus Disease an Appropriate Choice ? J Clin Anal Med [Internet]. 2011 Sep 1;2(3):79–81. Available from: http://www.jcam.com.tr/files/KATD-279.pdf

300. Dey A, Puri H, Malik VK. Management of pilonidal sinus disease by modified Karydakis procedure. Curr Med Res Pract [Internet]. 2014 Jul;4(4):156–60. Available from: http://www.journals.elsevier.com/current-medicine-research-and-practice/

301. J. Y, A. J, D. W, M. D, S.J. A, A. V. Recurrence rates following cleft closure for pilonidal sinus disease: A single centre experience. Color Dis [Internet]. 2013;15(SUPPL. 1):52. Available from: http://ovidsp.ovid.com/ovidweb.cgi?T=JS&PAGE=reference&D=emed14&NEWS=N&AN=71554243

302. Saber A. Modified off-midline closure of pilonidal sinus disease. N Am J Med Sci [Internet]. 2014;6(5):210. Available from: http://www.najms.org/temp/NorthAmJMedSci65210-1214302_032223.pdf

303. A. K, M.-S. F, A. K, A. M. Prospective evaluation of outcome of karydakis flap for sacrococcygeal pilonidal disease. Tehran Univ Med J [Internet]. 2015;72(12):823–30. Available from: http://tumj.tums.ac.ir/browse.php?a_id=6533&slc_lang=en&sid=1&ftxt=1

304. Keshvari A, Keramati MR, Fazeli MS, Kazemeini A, Nouritaromlou MK. Risk factors for complications and recurrence after the Karydakis flap. J Surg Res [Internet]. 2016;204(1):55–60. Available from: http://ovidsp.ovid.com/ovidweb.cgi?T=JS&PAGE=reference&D=med13&NEWS=N&AN=27451868

305. Mohamed SJ, Kristensen BB, Lindgaard L, Bisgaard T. Acceptable effect of multimodal analgesic treatment after a Bascom cleft lift operation. Dan Med J [Internet]. 2015;62(1):A4985. Available from: http://ovidsp.ovid.com/ovidweb.cgi?T=JS&PAGE=reference&D=med12&NEWS=N&AN=25557329

306. Kanat BH, Bozan MB, Yazar FM, Yur M, Erol F, Ozkan Z, et al. Comparison of early surgery (unroofing-curettage) and elective surgery (Karydakis flap technique) in pilonidal sinus abscess cases. Ulus Travma Acil Cerrahi Derg [Internet]. 2014;20(5):366–70. Available from: http://ovidsp.ovid.com/ovidweb.cgi?T=JS&PAGE=reference&D=med11&NEWS=N&AN=25541849

307. Dudink R, Veldkamp J, Nienhuijs S, Heemskerk J. Secondary healing versus midline closure and modified Bascom natal cleft lift for pilonidal sinus disease. Scand J Surg [Internet]. 2011;100(2):110–3. Available from: http://ovidsp.ovid.com/ovidweb.cgi?T=JS&PAGE=reference&D=med8&NEWS=N&AN=21737387

308. Can MF, Sevinc MM, Yilmaz M. Comparison of Karydakis flap reconstruction versus primary midline closure in sacrococcygeal pilonidal disease: results of 200 military service members. Surg Today [Internet]. 2009;39(7):580–6. Available from: http://ovidsp.ovid.com/ovidweb.cgi?T=JS&PAGE=reference&D=med7&NEWS=N&AN=19562445

309. Bertelsen CA, Jorgensen LN. [Bascom’s operation for pilonidal fistula]. Bascoms Oper pilonidalcyste [Internet]. 2008;170(26–32):2313–7. Available from: http://ovidsp.ovid.com/ovidweb.cgi?T=JS&PAGE=reference&D=med7&NEWS=N&AN=18570761

310. Abdelrazeq AS, Rahman M, Botterill ID, Alexander DJ. Short-term and long-term outcomes of the cleft lift procedure in the management of nonacute pilonidal disorders. Dis Colon Rectum [Internet]. 2008;51(7):1100–6. Available from: http://ovidsp.ovid.com/ovidweb.cgi?T=JS&PAGE=reference&D=med7&NEWS=N&AN=18470564

311. Petersen S, Aumann G, Kramer A, Doll D, Sailer M, Hellmich G. Short-term results of Karydakis flap for pilonidal sinus disease. Tech Coloproctol [Internet]. 2007;11(3):235–40. Available from: http://ovidsp.ovid.com/ovidweb.cgi?T=JS&PAGE=reference&D=med6&NEWS=N&AN=17676268

312. Mentes O, Bagci M, Bilgin T, Coskun I, Ozgul O, Ozdemir M. Management of pilonidal sinus disease with oblique excision and primary closure: results of 493 patients. Dis Colon Rectum [Internet]. 2006;49(1):104–8. Available from: http://ovidsp.ovid.com/ovidweb.cgi?T=JS&PAGE=reference&D=med6&NEWS=N&AN=16283563

313. Morden P, Drongowski RA, Geiger JD, Hirschl RB, Teitelbaum DH. Comparison of Karydakis versus midline excision for treatment of pilonidal sinus disease. Pediatr Surg Int [Internet]. 2005;21(10):793–6. Available from: http://ovidsp.ovid.com/ovidweb.cgi?T=JS&PAGE=reference&D=med6&NEWS=N&AN=16172873

314. Sakr M, El-Hammadi H, Moussa M, Arafa S, Rasheed M. The effect of obesity on the results of Karydakis technique for the management of chronic pilonidal sinus. Int J Colorectal Dis [Internet]. 2003;18(1):36–9. Available from: http://ovidsp.ovid.com/ovidweb.cgi?T=JS&PAGE=reference&D=med5&NEWS=N&AN=12458379

315. Immerman SC. Treatment of pilonidal disease using the Bascom “Cleft-Lift” procedure. Am Surg [Internet]. 2014;80(2):E49-50. Available from: http://ovidsp.ovid.com/ovidweb.cgi?T=JS&PAGE=reference&D=med11&NEWS=N&AN=24480199

316. Guner A, Ozkan OF, Kece C, Kesici S, Kucuktulu U. Modification of the Bascom cleft lift procedure for chronic pilonidal sinus: results in 141 patients. Colorectal Dis [Internet]. 2013;15(7):e402-6. Available from: http://ovidsp.ovid.com/ovidweb.cgi?T=JS&PAGE=reference&D=med10&NEWS=N&AN=23581906

317. Gendy AS, Glick RD, Hong AR, Dolgin SE, Soffer SZ, Landers H, et al. A comparison of the cleft lift procedure vs wide excision and packing for the treatment of pilonidal disease in adolescents. J Pediatr Surg [Internet]. 2011;46(6):1256–9. Available from: http://ovidsp.ovid.com/ovidweb.cgi?T=JS&PAGE=reference&D=med8&NEWS=N&AN=21683232

318. Kaplan M, Ozcan O, Bilgic E, Kaplan ET, Kaplan T, Kaplan FC. Distal scar-to-midline distance in pilonidal Limberg flap surgery is a recurrence-promoting factor: A multicenter, case-control study. Am J Surg [Internet]. 2017;214(5):811–9. Available from: http://ovidsp.ovid.com/ovidweb.cgi?T=JS&PAGE=reference&D=med14&NEWS=N&AN=28359560

319. Umesh V, Sussman RH, Smith J, Whyte C. Long term outcome of the Bascom cleft lift procedure for adolescent pilonidal sinus. J Pediatr Surg [Internet]. 2018;53(2):295–7. Available from: http://ovidsp.ovid.com/ovidweb.cgi?T=JS&PAGE=reference&D=med15&NEWS=N&AN=29223675

320. Ehrl D, Choplain C, Heidekrueger P, Erne HC, Rau H-G, Broer PN. Treatment Options for Pilonidal Disease. Am Surg [Internet]. 2017;83(5):453–7. Available from: http://ovidsp.ovid.com/ovidweb.cgi?T=JS&PAGE=reference&D=med14&NEWS=N&AN=28541853

321. Brusciano L, Limongelli P, Del Genio G, Tolone S, Amoroso V, Docimo G, et al. D-shape asymmetric excision of sacrococcygeal pilonidal sinus with primary closure, suction drain, and subcuticular skin closure: an analysis of risks factors for long-term recurrence. Surg Innov [Internet]. 2015;22(2):143–8. Available from: http://ovidsp.ovid.com/ovidweb.cgi?T=JS&PAGE=reference&D=med12&NEWS=N&AN=24902690

322. Yildiz MK, Ozkan E, Odabasi HM, Kaya B, Eris C, Abuoglu HH, et al. Karydakis flap procedure in patients with sacrococcygeal pilonidal sinus disease: experience of a single centre in Istanbul. ScientificWorldJournal [Internet]. 2013;2013:807027. Available from: http://ovidsp.ovid.com/ovidweb.cgi?T=JS&PAGE=reference&D=med10&NEWS=N&AN=23766710

323. Iesalnieks I, Deimel S, Schlitt HJ. Karydakis flap for recurrent pilonidal disease. World J Surg [Internet]. 2013;37(5):1115–20. Available from: http://ovidsp.ovid.com/ovidweb.cgi?T=JS&PAGE=reference&D=med10&NEWS=N&AN=23435676

324. Maghsoudi H, Nezami N, Ghamari AA. Ambulatory treatment of chronic pilonidal sinuses with lateral incision and primary suture. Can J Surg [Internet]. 2011;54(2):78–82. Available from: http://ovidsp.ovid.com/ovidweb.cgi?T=JS&PAGE=reference&D=med8&NEWS=N&AN=21251419

325. Rushfeldt C, Bernstein A, Norderval S, Revhaug A. Introducing an asymmetric cleft lift technique as a uniform procedure for pilonidal sinus surgery. Scand J Surg [Internet]. 2008;97(1):77–81. Available from: http://ovidsp.ovid.com/ovidweb.cgi?T=JS&PAGE=reference&D=med7&NEWS=N&AN=18450210

326. Borel F, Gaudin C, Duchalais E, Lehur P-A, Meurette G. Wound closure with Karydakis flap is decreasing the perioperative costs after pilonidal sinus excision as compared to lay-open approach. J Visc Surg [Internet]. 2017;154(6):407–12. Available from: http://ovidsp.ovid.com/ovidweb.cgi?T=JS&PAGE=reference&D=med14&NEWS=N&AN=29100740

327. Ortega PM, Baixauli J, Arredondo J, Bellver M, Sanchez-Justicia C, Ocana S, et al. Is the cleft lift procedure for non-acute sacrococcygeal pilonidal disease a definitive treatment? Long-term outcomes in 74 patients. Surg Today [Internet]. 2014;44(12):2318–23. Available from: http://ovidsp.ovid.com/ovidweb.cgi?T=JS&PAGE=reference&D=med11&NEWS=N&AN=24845740

328. Limongelli P, Brusciano L, Di Stazio C, del Genio G, Tolone S, Lucido FS, et al. D-shape asymmetric and symmetric excision with primary closure in the treatment of sacrococcygeal pilonidal disease. Am J Surg [Internet]. 2014;207(6):882–9. Available from: http://ovidsp.ovid.com/ovidweb.cgi?T=JS&PAGE=reference&D=med11&NEWS=N&AN=24112672

329. Buczacki S, Drage M, Wells A, Guy R. Sacrococcygeal pilonidal sinus disease. Colorectal Dis [Internet]. 2009;11(6):657. Available from: http://ovidsp.ovid.com/ovidweb.cgi?T=JS&PAGE=reference&D=med7&NEWS=N&AN=19250261

330. Majeski J, Stroud J. Sacrococcygeal pilonidal disease. Int Surg [Internet]. 2011;96(2):144–7. Available from: http://ovidsp.ovid.com/ovidweb.cgi?T=JS&PAGE=reference&D=med8&NEWS=N&AN=22026306

331. Moran DC, Kavanagh DO, Adhmed I, Regan MC. Excision and primary closure using the Karydakis flap for the treatment of pilonidal disease: outcomes from a single institution. World J Surg [Internet]. 2011;35(8):1803–8. Available from: http://ovidsp.ovid.com/ovidweb.cgi?T=JS&PAGE=reference&D=med8&NEWS=N&AN=21553200

332. Senapati A, Cripps NPJ, Flashman K, Thompson MR. Cleft closure for the treatment of pilonidal sinus disease. Colorectal Dis [Internet]. 2011;13(3):333–6. Available from: http://ovidsp.ovid.com/ovidweb.cgi?T=JS&PAGE=reference&D=med8&NEWS=N&AN=20015265

333. Inan A, Surgit O, Sen M, Bozer M, Dener C. One day surgery for pilonidal disease. Bratisl Lek Listy [Internet]. 2011;112(10):572–4. Available from: http://ovidsp.ovid.com/ovidweb.cgi?T=JS&PAGE=reference&D=med8&NEWS=N&AN=21954542

334. Favuzza J, Brand M, Francescatti A, Orkin B. Cleft lift procedure for pilonidal disease: technique and perioperative management. Tech Coloproctol [Internet]. 2015;19(8):477–82. Available from: http://ovidsp.ovid.com/ovidweb.cgi?T=JS&PAGE=reference&D=med12&NEWS=N&AN=26165209

335. Tezel E, Bostanci H, Anadol AZ, Kurukahvecioglu O. Cleft lift procedure for sacrococcygeal pilonidal disease. Dis Colon Rectum [Internet]. 2009;52(1):135–9. Available from: http://ovidsp.ovid.com/ovidweb.cgi?T=JS&PAGE=reference&D=med7&NEWS=N&AN=19273969

336. Anderson JH, Yip CO, Nagabhushan JS, Connelly SJ. Day-case Karydakis flap for pilonidal sinus. Dis Colon Rectum [Internet]. 2008;51(1):134–8. Available from: http://ovidsp.ovid.com/ovidweb.cgi?T=JS&PAGE=reference&D=med7&NEWS=N&AN=18193323

337. Bessa SS. Results of the lateral advancing flap operation (modified Karydakis procedure) for the management of pilonidal sinus disease. Dis Colon Rectum [Internet]. 2007;50(11):1935–40. Available from: http://ovidsp.ovid.com/ovidweb.cgi?T=JS&PAGE=reference&D=med6&NEWS=N&AN=17828399

338. Bascom J, Bascom T. Utility of the cleft lift procedure in refractory pilonidal disease. Am J Surg [Internet]. 2007;193(5):606–9. Available from: http://ovidsp.ovid.com/ovidweb.cgi?T=JS&PAGE=reference&D=med6&NEWS=N&AN=17434365

339. Keshava A, Young CJ, Rickard MJFX, Sinclair G. Karydakis flap repair for sacrococcygeal pilonidal sinus disease: how important is technique?. ANZ J Surg [Internet]. 2007;77(3):181–3. Available from: http://ovidsp.ovid.com/ovidweb.cgi?T=JS&PAGE=reference&D=med6&NEWS=N&AN=17305997

340. Abdul-Ghani AKM, Abdul-Ghani AN, Ingham Clark CL. Day-care surgery for pilonidal sinus. Ann R Coll Surg Engl [Internet]. 2006;88(7):656–8. Available from: http://ovidsp.ovid.com/ovidweb.cgi?T=JS&PAGE=reference&D=med6&NEWS=N&AN=17132316

341. Kulacoglu H, Dener C, Tumer H, Aktimur R. Total subcutaneous fistulectomy combined with Karydakis flap for sacrococcygeal pilonidal disease with secondary perianal opening. Colorectal Dis [Internet]. 2006;8(2):120–3. Available from: http://ovidsp.ovid.com/ovidweb.cgi?T=JS&PAGE=reference&D=med6&NEWS=N&AN=16412071

342. Theodoropoulos GE, Vlahos K, Lazaris AC, Tahteris E, Panoussopoulos D. Modified Bascom’s asymmetric midgluteal cleft closure technique for recurrent pilonidal disease: early experience in a military hospital. Dis Colon Rectum [Internet]. 2003;46(9):1286–91. Available from: http://ovidsp.ovid.com/ovidweb.cgi?T=JS&PAGE=reference&D=med5&NEWS=N&AN=12972977

343. Bascom J, Bascom T. Failed pilonidal surgery: new paradigm and new operation leading to cures. Arch Surg [Internet]. 2002;137(10):1146–51. Available from: http://ovidsp.ovid.com/ovidweb.cgi?T=JS&PAGE=reference&D=med4&NEWS=N&AN=12361421

344. Akinci OF, Coskun A, Uzunkoy A. Simple and effective surgical treatment of pilonidal sinus: asymmetric excision and primary closure using suction drain and subcuticular skin closure. Dis Colon Rectum [Internet]. 2000;43(5):701–7. Available from: http://ovidsp.ovid.com/ovidweb.cgi?T=JS&PAGE=reference&D=med4&NEWS=N&AN=10826434

345. Kitchen PR. Pilonidal sinus: experience with the Karydakis flap. Br J Surg [Internet]. 1996;83(10):1452–5. Available from: http://ovidsp.ovid.com/ovidweb.cgi?T=JS&PAGE=reference&D=med4&NEWS=N&AN=8944470

346. Bascom JU. Repeat pilonidal operations. Am J Surg [Internet]. 1987;154(1):118–22. Available from: http://ovidsp.ovid.com/ovidweb.cgi?T=JS&PAGE=reference&D=med2&NEWS=N&AN=3605509

347. Kitchen PR. Pilonidal sinus: excision and primary closure with a lateralised wound - the Karydakis operation. Aust N Z J Surg [Internet]. 1982;52(3):302–5. Available from: http://ovidsp.ovid.com/ovidweb.cgi?T=JS&PAGE=reference&D=med2&NEWS=N&AN=6954933

348. Karydakis GE. New approach to the problem of pilonidal sinus. Lancet (London, England) [Internet]. 1973;2(7843):1414–5. Available from: http://ovidsp.ovid.com/ovidweb.cgi?T=JS&PAGE=reference&D=med1&NEWS=N&AN=4128725

349. Gönenç M, Yirgin H, Dinç M, Kapan S, Turhan AN, Alis H. Karydakis flap for sacrococcygeal pilonidal sinus disease: long-term outcomes. A retrospective analysis. Med J Bakirkoy [Internet]. 2011 Dec;7(4):153–5. Available from: http://search.ebscohost.com/login.aspx?direct=true&db=cin20&AN=108223035&site=ehost-live

350. De Robles MS, Seyfi D, Zahid A, Young CJ. Karydakis procedure can be effectively performed in the lateral position. ANZ J Surg [Internet]. 2019 Jan;89(1–2):E10–4. Available from: http://ovidsp.ovid.com/ovidweb.cgi?T=JS&PAGE=reference&D=emexb&NEWS=N&AN=626356083

351. H.K. S, G. C, I. C. Karydakis flap reconstruction versus cleft lift procedure in pilonidal disease. Eur Surg Res [Internet]. 2014;52(3–4):207. Available from: http://ovidsp.ovid.com/ovidweb.cgi?T=JS&PAGE=reference&D=emed15&NEWS=N&AN=71493435

352. Hatch Q, Marenco C, Lammers D, Morte K, Schlussel A, McNevin S. Postoperative outcomes of Bascom cleft lift for pilonidal disease: A single-center experience. Am J Surg [Internet]. 2020;219(5):737–40. Available from: http://ovidsp.ovid.com/ovidweb.cgi?T=JS&PAGE=reference&D=medl&NEWS=N&AN=32223912

353. M.I. K, M. J, S. B, U. S. To evaluate the outcome of sacrococcygeal pilonidal sinus excision using karydakis technique. Med Forum Mon [Internet]. 2014;25(12):57–9. Available from: http://www.medforum.pk/

354. Ragab Raga A-M. Oblique Excision and Primary Closure of Pilonidal Sinus (Sacrococcygeal). Trends Med Res [Internet]. 2012 Feb 1;7(2):62–9. Available from: http://scialert.net/qredirect.php?doi=tmr.2012.62.69&linkid=pdf

355. F. S. Karydakis technique for pilonidal sinus: Revisited. Pakistan J Med Heal Sci [Internet]. 2017;11(2):742–4. Available from: http://www.pjmhsonline.com/2017/april_june/pdf/742.pdf

356. Barrera E. A, Pradenas B. S, Bannura C. G, Illanes F. F, Gallardo V. C, Rinaldi C. B, et al. Operación de Bascom para el tratamiento de la enfermedad pilonidal sacrococcígea abscedada. Experiencia inicial. Rev Chil Cirugía [Internet]. 2016 Aug;70(4):350–3. Available from: https://scielo.conicyt.cl/pdf/rchcir/v70n4/0718-4026-rchcir-70-04-0350.pdf

357. J. F, M. B, A. F, L. J, M. MP. Cleft lift procedure as first line for pilonidal disease. Dis Colon Rectum [Internet]. 2014;57(5):e124. Available from: http://ovidsp.ovid.com/ovidweb.cgi?T=JS&PAGE=reference&D=emed15&NEWS=N&AN=71493629

358. C. G, F. B, G. M. Karydakis flap: A simple way to decrease the cost of pilonidal disease management. Color Dis [Internet]. 2015;17(SUPPL. 2):93. Available from: http://ovidsp.ovid.com/ovidweb.cgi?T=JS&PAGE=reference&D=emed16&NEWS=N&AN=72057749

359. kurt feyzi. The comparison of Crystallized phenol with Lateral Flap method in treatment of sinus pilonidalis. East J Med [Internet]. 2019;24(4):422–6. Available from: https://www.journalagent.com/z4/download_fulltext.asp?pdir=ejm&plng=eng&un=EJM-92485

360. S. D. Patient satisfaction after Karydakis flap for pilonidal disease. Color Dis [Internet]. 2015;17(SUPPL. 2):91. Available from: http://ovidsp.ovid.com/ovidweb.cgi?T=JS&PAGE=reference&D=emed16&NEWS=N&AN=72057740

361. Brusciano L, Del Genio G, Tolone S, Schiano di Visconte M, Gualtieri G, Terracciano G, et al. D-shape asymmetric excision in recurrent pilonidalis disease: an analytic longitudinal long-term evaluation. Updates Surg [Internet]. 2019 Dec 18;71(4):723–7. Available from: http://ovidsp.ovid.com/ovidweb.cgi?T=JS&PAGE=reference&D=medl&NEWS=N&AN=30887467

362. V. S. Pilonidal disease - Individualization and pathogenesis-oriented surgery. Color Dis [Internet]. 2018;20(Supplement 4):138. Available from: http://ovidsp.ovid.com/ovidweb.cgi?T=JS&PAGE=reference&D=emed19&NEWS=N&AN=624185849

363. Ekici U, Ferhatoglu MF. The effects of skin closure by using mattress sutures or intracutaneous absorbable sutures after the Karydakis flap surgery because of sacrococcygeal pilonidal sinus. A comparative analysis. Ann Ital Chir [Internet]. 2019;90:474–9. Available from: http://ovidsp.ovid.com/ovidweb.cgi?T=JS&PAGE=reference&D=medl&NEWS=N&AN=31158103

364. Yardimci VH. Outcomes of Two Treatments for Uncomplicated Pilonidal Sinus Disease: Karydakis Flap Procedure and Sinus Tract Ablation Procedure Using a 1,470 nm Diode Laser Combined With Pit Excision. Lasers Surg Med [Internet]. 2020 Nov 17;52(9):848–54. Available from: http://onlinelibrary.wiley.com/journal/10.1002/(ISSN)1096-9101

365. J. E. Bascom’s Cleft Closure in the treatment of sacrococcygeal pilonidal disease. Dis Colon Rectum [Internet]. 2009;52(4):858–9. Available from: http://ovidsp.ovid.com/ovidweb.cgi?T=JS&PAGE=reference&D=emed11&NEWS=N&AN=70343005

366. S. M. Comparative analysis of Endoscopic Pilonidal Sinus Treatment (EPSiT) and non EPSiT therapies in an Asian population cohort. Color Dis [Internet]. 2017;19(Supplement 2):64. Available from: http://ovidsp.ovid.com/ovidweb.cgi?T=JS&PAGE=reference&D=emed18&NEWS=N&AN=618607694

367. Ciftci F, Abdurrahman I, Tosun M, Bas G. A new approach: oblique excision and primary closure in the management of acute pilonidal disease. Int J Clin Exp Med [Internet]. 2014;7(12):5706–10. Available from: http://www.ijcem.com/files/ijcem0003591.pdf

368. I. I, H.J. S. Karydakis flap for recurrent pilonidal disease. Color Dis [Internet]. 2010;12(SUPPL. 3):50. Available from: http://ovidsp.ovid.com/ovidweb.cgi?T=JS&PAGE=reference&D=emed11&NEWS=N&AN=70325315

369. S. A, S. R, F. E-M. Modified Karydakis flap for sacrococcygeal pilonidal sinus: Results of 7 years’ experience in a UK district hospital. Color Dis [Internet]. 2011;13(SUPPL. 6):50. Available from: http://ovidsp.ovid.com/ovidweb.cgi?T=JS&PAGE=reference&D=emed12&NEWS=N&AN=70567375

370. A. K, M.F. F, T. K, A.I. F, A. K, M. Y. A novel technique in sacrococcygeal pilonidal sinus disease: Effect of death space reduction with additional skin excision and asymmetric primary closure. Color Dis [Internet]. 2017;19(Supplement 2):57. Available from: http://ovidsp.ovid.com/ovidweb.cgi?T=JS&PAGE=reference&D=emed18&NEWS=N&AN=618607822

371. I. I, S. D. Postoperative wound infections after karydakis flap in patients with pilonidal disease. Dis Colon Rectum [Internet]. 2012;55(5):e158. Available from: http://ovidsp.ovid.com/ovidweb.cgi?T=JS&PAGE=reference&D=emed13&NEWS=N&AN=71634853

372. De Nardi P, Gazzetta PG, Fiorentini G, Guarneri G. The cleft lift procedure for complex pilonidal disease. Eur Surg [Internet]. 2016 Aug 16;48(4):250–7. Available from: http://www.springerlink.com/content/1682-8631

373. Toydemir T, Peşluk O, Ermeç ED, Turhan AN. Sakrokosigeal Pilonidal Sinüs Hastalığının Cerrahi Tedavisinde Karydakis Flap ile Primer Kapama Prosedürlerinin Klinik Sonuçlarının Karşılaştırılması. Bakirkoy Tip Derg / Med J Bakirkoy [Internet]. 2012 Jun 15;8(2):78–81. Available from: http://search.ebscohost.com/login.aspx?direct=true&db=cin20&AN=108145428&site=ehost-live

374. C.W. M, M. M, D. L, K. M. Perioperative outcomes of bascom cleft lift for pilonidal disease: A single-center retrospective review. Dis Colon Rectum [Internet]. 2019;62(6):e340. Available from: http://ovidsp.ovid.com/ovidweb.cgi?T=JS&PAGE=reference&D=emexa&NEWS=N&AN=628086238

375. Fitzpatrick EB, Chesley PM, Oguntoye MO, Maykel JA, Johnson EK, Steele SR. Pilonidal disease in a military population: how far have we really come? Am J Surg [Internet]. 2014 Jun;207(6):907–14. Available from: http://ovidsp.ovid.com/ovidweb.cgi?T=JS&PAGE=reference&D=emed14&NEWS=N&AN=70973429

376. A. K, M. F, A. K, A. M. Risk factors of early wound failures in karydakis flap for sacral pilonidal sinus. Dis Colon Rectum [Internet]. 2011;54(5):e98--e99. Available from: http://ovidsp.ovid.com/ovidweb.cgi?T=JS&PAGE=reference&D=emed12&NEWS=N&AN=71635264

377. Demircan F, Akbulut S, Yavuz R, Agtas H, Karabulut K, Yagmur Y. The effect of laser epilation on recurrence and satisfaction in patients with sacrococcygeal pilonidal disease: a prospective randomized controlled trial. Int J Clin Exp Med [Internet]. 2015;8(2):2929–33. Available from: http://www.ijcem.com/files/ijcem0003594.pdf

378. F. P. Excision of the fistula tract with tension-free primary closure for pilonidal disease. Color Dis [Internet]. 2012;14(SUPPL. 2):63. Available from: http://ovidsp.ovid.com/ovidweb.cgi?T=JS&PAGE=reference&D=emed13&NEWS=N&AN=70926539

379. Dutkiewicz P, Ciesielski P, Kołodziejczak M. Results of surgical treatment of pilonidal sinus in 50 patients operated by the Bascom 2 method – prospective study. Polish J Surg [Internet]. 2019 Aug 31;91(5):1–5. Available from: http://ovidsp.ovid.com/ovidweb.cgi?T=JS&PAGE=reference&D=medl&NEWS=N&AN=31702572

380. I. I, S. D, C. Z. Karydakis flap for recurrent pilonidal disease. Color Dis [Internet]. 2012;14(SUPPL. 2):27. Available from: http://ovidsp.ovid.com/ovidweb.cgi?T=JS&PAGE=reference&D=emed13&NEWS=N&AN=70926257

381. McCallum I, King PM, Bruce J. Healing by primary versus secondary intention after surgical treatment for pilonidal sinus. Cochrane database Syst Rev [Internet]. 2007;(4):CD006213. Available from: http://ovidsp.ovid.com/ovidweb.cgi?T=JS&PAGE=reference&D=med6&NEWS=N&AN=17943897

382. Al-Khamis A, McCallum I, King PM, Bruce J. Healing by primary versus secondary intention after surgical treatment for pilonidal sinus. Cochrane database Syst Rev [Internet]. 2010;(1):CD006213. Available from: http://ovidsp.ovid.com/ovidweb.cgi?T=JS&PAGE=reference&D=med8&NEWS=N&AN=20091589

383. Milone M, Musella M, Maietta P, Bianco P, Taffuri C, Salvatore G, et al. Intradermal absorbable sutures to close pilonidal sinus wounds: a safe closure method?. Surg Today [Internet]. 2014;44(9):1638–42. Available from: http://ovidsp.ovid.com/ovidweb.cgi?T=JS&PAGE=reference&D=med11&NEWS=N&AN=24078028

384. Aldaqal SM, Kensarah AA, Alhabboubi M, Ashy AA. A new technique in management of pilonidal sinus, a university teaching hospital experience. Int Surg [Internet]. 2013;98(4):304–6. Available from: http://ovidsp.ovid.com/ovidweb.cgi?T=JS&PAGE=reference&D=med10&NEWS=N&AN=24229013

385. Lorant T, Ribbe I, Mahteme H, Gustafsson U-M, Graf W. Sinus excision and primary closure versus laying open in pilonidal disease: a prospective randomized trial. Dis Colon Rectum [Internet]. 2011;54(3):300–5. Available from: http://ovidsp.ovid.com/ovidweb.cgi?T=JS&PAGE=reference&D=med8&NEWS=N&AN=21304300

386. Rao MM, Zawislak W, Kennedy R, Gilliland R. A prospective randomised study comparing two treatment modalities for chronic pilonidal sinus with a 5-year follow-up. Int J Colorectal Dis [Internet]. 2010;25(3):395–400. Available from: http://ovidsp.ovid.com/ovidweb.cgi?T=JS&PAGE=reference&D=med8&NEWS=N&AN=19823853

387. Tocchi A, Mazzoni G, Bononi M, Fornasari V, Miccini M, Drumo A, et al. Outcome of chronic pilonidal disease treatment after ambulatory plain midline excision and primary suture. Am J Surg [Internet]. 2008;196(1):28–33. Available from: http://ovidsp.ovid.com/ovidweb.cgi?T=JS&PAGE=reference&D=med7&NEWS=N&AN=18565338

388. Al-Salamah SM, Hussain MI, Mirza SM. Excision with or without primary closure for pilonidal sinus disease. J Pak Med Assoc [Internet]. 2007;57(8):388–91. Available from: http://ovidsp.ovid.com/ovidweb.cgi?T=JS&PAGE=reference&D=med6&NEWS=N&AN=17902520

389. Kareem TS. Surgical treatment of chronic sacrococcygeal pilonidal sinus. Open method versus primary closure. Saudi Med J [Internet]. 2006;27(10):1534–7. Available from: http://ovidsp.ovid.com/ovidweb.cgi?T=JS&PAGE=reference&D=med6&NEWS=N&AN=17013478

390. Yigit T, Yigitler C, Gulec B, Ihsan UA, Ozer T, Oner K. Do we need to use subcutaneous suture for pilonidal sinus treated with excision and simple primary closure?. Acta Chir Belg [Internet]. 2005;105(6):635–8. Available from: http://ovidsp.ovid.com/ovidweb.cgi?T=JS&PAGE=reference&D=med6&NEWS=N&AN=16438075

391. Al-Naami MY. Outpatient pilonidal sinotomy complemented with good wound and surrounding skin care. Saudi Med J [Internet]. 2005;26(2):285–8. Available from: http://ovidsp.ovid.com/ovidweb.cgi?T=JS&PAGE=reference&D=med6&NEWS=N&AN=15770307

392. Gencosmanoglu R, Inceoglu R. Modified lay-open (incision, curettage, partial lateral wall excision and marsupialization) versus total excision with primary closure in the treatment of chronic sacrococcygeal pilonidal sinus: a prospective, randomized clinical trial with a complete two. Int J Colorectal Dis [Internet]. 2005;20(5):415–22. Available from: http://ovidsp.ovid.com/ovidweb.cgi?T=JS&PAGE=reference&D=med6&NEWS=N&AN=15714292

393. Holzer B, Grussner U, Bruckner B, Houf M, Kiffner E, Schildberg FW, et al. Efficacy and tolerance of a new gentamicin collagen fleece (Septocoll) after surgical treatment of a pilonidal sinus. Colorectal Dis [Internet]. 2003;5(3):222–7. Available from: http://ovidsp.ovid.com/ovidweb.cgi?T=JS&PAGE=reference&D=med5&NEWS=N&AN=12780882

394. Miocinovic M, Horzic M, Bunoza D. The treatment of pilonidal disease of the sacrococcygeal region by the method of limited excision and open wound healing. Acta Med Croatica [Internet]. 2000;54(1):27–31. Available from: http://ovidsp.ovid.com/ovidweb.cgi?T=JS&PAGE=reference&D=med4&NEWS=N&AN=10914438

395. Sondenaa K, Nesvik I, Andersen E, Soreide JA. Recurrent pilonidal sinus after excision with closed or open treatment: final result of a randomised trial. Eur J Surg [Internet]. 1996;162(3):237–40. Available from: http://ovidsp.ovid.com/ovidweb.cgi?T=JS&PAGE=reference&D=med4&NEWS=N&AN=8695740

396. Fuzun M, Bakir H, Soylu M, Tansug T, Kaymak E, Harmancioglu O. Which technique for treatment of pilonidal sinus--open or closed?. Dis Colon Rectum [Internet]. 1994;37(11):1148–50. Available from: http://ovidsp.ovid.com/ovidweb.cgi?T=JS&PAGE=reference&D=med3&NEWS=N&AN=7956585

397. Khawaja HT, Bryan S, Weaver PC. Treatment of natal cleft sinus: a prospective clinical and economic evaluation. BMJ [Internet]. 1992;304(6837):1282–3. Available from: http://ovidsp.ovid.com/ovidweb.cgi?T=JS&PAGE=reference&D=med3&NEWS=N&AN=1606429

398. Sondenaa K, Andersen E, Soreide JA. Morbidity and short term results in a randomised trial of open compared with closed treatment of chronic pilonidal sinus. Eur J Surg [Internet]. 1992;158(6–7):351–5. Available from: http://ovidsp.ovid.com/ovidweb.cgi?T=JS&PAGE=reference&D=med3&NEWS=N&AN=1356467

399. al-Hassan HK, Francis IM, Neglen P. Primary closure or secondary granulation after excision of pilonidal sinus?. Acta Chir Scand [Internet]. 1990;156(10):695–9. Available from: http://ovidsp.ovid.com/ovidweb.cgi?T=JS&PAGE=reference&D=med3&NEWS=N&AN=2264427

400. Kronborg O, Christensen K, Zimmermann-Nielsen C. Chronic pilonidal disease: a randomized trial with a complete 3-year follow-up. Br J Surg [Internet]. 1985;72(4):303–4. Available from: http://ovidsp.ovid.com/ovidweb.cgi?T=JS&PAGE=reference&D=med2&NEWS=N&AN=3886069

401. Rossi P, Russo F, Gentileschi P, Quintigliano D, Cicardo G, Nasrollah N, et al. [The pilonidal sinus: its surgical treatment, our experience and a review of the literature]. Sinus pilonidalis Tratt Chir nostra Esper e Revis della Lett [Internet]. 1993;14(2):120–3. Available from: http://ovidsp.ovid.com/ovidweb.cgi?T=JS&PAGE=reference&D=med3&NEWS=N&AN=8489894

402. Williams RS. A simple technique for successful primary closure after excision of pilonidal sinus disease. Ann R Coll Surg Engl [Internet]. 1990;72(5):313–5. Available from: http://ovidsp.ovid.com/ovidweb.cgi?T=JS&PAGE=reference&D=med3&NEWS=N&AN=2221767

403. Pozzi C, Tritapepe R. [Primary therapy after radical removal of sinus pilonidal]. Guarigione per prima Intenz dopo Interv Radic di asportazione del sinus pilonidalis [Internet]. 1989;44(22):2329–32. Available from: http://ovidsp.ovid.com/ovidweb.cgi?T=JS&PAGE=reference&D=med3&NEWS=N&AN=2626198

404. Mann C V, Springall R. “D” excision for sacrococcygeal pilonidal sinus disease. J R Soc Med [Internet]. 1987;80(5):292–5. Available from: http://ovidsp.ovid.com/ovidweb.cgi?T=JS&PAGE=reference&D=med2&NEWS=N&AN=3302254

405. Zimmerman CE. Outpatient excision and primary closure of pilonidal cysts and sinuses. Long-term follow-up. Am J Surg [Internet]. 1984;148(5):658–9. Available from: http://ovidsp.ovid.com/ovidweb.cgi?T=JS&PAGE=reference&D=med2&NEWS=N&AN=6496858

406. Rainsbury RM, Southam JA. Radical surgery for pilonidal sinus. Ann R Coll Surg Engl [Internet]. 1982;64(5):339–41. Available from: http://ovidsp.ovid.com/ovidweb.cgi?T=JS&PAGE=reference&D=med2&NEWS=N&AN=7114771

407. Farringer JLJ, Pickens DRJ. Pilonidal cyst: an operative approach. Am J Surg [Internet]. 1978;135(2):262–4. Available from: http://ovidsp.ovid.com/ovidweb.cgi?T=JS&PAGE=reference&D=med1&NEWS=N&AN=626307

408. Appiani GP. [Removal and immediate suture of sacrococcygeal cysts]. L’asportazione con sutura Immed delle Cist sacro-coccigee [Internet]. 1978;33(21):1619–22. Available from: http://ovidsp.ovid.com/ovidweb.cgi?T=JS&PAGE=reference&D=med1&NEWS=N&AN=364336

409. Zimmerman CE. Outpatient excision and primary closure of pilonidal cysts and sinuses. Am J Surg [Internet]. 1978;136(5):640–2. Available from: http://ovidsp.ovid.com/ovidweb.cgi?T=JS&PAGE=reference&D=med1&NEWS=N&AN=360863

410. Abramson DJ. Excision and delayed closure of pilonidal sinuses. Surg Gynecol Obstet [Internet]. 1977;144(2):205–7. Available from: http://ovidsp.ovid.com/ovidweb.cgi?T=JS&PAGE=reference&D=med1&NEWS=N&AN=835059

411. Bentivegna SS, Procario P. Primary closure of pilonidal cystectomy. Am Surg [Internet]. 1977;43(4):214–6. Available from: http://ovidsp.ovid.com/ovidweb.cgi?T=JS&PAGE=reference&D=med1&NEWS=N&AN=322559

412. Kam BH. A simple surgical method of treating pilonidal sinus. Arch Chir Neerl [Internet]. 1976;28(1):43–53. Available from: http://ovidsp.ovid.com/ovidweb.cgi?T=JS&PAGE=reference&D=med1&NEWS=N&AN=938060

413. Cruz J, Ram MD. Sacrococcygeal pilonidal sinus. Dis Colon Rectum [Internet]. 1971;14(5):356–9. Available from: http://ovidsp.ovid.com/ovidweb.cgi?T=JS&PAGE=reference&D=med1&NEWS=N&AN=4938034

414. ROSS RL. Recurrent pilonidal cyst and sinus; a plan of preoperative preparation, operation and postoperative care. Calif Med [Internet]. 1956;85(3):183–6. Available from: http://ovidsp.ovid.com/ovidweb.cgi?T=JS&PAGE=reference&D=med1&NEWS=N&AN=13356182

415. CLOSE AS. Pilonidal cysts: an analysis of surgical failures. Ann Surg [Internet]. 1955;141(4):523–6. Available from: http://ovidsp.ovid.com/ovidweb.cgi?T=JS&PAGE=reference&D=med1&NEWS=N&AN=14362385

416. Courtney SP, Merlin MJ. The use of fusidic acid gel in pilonidal abscess treatment: cure, recurrence and failure rates. Ann R Coll Surg Engl [Internet]. 1986;68(3):170–1. Available from: http://ovidsp.ovid.com/ovidweb.cgi?T=JS&PAGE=reference&D=med2&NEWS=N&AN=3729269

417. Bascom J. Pilonidal disease: origin from follicles of hairs and results of follicle removal as treatment. Surgery [Internet]. 1980;87(5):567–72. Available from: http://ovidsp.ovid.com/ovidweb.cgi?T=JS&PAGE=reference&D=med2&NEWS=N&AN=7368107

418. GOSWITZ JT. SACROCOCCYGEAL PILONIDAL SINUS DISEASE. TREATMENT AND LONG-TERM FOLLOW-UP IN A MILITARY HOSPITAL SERVING COMBAT-READY UNITS. Arch Surg [Internet]. 1965;90:890–2. Available from: http://ovidsp.ovid.com/ovidweb.cgi?T=JS&PAGE=reference&D=med1&NEWS=N&AN=14333532

419. GOODALL P. The aetiology and treatment of pilonidal sinus. A review of 163 patients. Br J Surg [Internet]. 1961;49:212–8. Available from: http://ovidsp.ovid.com/ovidweb.cgi?T=JS&PAGE=reference&D=med1&NEWS=N&AN=13900261

420. HARBERSON JC, BRINTNALL ES. Surgical principles in the treatment of pilonidal cyst and its complications. Am J Surg [Internet]. 1950;79(1):101-illust. Available from: http://ovidsp.ovid.com/ovidweb.cgi?T=JS&PAGE=reference&D=med1&NEWS=N&AN=15399359

421. H.R. H, R. G. Recurrence rate in the pilonidal sinus after excision with or without primary closure. Koomesh [Internet]. 2013;15(1):78–82. Available from: http://koomeshjournal.semums.ac.ir/browse.php?a_id=1918&slc_lang=en&sid=1&ftxt=1

422. Arslan S, Karadeniz E, Ozturk G, Aydinli B, Bayraktutan MC, Atamanalp SS. Modified Primary Closure Method for the Treatment of Pilonidal Sinus. Eurasian J Med [Internet]. 2016 Jun;48(2):84–9. Available from: http://search.ebscohost.com/login.aspx?direct=true&db=cin20&AN=117408751&site=ehost-live

423. A. J, K. L, T.A. B. Excision and primacy closure of pilonidal sinus defects. Med Channel [Internet]. 2012;19(2):100–2. Available from: http://ovidsp.ovid.com/ovidweb.cgi?T=JS&PAGE=reference&D=emed13&NEWS=N&AN=366155335

424. M.F. C, K.M. G, Y.M. S, D.M. C. Comparative study of treatment methods of pilonidal sinus. New Armen Med J [Internet]. 2016;10(4):61–5. Available from: http://www.ysmu.am/images/stories/downloads/NAMJ/Int

425. F. S, S. L, L. S, R. L. Ambulatory treatment of pilonidal sinus. Chirurgia (Bucur) [Internet]. 1994;7(9):639–42. Available from: http://ovidsp.ovid.com/ovidweb.cgi?T=JS&PAGE=reference&D=emed5&NEWS=N&AN=24343839

426. M. M, M. M. Video assisted ablation of pilonidal sinus. A new endoscopic minimally invasive treatment. Preliminary results by a prospective comparative study. Surg Endosc Other Interv Tech [Internet]. 2015;29(SUPPL. 1):S52. Available from: http://ovidsp.ovid.com/ovidweb.cgi?T=JS&PAGE=reference&D=emed16&NEWS=N&AN=71872462

427. A.S. DM, D. H, M. D, A. R, E. T. Retrospective analysis of treatment of pilonidal sinus in a general surgery unit of a second level new creation hospital (2004-2008). Color Dis [Internet]. 2009;11(SUPPL. 2):38. Available from: http://ovidsp.ovid.com/ovidweb.cgi?T=JS&PAGE=reference&D=emed11&NEWS=N&AN=70204620

428. Benfatto G, Zanghi G, Catalano F, Benfatto S, Licari V, Basile GP, et al. [The use of drainage in the “per primam” treatment of recurrent pilonidal cysts]. G Chir [Internet]. 2003 May;24(5):205–8. Available from: http://ovidsp.ovid.com/ovidweb.cgi?T=JS&PAGE=reference&D=emed8&NEWS=N&AN=137569499

429. S.S. S. Pilonidal sinus: Following the Aristotelian middle! Pakistan J Med Sci [Internet]. 2008;24(6):845–8. Available from: http://www.pjms.com.pk/issues/octdec208/pdf/article13.pdf

430. B. S, M. M, A.A.S. K, R. S. A comparison between the outcomes of tie-over and closed suction drainage techniques in the management of sacrococcygeal pilonidal sinus disease. J Isfahan Med Sch [Internet]. 2012;29(161). Available from: http://jims.mui.ac.ir/index.php/jims/issue/archive

431. A.B. Z, M. A. Study of complications and patient satisfaction of pilonidal disease patients with tie over surgery versus open surgical technique in the razi hospital in 2010. Int J Pharm Technol [Internet]. 2016;8(1):11361–71. Available from: http://www.ijptonline.com/wp-content/uploads/2016/06/11361-11371.pdf

432. Oliveira AI, Barroso C, Osório A, Correia-Pinto J. Minimally Invasive Surgical Treatment of Pilonidal Disease: Mid-Term Retrospective Analysis of a Single Center. Front Pediatr [Internet]. 2019 Jun 4;7(JUN):215. Available from: https://www.frontiersin.org/journals/pediatrics

433. Maghsudi H, Almasi H, Mousavai Toomatari SE, Fasihi M, Akhavan Salamat S, Mousavi Toomatari SB, et al. Comparison of Primary Closure, Secondary Closure, and Limberg Flap in the Surgical Treatment of Pilonidal Cysts. Plast Surg Nurs [Internet]. 2020 Apr;40(2):81–5. Available from: http://search.ebscohost.com/login.aspx?direct=true&db=cin20&AN=144349355&site=ehost-live

434. M. G, T. T, G. G, P. S. Regenerative medicine for the definitive surgical repair of pilonidal sinus. A new method of wound reconstruction. Tech Coloproctol [Internet]. 2011;15(2):237–8. Available from: http://ovidsp.ovid.com/ovidweb.cgi?T=JS&PAGE=reference&D=emed12&NEWS=N&AN=70705949

435. M. A, M.H. R. Surgical treatment of chronic pilonidal sinus-open vs closed excision. J Coll Physicians Surg Pakistan [Internet]. 1999;9(12):518–21. Available from: http://ovidsp.ovid.com/ovidweb.cgi?T=JS&PAGE=reference&D=emed6&NEWS=N&AN=30120321

436. Baier PK, Baumgartner U, Furtwängler A, Holzinger F, Schöffel U. Die Therapie des Sinus pilonidalis - Primärer Wundverschluss versus offene Wundbehandlung nach Exzision -. Zentralbl Chir [Internet]. 2002;127(4):310–4. Available from: http://ovidsp.ovid.com/ovidweb.cgi?T=JS&PAGE=reference&D=emed7&NEWS=N&AN=34578695

437. S. M, H.A. M, S. M, S. A. Comparison of outcomes in four different surgical methods for sacral pilonidal sinus with long-term follows-up. Tehran Univ Med J [Internet]. 2019;76(10):660–4. Available from: http://tumj.tums.ac.ir/files/site1/user_files_40de64/tumj1-A-10-3666-110-bbc1b7f.pdf

438. A. C. Sinus pilonidalis: Removal and primary suture with aspirative draining. Chirurgia (Bucur) [Internet]. 1990;3(9):433–7. Available from: http://ovidsp.ovid.com/ovidweb.cgi?T=JS&PAGE=reference&D=emed4&NEWS=N&AN=20358876

439. T.W. K. Recurrence after excision and primary closure of pilonidal sinus. Pakistan J Med Sci [Internet]. 2007;23(3):375–9. Available from: http://www.pjms.com.pk/issues/aprjun207/pdf/pilonidal.pdf

440. J. S, E. T, E. M, Z. A-H, E. C. Primary closure vs excision and healing by secondary intention in the treatment of sacrococcygeal pilonidal disease. Ir J Med Sci [Internet]. 2011;180(SUPPL. 3):S95. Available from: http://ovidsp.ovid.com/ovidweb.cgi?T=JS&PAGE=reference&D=emed12&NEWS=N&AN=70701440

441. Salih AM, Kakamad FH, Salih RQ, Mohammed SH, Habibullah IJ, Hammood ZD, et al. Nonoperative management of pilonidal sinus disease: one more step toward the ideal management therapy—a randomized controlled trial. Surgery [Internet]. 2018 Jul;164(1):66–70. Available from: http://www.elsevier.com/inca/publications/store/6/2/3/1/9/5/index.htt

442. Y. K, I. T, D. S, D. M. Gluteus maximus fascia plasty (GMF) flap for pilonidal sinus is a promising technique to avoid recurrence. Color Dis [Internet]. 2018;20(Supplement 4):134. Available from: http://ovidsp.ovid.com/ovidweb.cgi?T=JS&PAGE=reference&D=emed19&NEWS=N&AN=624186221

443. M.A. A, G. H, I. A. “Pilonidal Sinus” Mass Closure (with Prolene no. 1 having 90mm needle) after simple excision. Pakistan J Med Heal Sci [Internet]. 2019;13(2):304–5. Available from: http://www.pjmhsonline.com/2019/april_june/pdf/304.pdf

444. Excision and primary closure of pilonidal sinus as day case. Ambul Surg [Internet]. 2015;21(3):108. Available from: http://www.iaas-med.com/files/Journal/21.3/AMB_SURG_21-33.pdf

445. M. C, K. G, Y. S, D. C. VAC therapy in the treatment of pilonidal disease. Color Dis [Internet]. 2017;19(Supplement 2):129. Available from: http://ovidsp.ovid.com/ovidweb.cgi?T=JS&PAGE=reference&D=emed18&NEWS=N&AN=618607589

446. Gul VO, Destek S. Sinusectomy and primary closure versus excision and primary closure in pilonidal sinus disease: a retrospective cohort study. Int J Colorectal Dis [Internet]. 2020 Jun 4;35(6):1117–24. Available from: http://link.springer.de/link/service/journals/00384/index.htm

447. Topuz Ö, Sözen S, Tükenmez M, Topuz S, Vurdem ÜE. Crystallized Phenol Treatment of Pilonidal Disease Improves Quality of Life. Indian J Surg [Internet]. 2014 Feb;76(1):81–4. Available from: http://search.ebscohost.com/login.aspx?direct=true&db=cin20&AN=103936258&site=ehost-live

448. Othman I. Skin Glue Improves Outcome after Excision and Primary Closure of Sacrococcygeal Pilonidal Disease. Indian J Surg [Internet]. 2010 Dec 16;72(6):470–4. Available from: http://search.ebscohost.com/login.aspx?direct=true&db=cin20&AN=104528388&site=ehost-live

449. G. L, G. M, A. N, T. B, C. S, S. S, et al. Sinus pilonidalis. Critical review of our experience. Chirurgia (Bucur) [Internet]. 1998;11(2):93–6. Available from: http://ovidsp.ovid.com/ovidweb.cgi?T=JS&PAGE=reference&D=emed6&NEWS=N&AN=28365264

450. The primary suture repair after pilonidal sinus excision using rubber tube. Dis Colon Rectum [Internet]. 2014;57(5):e260. Available from: http://ovidsp.ovid.com/ovidweb.cgi?T=JS&PAGE=reference&D=emed15&NEWS=N&AN=71493862

451. D. D, P. L, P. M. Minimally invasive treatment versus total excision with primary closure for pilonidal disease. Color Dis [Internet]. 2018;20(Supplement 4):131. Available from: http://ovidsp.ovid.com/ovidweb.cgi?T=JS&PAGE=reference&D=emed19&NEWS=N&AN=624186025

452. Khattak MI, Shah HU, Qazi D e. S. Pilonidal sinus; primary repair versus healing by second intention. Rawal Med J [Internet]. 2016;41(4):454–8. Available from: http://www.ejmanager.com/mnstemps/27/27-1449812059.pdf?t=1480298032

453. S. P, D. C. Risk factor analysis for surgical site infection after excision and primary closure of pilonidal sinus. Color Dis [Internet]. 2009;11(SUPPL. 2):58. Available from: http://ovidsp.ovid.com/ovidweb.cgi?T=JS&PAGE=reference&D=emed11&NEWS=N&AN=70204770

454. M.O. K, M.I. A, S.H. A, O. A. Comparison of “excision and primary repair” with “Bascom’s technique” in the surgical treatment of pilonidal disease. Int J Surg [Internet]. 2017;47(Supplement 1):S33. Available from: http://ovidsp.ovid.com/ovidweb.cgi?T=JS&PAGE=reference&D=emed18&NEWS=N&AN=619899347

455. M.G. M, C. M, M. C, A. C, P. M, M. S. Muzi’s tension free primary closure of pilonidal sinus disease: Long-term results on 450 patients. Gastroenterology [Internet]. 2017;152(5 Supplement 1):S1212--S1213. Available from: http://ovidsp.ovid.com/ovidweb.cgi?T=JS&PAGE=reference&D=emed18&NEWS=N&AN=618672593

456. A. W, K. G. Surgical treatment of pilonidal disease: Comparison of midline excision with primary closure and Bascom’s natal cleft closure. Color Dis [Internet]. 2009;11(SUPPL. 2):32. Available from: http://ovidsp.ovid.com/ovidweb.cgi?T=JS&PAGE=reference&D=emed11&NEWS=N&AN=70204573

457. Masuda H, Taniguchi T, Hayashi I, Kono Y, Watanabe K, Horiuchi H, et al. Factors influencing the results of surgery on the pilonidal sinus, with special reference to postoperative care. Nippon Daicho Komonbyo Gakkai Zasshi [Internet]. 1988;41(4):406–10. Available from: http://ovidsp.ovid.com/ovidweb.cgi?T=JS&PAGE=reference&D=emed4&NEWS=N&AN=18189698

458. Z.U. O, H. O, M. S, I.T. S. Comparison of primary repair and limberg flap technique in pilonidal sinus disease. Eur Surg Res [Internet]. 2013;50(SUPPL. 1):104–5. Available from: http://ovidsp.ovid.com/ovidweb.cgi?T=JS&PAGE=reference&D=emed14&NEWS=N&AN=71094754

459. A retrospective study of 103 patients who had pilonidal sinus surgery with direct suture. Tech Coloproctol [Internet]. 2014;18(4):411. Available from: http://ovidsp.ovid.com/ovidweb.cgi?T=JS&PAGE=reference&D=emed15&NEWS=N&AN=71437555

460. Z. H, A. A. Two step management of acute pilonidal sinus abscess. Color Dis [Internet]. 2011;13(SUPPL. 4):45. Available from: http://ovidsp.ovid.com/ovidweb.cgi?T=JS&PAGE=reference&D=emed12&NEWS=N&AN=70566049

461. E. N, I. B, G. E. Suction drain usage in treatment of pilonidal sinus disease. Turkish J Surg [Internet]. 2005;21(2):85–7. Available from: http://www.turkjsurg.com/

462. H. M, R. G. Bascom cleft closure is a simple and effective day-case procedure for sacrococcygeal pilonidal sinus. Color Dis [Internet]. 2014;16(SUPPL. 2):199–200. Available from: http://ovidsp.ovid.com/ovidweb.cgi?T=JS&PAGE=reference&D=emed15&NEWS=N&AN=71604844

463. Khodakaram K, Stark J, Hoglund I, Andersson RE. Minimal Excision and Primary Suture is a Cost-Efficient Definitive Treatment for Pilonidal Disease with Low Morbidity: A Population-Based Interventional and a Cross-Sectional Cohort Study. World J Surg [Internet]. 2017;41(5):1295–302. Available from: http://ovidsp.ovid.com/ovidweb.cgi?T=JS&PAGE=reference&D=med14&NEWS=N&AN=27905018

464. Muzi MG, Milito G, Nigro C, Cadeddu F, Farinon AM. A modification of primary closure for the treatment of pilonidal disease in day-care setting. Colorectal Dis [Internet]. 2009;11(1):84–8. Available from: http://ovidsp.ovid.com/ovidweb.cgi?T=JS&PAGE=reference&D=med7&NEWS=N&AN=18462226

465. Reboa G, Gipponi M, Testa T, Giannini G, Scala M, Dalla Costa R, et al. Regenerative medicine for the definitive surgical repair of pilonidal sinus. A new method of wound reconstruction. In Vivo [Internet]. 2007;21(3):529–34. Available from: http://ovidsp.ovid.com/ovidweb.cgi?T=JS&PAGE=reference&D=med6&NEWS=N&AN=17591365

466. Dalenback J, Magnusson O, Wedel N, Rimback G. Prospective follow-up after ambulatory plain midline excision of pilonidal sinus and primary suture under local anaesthesia--efficient, sufficient, and persistent. Colorectal Dis [Internet]. 2004;6(6):488–93. Available from: http://ovidsp.ovid.com/ovidweb.cgi?T=JS&PAGE=reference&D=med5&NEWS=N&AN=15521941

467. Calcina G, Setti P, Benati L, Savioli A, Galli G. [Excision and immediate suture technic in the treatment of pilonidal fistula. Our experience]. La Tec di escissione e sutura Immed nel Tratt della fistola pilonidale Nostra esperienza [Internet]. 1995;50(9):815–9. Available from: http://ovidsp.ovid.com/ovidweb.cgi?T=JS&PAGE=reference&D=med3&NEWS=N&AN=8587720

468. Fahrni GT, Vuille-Dit-Bille RN, Leu S, Meuli M, Staerkle RF, Fink L, et al. Five-year Follow-up and Recurrence Rates Following Surgery for Acute and Chronic Pilonidal Disease: A Survey of 421 Cases. Wounds a Compend Clin Res Pract [Internet]. 2016;28(1):20–6. Available from: http://ovidsp.ovid.com/ovidweb.cgi?T=JS&PAGE=reference&D=med13&NEWS=N&AN=26824973

469. Arer IM, Yabanoglu H, Caliskan K. Tension-free primary closure for the treatment of pilonidal disease. Ann Ital Chir [Internet]. 2015;86:459–63. Available from: http://ovidsp.ovid.com/ovidweb.cgi?T=JS&PAGE=reference&D=med12&NEWS=N&AN=26567718

470. Emir S, Topuz O, Kanat BH, Bali I. Sinotomy technique versus surgical excision with primary closure technique in pilonidal sinus disease. Bosn J basic Med Sci [Internet]. 2014;14(4):263–7. Available from: http://ovidsp.ovid.com/ovidweb.cgi?T=JS&PAGE=reference&D=med11&NEWS=N&AN=25428682

471. Varnalidis I, Ioannidis O, Paraskevas G, Papapostolou D, Malakozis SG, Gatzos S, et al. Pilonidal sinus: a comparative study of treatment methods. J Med Life [Internet]. 2014;7(1):27–30. Available from: http://ovidsp.ovid.com/ovidweb.cgi?T=JS&PAGE=reference&D=med11&NEWS=N&AN=24653753

472. Rabie ME, Al Refeidi AA, Al Haizaee A, Hilal S, Al Ajmi H, Al Amri AA. Sacrococcygeal pilonidal disease: sinotomy versus excisional surgery, a retrospective study. ANZ J Surg [Internet]. 2007;77(3):177–80. Available from: http://ovidsp.ovid.com/ovidweb.cgi?T=JS&PAGE=reference&D=med6&NEWS=N&AN=17305995

473. Bianco V, Basile C, Tortorella M. [Sacrococcygeal pilonidal sinus disease. Treatment by “open” and “closed” technique: personal experience]. La Mal pilonidale sacrococcigea Tratt con Metod “aperto” e “chiuso” Esper Pers [Internet]. 2003;24(4):145–7. Available from: http://ovidsp.ovid.com/ovidweb.cgi?T=JS&PAGE=reference&D=med5&NEWS=N&AN=12886754

474. Iesalnieks I, Furst A, Rentsch M, Jauch K-W. [Primary midline closure after excision of a pilonidal sinus is associated with a high recurrence rate]. Erhohtes Rezidivrisiko nach primarem Median Wundverschluss bei Patienten mit Pilonidalsinus [Internet]. 2003;74(5):461–8. Available from: http://ovidsp.ovid.com/ovidweb.cgi?T=JS&PAGE=reference&D=med5&NEWS=N&AN=12748795

475. Perruchoud C, Vuilleumier H, Givel JC. Pilonidal sinus: how to choose between excision and open granulation versus excision and primary closure? Study of a series of 141 patients operated on from 1991 to 1995. Swiss Surg [Internet]. 2002;8(6):255–8. Available from: http://ovidsp.ovid.com/ovidweb.cgi?T=JS&PAGE=reference&D=med4&NEWS=N&AN=12520844

476. Baier PK, Baumgartner U, Furtwangler A, Holzinger F, Schoffel U. [Therapy of the pilonidal sinus--Primary wound closure or open wound after excision]. Die Ther des Sinus pilonidalis - Primarer Wundverschluss versus offene Wundbehandlung nach Exzision [Internet]. 2002;127(4):310–4. Available from: http://ovidsp.ovid.com/ovidweb.cgi?T=JS&PAGE=reference&D=med4&NEWS=N&AN=12085282

477. Spivak H, Brooks VL, Nussbaum M, Friedman I. Treatment of chronic pilonidal disease. Dis Colon Rectum [Internet]. 1996;39(10):1136–9. Available from: http://ovidsp.ovid.com/ovidweb.cgi?T=JS&PAGE=reference&D=med4&NEWS=N&AN=8831530

478. Aaser P, Gruner OP. [Pilonidal cysts. Excision and intracutaneous absorbable primary suture]. Pilonidalcyste Ekstirpasjon og intrakutan resorberbar primaersutur [Internet]. 1992;112(2):206–7. Available from: http://ovidsp.ovid.com/ovidweb.cgi?T=JS&PAGE=reference&D=med3&NEWS=N&AN=1566250

479. Del Papa M, Mobili M, Attardo S, Paolucci G, Braccioni U. [Radical excision and primary suture of pilonidal sinus: our experience]. Escissione Radic e sutura primaria del sinus pilonidalis nostra esperienza [Internet]. 1992;63(4):507–10. Available from: http://ovidsp.ovid.com/ovidweb.cgi?T=JS&PAGE=reference&D=med3&NEWS=N&AN=1463266

480. Morell V, Charlton BL, Deshmukh N. Surgical treatment of pilonidal disease: comparison of three different methods in fifty-nine cases. Mil Med [Internet]. 1991;156(3):144–6. Available from: http://ovidsp.ovid.com/ovidweb.cgi?T=JS&PAGE=reference&D=med3&NEWS=N&AN=1901978

481. Cimarelli S, Magnano G. [Treatment of pilonidal sinus. Our experience]. Tratt del “sinus pilonidalis” Nostra esperienza [Internet]. 1989;44(7):1131–4. Available from: http://ovidsp.ovid.com/ovidweb.cgi?T=JS&PAGE=reference&D=med3&NEWS=N&AN=2747956

482. McLaren CA. Partial closure and other techniques in pilonidal surgery: an assessment of 157 cases. Br J Surg [Internet]. 1984;71(7):561–2. Available from: http://ovidsp.ovid.com/ovidweb.cgi?T=JS&PAGE=reference&D=med2&NEWS=N&AN=6733433

483. Doll D, Matevossian E, Luedi MM, Schneider R, van Zypen D, Novotny A. Does Full Wound Rupture following Median Pilonidal Closure Alter Long-Term Recurrence Rate?. Med Princ Pract [Internet]. 2015;24(6):571–7. Available from: http://ovidsp.ovid.com/ovidweb.cgi?T=JS&PAGE=reference&D=med12&NEWS=N&AN=26334688

484. Nasr A, Ein SH. A pediatric surgeon’s 35-year experience with pilonidal disease in a Canadian children’s hospital. Can J Surg [Internet]. 2011;54(1):39–42. Available from: http://ovidsp.ovid.com/ovidweb.cgi?T=JS&PAGE=reference&D=med8&NEWS=N&AN=21251431

485. Chiedozi LC, Al-Rayyes FA, Salem MM, Al-Haddi FH, Al-Bidewi AA. Management of pilonidal sinus. Saudi Med J [Internet]. 2002;23(7):786–8. Available from: http://ovidsp.ovid.com/ovidweb.cgi?T=JS&PAGE=reference&D=med4&NEWS=N&AN=12174225

486. Sequeira JB, Coelho A, Marinho AS, Bonet B, Carvalho F, Moreira-Pinto J. Endoscopic pilonidal sinus treatment versus total excision with primary closure for sacrococcygeal pilonidal sinus disease in the pediatric population. J Pediatr Surg [Internet]. 2018;53(10):2003–7. Available from: http://ovidsp.ovid.com/ovidweb.cgi?T=JS&PAGE=reference&D=med15&NEWS=N&AN=29602548

487. Mutus HM, Aksu B, Uzun E, Gulcin N, Gercel G, Ozatman E, et al. Long-term analysis of surgical treatment outcomes in chronic pilonidal sinus disease. J Pediatr Surg [Internet]. 2018;53(2):293–4. Available from: http://ovidsp.ovid.com/ovidweb.cgi?T=JS&PAGE=reference&D=med15&NEWS=N&AN=29217319

488. Muzi MG, Mascagni P, Buonomo O, Cianfarani A, Mosconi C, Colella M, et al. Muzi’s Tension Free Primary Closure of Pilonidal Sinus Disease: Updates on Long-Term Results on 514 Patients. J Gastrointest Surg [Internet]. 2018;22(1):133–7. Available from: http://ovidsp.ovid.com/ovidweb.cgi?T=JS&PAGE=reference&D=med15&NEWS=N&AN=28752401

489. Kravchenko SP. [THE WAYS TO IMPROVE SURGICAL TREATMENT OF PILONIDAL DISEASE]. Klin khirurhiia [Internet]. 2016;(2):11–4. Available from: http://ovidsp.ovid.com/ovidweb.cgi?T=JS&PAGE=reference&D=med13&NEWS=N&AN=27244909

490. Muzi MG, Maglio R, Milito G, Nigro C, Ciangola I, Bernagozzi B, et al. Long-term results of pilonidal sinus disease with modified primary closure: new technique on 450 patients. Am Surg [Internet]. 2014;80(5):484–8. Available from: http://ovidsp.ovid.com/ovidweb.cgi?T=JS&PAGE=reference&D=med11&NEWS=N&AN=24887728

491. Fitzpatrick EB, Chesley PM, Oguntoye MO, Maykel JA, Johnson EK, Steele SR. Pilonidal disease in a military population: how far have we really come?. Am J Surg [Internet]. 2014;207(6):907–14. Available from: http://ovidsp.ovid.com/ovidweb.cgi?T=JS&PAGE=reference&D=med11&NEWS=N&AN=24239526

492. Gonzalez-Temprano N, Sanchez-Vazquez M, Ayuso-Gonzalez L, Pison-Chacon J, Perez-Martinez A. [Are we correctly treating pilonidal disease in children? therapeutic goals beyond preventing recurrence]. Estamos tratando bien la Enferm pilonidal en los ninos? Objet Ter mas alla Prev la Recidiv [Internet]. 2011;24(3):161–4. Available from: http://ovidsp.ovid.com/ovidweb.cgi?T=JS&PAGE=reference&D=med8&NEWS=N&AN=22295658

493. Popeskou S, Christoforidis D, Ruffieux C, Demartines N. Wound infection after excision and primary midline closure for pilonidal disease: risk factor analysis to improve patient selection. World J Surg [Internet]. 2011;35(1):206–11. Available from: http://ovidsp.ovid.com/ovidweb.cgi?T=JS&PAGE=reference&D=med8&NEWS=N&AN=20931197

494. Gilani SNS, Furlong H, Reichardt K, Nasr AO, Theophilou G, Walsh TN. Excision and primary closure of pilonidal sinus disease: worthwhile option with an acceptable recurrence rate. Ir J Med Sci [Internet]. 2011;180(1):173–6. Available from: http://ovidsp.ovid.com/ovidweb.cgi?T=JS&PAGE=reference&D=med8&NEWS=N&AN=20661780

495. Gidwani AL, Murugan K, Nasir A, Brown R. Incise and lay open: an effective procedure for coccygeal pilonidal sinus disease. Ir J Med Sci [Internet]. 2010;179(2):207–10. Available from: http://ovidsp.ovid.com/ovidweb.cgi?T=JS&PAGE=reference&D=med8&NEWS=N&AN=20091138

496. Al-Khayat H, Al-Khayat H, Sadeq A, Groof A, Haider HH, Hayati H, et al. Risk factors for wound complication in pilonidal sinus procedures. J Am Coll Surg [Internet]. 2007;205(3):439–44. Available from: http://ovidsp.ovid.com/ovidweb.cgi?T=JS&PAGE=reference&D=med6&NEWS=N&AN=17765160

497. Rosato L, Fornero G, Luc AR, Clerico G. [The radical treatment of sacrococcygeal pilonidal cysts]. Tratt Radic della Cist pilonidale sacrococcigea [Internet]. 1997;52(10):1277–9. Available from: http://ovidsp.ovid.com/ovidweb.cgi?T=JS&PAGE=reference&D=med4&NEWS=N&AN=9471586

498. Solla JA, Rothenberger DA. Chronic pilonidal disease. An assessment of 150 cases. Dis Colon Rectum [Internet]. 1990;33(9):758–61. Available from: http://ovidsp.ovid.com/ovidweb.cgi?T=JS&PAGE=reference&D=med3&NEWS=N&AN=2390911

499. Ozcan R, Huseynov M, Bakir AC, Emre S, Tutuncu C, Celayir S, et al. Which treatment modality for pediatric pilonidal sinus: Primary repair or secondary healing?. Asian J Surg [Internet]. 2018;41(5):506–10. Available from: http://ovidsp.ovid.com/ovidweb.cgi?T=JS&PAGE=reference&D=med15&NEWS=N&AN=29042151

500. Toccaceli S, Persico Stella L, Diana M, Dandolo R, Negro P. Treatment of pilonidal sinus with primary closure. A twenty-year experience. Chir Ital [Internet]. 2008;60(3):433–8. Available from: http://ovidsp.ovid.com/ovidweb.cgi?T=JS&PAGE=reference&D=med7&NEWS=N&AN=18709783

501. Lavreshin PM, Gobedzhishvili VK, Aliev MO, Khutov AB, Murtazaev TS, Lavreshin MP. [Method of surgical treatment of the epithelial coccygeal track complicated by numerous fistulas of the sacrococcygeal area]. Vestn Khir Im I I Grek [Internet]. 2007;166(4):63–5. Available from: http://ovidsp.ovid.com/ovidweb.cgi?T=JS&PAGE=reference&D=med6&NEWS=N&AN=17966659

502. Akinci OF, Coskun A, Ozgonul A, Terzi A. Surgical treatment of complicated pilonidal disease: limited separate elliptical excision with primary closure. Colorectal Dis [Internet]. 2006;8(8):704–9. Available from: http://ovidsp.ovid.com/ovidweb.cgi?T=JS&PAGE=reference&D=med6&NEWS=N&AN=16970582

503. Ciccolo A, Rossitto M, Panacea D, Manfre A, Buonamonte S, Ardizzone A. Treatment of pilonidal disease in short-stay surgery: personal method. Ann Ital Chir [Internet]. 2004;75(5):603–5. Available from: http://ovidsp.ovid.com/ovidweb.cgi?T=JS&PAGE=reference&D=med5&NEWS=N&AN=15960353

504. Greenberg R, Kashtan H, Skornik Y, Werbin N. Treatment of pilonidal sinus disease using fibrin glue as a sealant. Tech Coloproctol [Internet]. 2004;8(2):95–8. Available from: http://ovidsp.ovid.com/ovidweb.cgi?T=JS&PAGE=reference&D=med5&NEWS=N&AN=15309645

505. Ommer A, Pitt C, Albrecht K, Marla B, Peitgen K, Walz MK. [Pilonidal Sinus -- Primary Closure also in Case of Abscess?]. Sinus pilonidalis -- primarer Verschluss auch beim Abszess? [Internet]. 2004;129(3):216–9. Available from: http://ovidsp.ovid.com/ovidweb.cgi?T=JS&PAGE=reference&D=med5&NEWS=N&AN=15237330

506. Benfatto G, Catania G, Altadonna V, Licari V, Tenaglia L, Giovinetto R, et al. Drainage useful in the excision and closure “per primam” of pilonidal sinus: technical notes. Chir Ital [Internet]. 2003;55(4):621–4. Available from: http://ovidsp.ovid.com/ovidweb.cgi?T=JS&PAGE=reference&D=med5&NEWS=N&AN=12938615

507. Serour F, Somekh E, Krutman B, Gorenstein A. Excision with primary closure and suction drainage for pilonidal sinus in adolescent patients. Pediatr Surg Int [Internet]. 2002;18(2–3):159–61. Available from: http://ovidsp.ovid.com/ovidweb.cgi?T=JS&PAGE=reference&D=med4&NEWS=N&AN=11956785

508. Tritapepe R, Di Padova C. Excision and primary closure of pilonidal sinus using a drain for antiseptic wound flushing. Am J Surg [Internet]. 2002;183(2):209–11. Available from: http://ovidsp.ovid.com/ovidweb.cgi?T=JS&PAGE=reference&D=med4&NEWS=N&AN=11918891

509. Tocchi A, Costa G, Lepre L, Liotta G, Mazzoni G, Agostini N, et al. [Ambulatory closed surgery for the treatment of pilonidal sinus]. Tratt Chir ambulatoriale del sinus pilonidalis con Tec chiusa [Internet]. 2001;22(8–9):303–7. Available from: http://ovidsp.ovid.com/ovidweb.cgi?T=JS&PAGE=reference&D=med4&NEWS=N&AN=11682968

510. Al-Jaberi TM. Excision and simple primary closure of chronic pilonidal sinus. Eur J Surg [Internet]. 2001;167(2):133–5. Available from: http://ovidsp.ovid.com/ovidweb.cgi?T=JS&PAGE=reference&D=med4&NEWS=N&AN=11266254

511. Berry DP, Harding KG, Stanton MR, Jasani B, Ehrlich HP. Human wound contraction: collagen organization, fibroblasts, and myofibroblasts. Plast Reconstr Surg [Internet]. 1998;102(1):124. Available from: http://ovidsp.ovid.com/ovidweb.cgi?T=JS&PAGE=reference&D=med4&NEWS=N&AN=9655417

512. Khaira HS, Brown JH. Excision and primary suture of pilonidal sinus. Ann R Coll Surg Engl [Internet]. 1995;77(4):242–4. Available from: http://ovidsp.ovid.com/ovidweb.cgi?T=JS&PAGE=reference&D=med3&NEWS=N&AN=7574311

513. Garg P, Menon GR, Gupta V. Laying open (deroofing) and curettage of sinus as treatment of pilonidal disease: a systematic review and meta-analysis. ANZ J Surg [Internet]. 2016;86(1–2):27–33. Available from: http://ovidsp.ovid.com/ovidweb.cgi?T=JS&PAGE=reference&D=med13&NEWS=N&AN=26612320

514. Calikoglu I, Gulpinar K, Oztuna D, Elhan AH, Dogru O, Akyol C, et al. Phenol Injection Versus Excision With Open Healing in Pilonidal Disease: A Prospective Randomized Trial. Dis Colon Rectum [Internet]. 2017;60(2):161–9. Available from: http://ovidsp.ovid.com/ovidweb.cgi?T=JS&PAGE=reference&D=med14&NEWS=N&AN=28059912

515. Furnee EJB, Davids PHP, Pronk A, Smakman N. Pit excision with phenolisation of the sinus tract versus radical excision in sacrococcygeal pilonidal sinus disease: study protocol for a single centre randomized controlled trial. Trials [Internet]. 2015;16:92. Available from: http://ovidsp.ovid.com/ovidweb.cgi?T=JS&PAGE=reference&D=med12&NEWS=N&AN=25872666

516. Parlakgumus A, Ezer A, Caliskan K, Emeksiz S, Karakaya J, Colakoglu T, et al. Effects of a tissue sealing-cutting device versus monopolar electrocautery on early pilonidal wound healing: a prospective randomized controlled trial. Dis Colon Rectum [Internet]. 2011;54(9):1155–61. Available from: http://ovidsp.ovid.com/ovidweb.cgi?T=JS&PAGE=reference&D=med8&NEWS=N&AN=21825897

517. Gupta PJ. Comparative study between radiofrequency sinus excision and open excision in sacro-coccygeal pilonidal sinus disease. Dig Surg [Internet]. 2005;22(6):459–63. Available from: http://ovidsp.ovid.com/ovidweb.cgi?T=JS&PAGE=reference&D=med6&NEWS=N&AN=16549926

518. Mohamed HA, Kadry I, Adly S. Comparison between three therapeutic modalities for non-complicated pilonidal sinus disease. Surgeon [Internet]. 2005;3(2):73–7. Available from: http://ovidsp.ovid.com/ovidweb.cgi?T=JS&PAGE=reference&D=med6&NEWS=N&AN=15861940

519. Armstrong JH, Barcia PJ. Pilonidal sinus disease. The conservative approach. Arch Surg [Internet]. 1994;129(9):914–9. Available from: http://ovidsp.ovid.com/ovidweb.cgi?T=JS&PAGE=reference&D=med3&NEWS=N&AN=8080372

520. Ortiz HH, Marti J, Sitges A. Pilonidal sinus: a claim for simple track incision. Dis Colon Rectum [Internet]. 1977;20(4):325–8. Available from: http://ovidsp.ovid.com/ovidweb.cgi?T=JS&PAGE=reference&D=med1&NEWS=N&AN=862493

521. Pronk AA, Smakman N, Furnee EJB. Short-term outcomes of radical excision vs. phenolisation of the sinus tract in primary sacrococcygeal pilonidal sinus disease: a randomized-controlled trial. Tech Coloproctol [Internet]. 2019;23(7):665–73. Available from: http://ovidsp.ovid.com/ovidweb.cgi?T=JS&PAGE=reference&D=medl&NEWS=N&AN=31278458

522. Edwards MH. Pilonidal sinus: a 5-year appraisal of the Millar-Lord treatment. Br J Surg [Internet]. 1977;64(12):867–8. Available from: http://ovidsp.ovid.com/ovidweb.cgi?T=JS&PAGE=reference&D=med1&NEWS=N&AN=588984

523. DeRosario JL, Khare U. Pilonidal disease--a surgical enigma. Can Med Assoc J [Internet]. 1965;93(24):1262–7. Available from: http://ovidsp.ovid.com/ovidweb.cgi?T=JS&PAGE=reference&D=med1&NEWS=N&AN=5839226

524. Levinson T, Sela T, Chencinski S, Derazne E, Tzur D, Elad H, et al. Pilonidal Sinus Disease: A 10-Year Review Reveals Occupational Risk Factors and the Superiority of the Minimal Surgery Trephine Technique. Mil Med [Internet]. 2016 Apr;181(4):389–94. Available from: http://ovidsp.ovid.com/ovidweb.cgi?T=JS&PAGE=reference&D=med13&NEWS=N&AN=27046187

525. L. D, F. P, G. G, G. D, L. S. Primary closure of decentred wounds in surgical treatment of pilonidal sinus. Chirurgia (Bucur) [Internet]. 1998;11(4):239–44. Available from: http://ovidsp.ovid.com/ovidweb.cgi?T=JS&PAGE=reference&D=emed6&NEWS=N&AN=29126822

526. Chinn BT. Outpatient management of pilonidal disease. Semin Colon Rectal Surg [Internet]. 2003 Dec;14(4):166–72. Available from: http://search.ebscohost.com/login.aspx?direct=true&db=cin20&AN=106665335&site=ehost-live

527. N.M. H, D. M, R. L, S. B, S. B. The use of human dermal allograft for the treatment of chronic pilonidal disease: Comparison to traditional surgical methods. Dis Colon Rectum [Internet]. 2018;61(5):e236--e237. Available from: http://ovidsp.ovid.com/ovidweb.cgi?T=JS&PAGE=reference&D=emed19&NEWS=N&AN=622082091

528. A. K, M.R. K, M. K. Delayed wound healing after excision of sacrococcygeal pilonidal disease: Is it curettage a solution? Color Dis [Internet]. 2016;18(Supplement 1):118. Available from: http://ovidsp.ovid.com/ovidweb.cgi?T=JS&PAGE=reference&D=emed17&NEWS=N&AN=615291126

529. P. G, A. J, A. AK. Laying open and curettage under local anesthesia (locula)-a minimally invasive procedure for pilonidal sinus: Pilonidal disease management needs a paradigm shift from more to less. Dis Colon Rectum [Internet]. 2019;62(6):e251--e252. Available from: http://ovidsp.ovid.com/ovidweb.cgi?T=JS&PAGE=reference&D=emexa&NEWS=N&AN=628086277

530. Stewart A, Melbourne G, Donoghue J, Mitten-Lewis S, Rogan F, Wyllie A, et al. A pilot study of wound healing following surgical excision of pilonidal sinus. Collegian [Internet]. 2004 Jul;11(3):24–8. Available from: http://search.ebscohost.com/login.aspx?direct=true&db=cin20&AN=106677550&site=ehost-live

531. Giuseppe F, Silvia DG, Patrizia R, Riccardo P, Antonio DS, Aldo RS, et al. Pilonidal sinus disease: Preliminary case-control study on heat-related wound dehiscence. Ann Med Surg [Internet]. 2019 Dec;48:144–9. Available from: http://www.elsevier.com/journals/annals-of-medicine-and-surgery/2049-0801

532. M. C, R. R, L. F, S. SC. Retrospective study on the use of negative pressure wound therapy in the treatment of pilonidal cysts (sinus pilonidalis) operated on using an open technique or complicated by dehiscence of the surgery site through sepsis. Acta Vulnologica [Internet]. 2016;14(1):24–39. Available from: http://www.minervamedica.it/en/getpdf/fGgfiBGU5Mx6ENCvX%252FRshtkDWkcrter5Q50HZr0CHWBCSG8Ajv%252FDQpRRvB2L1O6VEx61YjGn7e82ObXCcbe9xA%253D%253D/R45Y2016N01A0024.pdf

533. Lasheen’s needles for closure of wound of PNS and trocar port. Eur Surg Res [Internet]. 2014;52(3–4):140–1. Available from: http://ovidsp.ovid.com/ovidweb.cgi?T=JS&PAGE=reference&D=emed15&NEWS=N&AN=71493269

534. Shirah BH, Shirah HA. Factors affecting the outcome and duration of healing of the laid open wound for sacrococcygeal pilonidal sinus: A prospective cohort study of 472 patients. Wound Med [Internet]. 2017 Sep;18:52–6. Available from: http://www.elsevier.com/journals/wound-medicine/2213-9095

535. Pilonidal sinus. A follow up investigation of outpatients treated by simple incision (Danish). Ugeskr Laeger [Internet]. 1975;137(14):789–91. Available from: http://ovidsp.ovid.com/ovidweb.cgi?T=JS&PAGE=reference&D=emed2&NEWS=N&AN=6030821

536. S. B. Pilonidal sinus: Open surgical technique. Tech Coloproctol [Internet]. 2014;18(4):416–7. Available from: http://ovidsp.ovid.com/ovidweb.cgi?T=JS&PAGE=reference&D=emed15&NEWS=N&AN=71437572

537. J.P. G. Pilonidal disease treated by wide excision and controlled cicatrisation. A report on 73 patients. Lyon Chir [Internet]. 1996;92(4):292–5. Available from: http://ovidsp.ovid.com/ovidweb.cgi?T=JS&PAGE=reference&D=emed6&NEWS=N&AN=26305657

538. M. K, S. S, E. B, C. S. The most commonly prescribed antibiotics therapy for pilonidal abscess is not always the most inappropriate. Color Dis [Internet]. 2015;17(SUPPL. 2):98. Available from: http://ovidsp.ovid.com/ovidweb.cgi?T=JS&PAGE=reference&D=emed16&NEWS=N&AN=72057782

539. A. K, A. K, M.S. F, A. M. Outcome of excision and healing by secondary intention for sacrocoxigeal pilonidal sinus. Color Dis [Internet]. 2014;16(SUPPL. 3):99. Available from: http://ovidsp.ovid.com/ovidweb.cgi?T=JS&PAGE=reference&D=emed15&NEWS=N&AN=71655385

540. M.R. K, A. K, S. J. Risk factors affecting wound complications and recurrence after excision of sacrococcygeal pilonidal disease. Color Dis [Internet]. 2016;18(Supplement 1):118. Available from: http://ovidsp.ovid.com/ovidweb.cgi?T=JS&PAGE=reference&D=emed17&NEWS=N&AN=615291773

541. Delshad HR, Henry O, Mooney DP. Improving Resource Utilization and Outcomes Using a Minimally Invasive Pilonidal Protocol. J Pediatr Surg [Internet]. 2020 Jan;55(1):182–6. Available from: http://ovidsp.ovid.com/ovidweb.cgi?T=JS&PAGE=reference&D=mesx&NEWS=N&AN=31676078

542. Mueller X, Rothenbuehler JM, Frede KE. [Sacrococcygeal cysts. Is Lord Millar’s procedure an alternative to exeresis?]. Kyste sacro-coccygien L’operation Lord Millar Represent une Altern a l’excision? [Internet]. 1991;128(11):487–90. Available from: http://ovidsp.ovid.com/ovidweb.cgi?T=JS&PAGE=reference&D=med3&NEWS=N&AN=1761604

543. Bissett IP, Isbister WH. The management of patients with pilonidal disease--a comparative study. Aust N Z J Surg [Internet]. 1987;57(12):939–42. Available from: http://ovidsp.ovid.com/ovidweb.cgi?T=JS&PAGE=reference&D=med2&NEWS=N&AN=3439938

544. Doll D, Luedi MM, Evers T, Kauf P, Matevossian E. Recurrence-free survival, but not surgical therapy per se, determines 583 patients’ long-term satisfaction following primary pilonidal sinus surgery. Int J Colorectal Dis [Internet]. 2015;30(5):605–11. Available from: http://ovidsp.ovid.com/ovidweb.cgi?T=JS&PAGE=reference&D=med12&NEWS=N&AN=25687246

545. Lee SL, Tejirian T, Abbas MA. Current management of adolescent pilonidal disease. J Pediatr Surg [Internet]. 2008;43(6):1124–7. Available from: http://ovidsp.ovid.com/ovidweb.cgi?T=JS&PAGE=reference&D=med7&NEWS=N&AN=18558194

546. Shafik A. Electrocauterization in the treatment of pilonidal sinus. Int Surg [Internet]. 1996;81(1):83–4. Available from: http://ovidsp.ovid.com/ovidweb.cgi?T=JS&PAGE=reference&D=med4&NEWS=N&AN=8803713

547. Halleran DR, Lopez JJ, Lawrence AE, Sebastiao Y V, Fischer BA, Cooper JN, et al. Recurrence of Pilonidal Disease: Our Best is Not Good Enough. J Surg Res [Internet]. 2018;232:430–6. Available from: http://ovidsp.ovid.com/ovidweb.cgi?T=JS&PAGE=reference&D=med15&NEWS=N&AN=30463753

548. Burney RE. Treatment of pilonidal disease by minimal surgical excision under local anesthesia with healing by secondary intention: Results in over 500 patients. Surgery [Internet]. 2018;164(6):1217–22. Available from: http://ovidsp.ovid.com/ovidweb.cgi?T=JS&PAGE=reference&D=med15&NEWS=N&AN=30064734

549. Tavangari FR, Lee JA, Garza D, Tejirian T. Outcomes of Unroofing with Limited Excision and Structured Postoperative Care for Pilonidal Disease. Am Surg [Internet]. 2017;83(10):1045–9. Available from: http://ovidsp.ovid.com/ovidweb.cgi?T=JS&PAGE=reference&D=med14&NEWS=N&AN=29391092

550. Speter C, Zmora O, Nadler R, Shinhar D, Bilik R. Minimal incision as a promising technique for resection of pilonidal sinus in children. J Pediatr Surg [Internet]. 2017;52(9):1484–7. Available from: http://ovidsp.ovid.com/ovidweb.cgi?T=JS&PAGE=reference&D=med14&NEWS=N&AN=28366559

551. Alptekin H, Yilmaz H, Kayis SA, Sahin M. Volume of the excised specimen and prediction of surgical site infection in pilonidal sinus procedures (surgical site infection after pilonidal sinus surgery). Surg Today [Internet]. 2013;43(12):1365–70. Available from: http://ovidsp.ovid.com/ovidweb.cgi?T=JS&PAGE=reference&D=med10&NEWS=N&AN=23224334

552. Tejirian T, Lee JJ, Abbas MA. Is wide local excision for pilonidal disease still justified?. Am Surg [Internet]. 2007;73(10):1075–8. Available from: http://ovidsp.ovid.com/ovidweb.cgi?T=JS&PAGE=reference&D=med6&NEWS=N&AN=17983085

553. Al-Homoud SJ, Habib ZS, Abdul Jabbar AS, Isbister WH. Management of sacrococcygeal pilonidal disease. Saudi Med J [Internet]. 2001;22(9):762–4. Available from: http://ovidsp.ovid.com/ovidweb.cgi?T=JS&PAGE=reference&D=med4&NEWS=N&AN=11590447

554. Anyanwu AC, Hossain S, Williams A, Montgomery AC. Karydakis operation for sacrococcygeal pilonidal sinus disease: experience in a district general hospital. Ann R Coll Surg Engl [Internet]. 1998;80(3):197–9. Available from: http://ovidsp.ovid.com/ovidweb.cgi?T=JS&PAGE=reference&D=med4&NEWS=N&AN=9682644

555. Nahas SC, Sobrado Junior CW, Araujo SE, Imperiale AR, Habr-Gama A, Pinotti HW. [Results of the surgical treatment of non-complicated pilonidal disease]. Result do Trat Cir Cist pilonidal nao-complicado [Internet]. 1997;52(6):287–90. Available from: http://ovidsp.ovid.com/ovidweb.cgi?T=JS&PAGE=reference&D=med4&NEWS=N&AN=9629736

556. Menzel T, Dorner A, Cramer J. [Excision and open wound treatment of pilonidal sinus. Rate of recurrence and duration of work incapacity]. Exzision und offene Wundbehandlung des Sinus pilonidalis Rezidivrate und Dauer der Arbeitsunfahigkeit [Internet]. 1997;122(47):1447–51. Available from: http://ovidsp.ovid.com/ovidweb.cgi?T=JS&PAGE=reference&D=med4&NEWS=N&AN=9424422

557. Papp F, Vecsei F. [Retrospective study 10 years after plastic surgery for sacrococcygeal pilonidal sinus]. Sacrococcygealis sinus pilonidalis miatt Vegz plasztikai eljarasunk Eredm 10 eves utanvizsgalata [Internet]. 1995;136(2):71–4. Available from: http://ovidsp.ovid.com/ovidweb.cgi?T=JS&PAGE=reference&D=med3&NEWS=N&AN=7862434

558. Palmieri B, Gozzi G, Rossi A. [Pilonidal cysts: the state of the art and the authors’ personal experiences]. Cist pilonidali stato dell’arte ed esperienze Pers [Internet]. 1994;49(5):377–82. Available from: http://ovidsp.ovid.com/ovidweb.cgi?T=JS&PAGE=reference&D=med3&NEWS=N&AN=7970033

559. Webb PM, Wysocki AP. Does pilonidal abscess heal quicker with off-midline incision and drainage?. Tech Coloproctol [Internet]. 2011;15(2):179–83. Available from: http://ovidsp.ovid.com/ovidweb.cgi?T=JS&PAGE=reference&D=med8&NEWS=N&AN=21533783

560. Petrovic J, Dimitrijevic I, Krivokapic Z. Minor vs complete excision of pilonidal sinus--early postoperative period. Acta Chir Iugosl [Internet]. 2012;59(2):81–5. Available from: http://ovidsp.ovid.com/ovidweb.cgi?T=JS&PAGE=reference&D=med9&NEWS=N&AN=23373363

561. Spyridakis M, Christodoulidis G, Chatzitheofilou C, Symeonidis D, Tepetes K. The role of the platelet-rich plasma in accelerating the wound-healing process and recovery in patients being operated for pilonidal sinus disease: preliminary results. World J Surg [Internet]. 2009;33(8):1764–9. Available from: http://ovidsp.ovid.com/ovidweb.cgi?T=JS&PAGE=reference&D=med7&NEWS=N&AN=19424751

562. Marzouk DM, Abou-Zeid AA, Antoniou A, Haji A, Benziger H. Sinus excision, release of coccycutaneous attachments and dermal-subcuticular closure (XRD procedure): a novel technique in flattening the natal cleft in pilonidal sinus treatment. Ann R Coll Surg Engl [Internet]. 2008;90(5):371–6. Available from: http://ovidsp.ovid.com/ovidweb.cgi?T=JS&PAGE=reference&D=med7&NEWS=N&AN=18634729

563. Blanco G, Giordano M, Torelli I. [Surgical treatment of pilonidal sinus with open surgical technique]. Tratt Chir del sinus pilonidalis col Metod aperto [Internet]. 2003;58(2):181–7. Available from: http://ovidsp.ovid.com/ovidweb.cgi?T=JS&PAGE=reference&D=med5&NEWS=N&AN=12738928

564. Soll C, Hahnloser D, Dindo D, Clavien P-A, Hetzer F. A novel approach for treatment of sacrococcygeal pilonidal sinus: less is more. Int J Colorectal Dis [Internet]. 2008;23(2):177–80. Available from: http://ovidsp.ovid.com/ovidweb.cgi?T=JS&PAGE=reference&D=med7&NEWS=N&AN=17703314

565. Aldean I, Shankar PJ, Mathew J, Safarani N, Haboubi NY. Simple excision and primary closure of pilonidal sinus: a simple modification of conventional technique with excellent results. Colorectal Dis [Internet]. 2005;7(1):81–5. Available from: http://ovidsp.ovid.com/ovidweb.cgi?T=JS&PAGE=reference&D=med6&NEWS=N&AN=15606592

566. Isbister WH, Prasad J. Pilonidal disease. Aust N Z J Surg [Internet]. 1995;65(8):561–3. Available from: http://ovidsp.ovid.com/ovidweb.cgi?T=JS&PAGE=reference&D=med3&NEWS=N&AN=7661795

567. Arseniuk V V, Bartosh AN, Gorban’ VR, Mazurik E V, Nosko VA, Petrovskii I V. [The programmed revision of the wound after the radical excision of an epithelial coccygeal cyst]. Program Reviz rany posle radikal’nogo issecheniia Ep kopchikovogo khoda [Internet]. 1993;(1):27–8. Available from: http://ovidsp.ovid.com/ovidweb.cgi?T=JS&PAGE=reference&D=med3&NEWS=N&AN=10912024

568. Muller XM, Rothenbuhler JM, Frede KE. [Sacro-coccygeal cyst: surgical techniques and results]. Kyste sacro-coccygien Tech Oper Result [Internet]. 1992;58(6):889–92. Available from: http://ovidsp.ovid.com/ovidweb.cgi?T=JS&PAGE=reference&D=med3&NEWS=N&AN=1644610

569. Nazarov LU, Ambartsumian RA, Akopian EB. [Treatment of pustular lesions of the perineum and sacrococcygeal region]. Lechenie gnoinikovykh porazhenii promezhnosti i kresttsovo- kopchikovoi Obl [Internet]. 1990;(6):79–82. Available from: http://ovidsp.ovid.com/ovidweb.cgi?T=JS&PAGE=reference&D=med3&NEWS=N&AN=2214582

570. Jensen SL, Harling H. Prognosis after simple incision and drainage for a first-episode acute pilonidal abscess. Br J Surg [Internet]. 1988;75(1):60–1. Available from: http://ovidsp.ovid.com/ovidweb.cgi?T=JS&PAGE=reference&D=med3&NEWS=N&AN=3337954

571. Obeid SA. A new technique for treatment of pilonidal sinus. Dis Colon Rectum [Internet]. 1988;31(11):879–85. Available from: http://ovidsp.ovid.com/ovidweb.cgi?T=JS&PAGE=reference&D=med3&NEWS=N&AN=3053071

572. Marks J, Harding KG, Hughes LE, Ribeiro CD. Pilonidal sinus excision--healing by open granulation. Br J Surg [Internet]. 1985;72(8):637–40. Available from: http://ovidsp.ovid.com/ovidweb.cgi?T=JS&PAGE=reference&D=med2&NEWS=N&AN=4027539

573. Houston HE. One-stage cure of infected pilonidal cysts. Am Surg [Internet]. 1977;43(8):517–9. Available from: http://ovidsp.ovid.com/ovidweb.cgi?T=JS&PAGE=reference&D=med1&NEWS=N&AN=889188

574. Eftaiha M, Abcarian H. The dilemma of pilonidal disease: surgical treatment. Dis Colon Rectum [Internet]. 1977;20(4):279–86. Available from: http://ovidsp.ovid.com/ovidweb.cgi?T=JS&PAGE=reference&D=med1&NEWS=N&AN=862486

575. Yi G, Soyele B, Bempah M, Valdes L, Trang J, Miller A, et al. Long-term outcomes comparing conventional surgical management versus new minimally invasive techniques of pilonidal sinus disease. Meta-analysis and review of literature. Surg Obes Relat Dis [Internet]. 2018 Nov;14(11):S168–9. Available from: http://ovidsp.ovid.com/ovidweb.cgi?T=JS&PAGE=reference&D=emed19&NEWS=N&AN=2001234576

576. Oncel M, Kurt N, Kement M, Colak E, Eser M, Uzun H. Excision and marsupialization versus sinus excision for the treatment of limited chronic pilonidal disease: a prospective, randomized trial. Tech Coloproctol [Internet]. 2002;6(3):165–9. Available from: http://ovidsp.ovid.com/ovidweb.cgi?T=JS&PAGE=reference&D=med4&NEWS=N&AN=12525910

577. Demir U, Yazici P, Bostanci O, Kaya C, Isil RG, T Mihmanli M. Less is more: “incision and curettage” as an optimal procedure for recurrent pilonidal disease. Ann Ital Chir [Internet]. 2015;86:575–9. Available from: http://ovidsp.ovid.com/ovidweb.cgi?T=JS&PAGE=reference&D=med12&NEWS=N&AN=26900142

578. Milone M, Velotti N, Manigrasso M, Milone F, Sosa Fernandez LM, De Palma GD. Video-assisted ablation of pilonidal sinus (VAAPS) versus sinusectomy for treatment of chronic pilonidal sinus disease: a comparative study. Updates Surg [Internet]. 2019;71(1):179–83. Available from: http://ovidsp.ovid.com/ovidweb.cgi?T=JS&PAGE=reference&D=med16&NEWS=N&AN=30542957

579. Javed MA, Fowler H, Jain Y, Singh S, Scott M, Rajaganeshan R. Comparison of conventional incision and drainage for pilonidal abscess versus novel endoscopic pilonidal abscess treatment (EPAT). Tech Coloproctol [Internet]. 2016;20(12):871–3. Available from: http://ovidsp.ovid.com/ovidweb.cgi?T=JS&PAGE=reference&D=med13&NEWS=N&AN=27888441

580. Iesalnieks I, Deimel S, Kienle K, Schlitt HJ, Zulke C. [Pit-picking surgery for pilonidal disease]. Pit-picking-Operation bei Patienten mit Sinus pilonidalis [Internet]. 2011;82(10):927–31. Available from: http://ovidsp.ovid.com/ovidweb.cgi?T=JS&PAGE=reference&D=med8&NEWS=N&AN=21424286

581. Eryilmaz R, Sahin M, Alimoglu O, Kaya B. [The comparison of incision and drainage with skin excision and curettage in the treatment of acute pilonidal abscess]. Akut pilonidal apse tedavisinde insizyon ve drenajin cilt eksizyonu ve kuretajla karsilastirilmasi [Internet]. 2003;9(2):120–3. Available from: http://ovidsp.ovid.com/ovidweb.cgi?T=JS&PAGE=reference&D=med5&NEWS=N&AN=12836108

582. Di Castro A, Guerra F, Levi Sandri GB, Ettorre GM. Minimally invasive surgery for the treatment of pilonidal disease. The Gips procedure on 2347 patients. Int J Surg [Internet]. 2016;36(Pt A):201–5. Available from: http://ovidsp.ovid.com/ovidweb.cgi?T=JS&PAGE=reference&D=med13&NEWS=N&AN=27989917

583. Qayyum I, Bai D, Tsoraides SS. Loop drainage after debridement (LDAD): minimally invasive treatment for pilonidal cyst. Tech Coloproctol [Internet]. 2016;20(8):591–4. Available from: http://ovidsp.ovid.com/ovidweb.cgi?T=JS&PAGE=reference&D=med13&NEWS=N&AN=27142855

584. Soll C, Dindo D, Steinemann D, Hauffe T, Clavien P-A, Hahnloser D. Sinusectomy for primary pilonidal sinus: less is more. Surgery [Internet]. 2011;150(5):996–1001. Available from: http://ovidsp.ovid.com/ovidweb.cgi?T=JS&PAGE=reference&D=med8&NEWS=N&AN=21911239

585. Kement M, Oncel M, Kurt N, Kaptanoglu L. Sinus excision for the treatment of limited chronic pilonidal disease: results after a medium-term follow-up. Dis Colon Rectum [Internet]. 2006;49(11):1758–62. Available from: http://ovidsp.ovid.com/ovidweb.cgi?T=JS&PAGE=reference&D=med6&NEWS=N&AN=16990977

586. Golladay ES, Wagner CW. Pediatric pilonidal disease: a method of management. South Med J [Internet]. 1990;83(8):922–4. Available from: http://ovidsp.ovid.com/ovidweb.cgi?T=JS&PAGE=reference&D=med3&NEWS=N&AN=2382157

587. Iesalnieks I, Deimel S, Schlitt HJ. ["Pit picking" surgery for patients with pilonidal disease : mid-term results and risk factors]. “Pit-picking”-Operation bei Patienten mit Sinus pilonidalis Mittelfristige Ergebnisse und Risikofaktoren [Internet]. 2015;86(5):482–5. Available from: http://ovidsp.ovid.com/ovidweb.cgi?T=JS&PAGE=reference&D=med12&NEWS=N&AN=24969346

588. Delshad HR, Dawson M, Melvin P, Zotto S, Mooney DP. Pit-picking resolves pilonidal disease in adolescents. J Pediatr Surg [Internet]. 2019;54(1):174–6. Available from: http://ovidsp.ovid.com/ovidweb.cgi?T=JS&PAGE=reference&D=med16&NEWS=N&AN=30661599

589. Lopez JJ, Cooper JN, Halleran DR, Deans KJ, Minneci PC. High Rate of Major Morbidity after Surgical Excision for Pilonidal Disease. Surg Infect (Larchmt) [Internet]. 2018;19(6):603–7. Available from: http://ovidsp.ovid.com/ovidweb.cgi?T=JS&PAGE=reference&D=med15&NEWS=N&AN=29870307

590. Yamashita Y, Nagae H, Hashimoto I. Ambulatory Surgery for Pilonidal Sinus: Tract Excision and Open Treatment Followed by At-Home Irrigation. J Med Invest [Internet]. 2016;63(3–4):216–8. Available from: http://ovidsp.ovid.com/ovidweb.cgi?T=JS&PAGE=reference&D=med13&NEWS=N&AN=27644561

591. Yalcin S, Ergul E. A single-surgeon, single-institute experience of 59 sinotomies for sacrococcygeal pilonidal disease under local anesthesia. Bratisl Lek Listy [Internet]. 2010;111(5):284–5. Available from: http://ovidsp.ovid.com/ovidweb.cgi?T=JS&PAGE=reference&D=med8&NEWS=N&AN=20568419

592. Kepenekci I, Demirkan A, Celasin H, Gecim IE. Unroofing and curettage for the treatment of acute and chronic pilonidal disease. World J Surg [Internet]. 2010;34(1):153–7. Available from: http://ovidsp.ovid.com/ovidweb.cgi?T=JS&PAGE=reference&D=med8&NEWS=N&AN=19820992

593. Gips M, Melki Y, Salem L, Weil R, Sulkes J. Minimal surgery for pilonidal disease using trephines: description of a new technique and long-term outcomes in 1,358 patients. Dis Colon Rectum [Internet]. 2008;51(11):1653–6. Available from: http://ovidsp.ovid.com/ovidweb.cgi?T=JS&PAGE=reference&D=med7&NEWS=N&AN=18516645

594. Weinstein MA, Rubin RJ, Salvati EP. The dilemma of pilonidal disease: pilonidal cystotomy, reappraisal of an old technique. Dis Colon Rectum [Internet]. 1977;20(4):287–9. Available from: http://ovidsp.ovid.com/ovidweb.cgi?T=JS&PAGE=reference&D=med1&NEWS=N&AN=862487

595. LORD PH, MILLAR DM. PILONIDAL SINUS: A SIMPLE TREATMENT. Br J Surg [Internet]. 1965;52:298–300. Available from: http://ovidsp.ovid.com/ovidweb.cgi?T=JS&PAGE=reference&D=med1&NEWS=N&AN=14271092

596. Neola B, Capasso S, Caruso L, Falato A, Ferulano GP. Scarless outpatient ablation of pilonidal sinus: a pilot study of a new minimally invasive treatment. Int Wound J [Internet]. 2016;13(5):705–8. Available from: http://ovidsp.ovid.com/ovidweb.cgi?T=JS&PAGE=reference&D=med13&NEWS=N&AN=25132617

597. A. W, C. K. Micronutrition deficits after pit picking with adjunct laser therapy in Pilonidal Cyst: A possible cause of failure. Color Dis [Internet]. 2018;20(Supplement 4):139–40. Available from: http://ovidsp.ovid.com/ovidweb.cgi?T=JS&PAGE=reference&D=emed19&NEWS=N&AN=624185962

598. Guerra F, Cirullo E, Di Castro A. Minimally Invasive Pilonidal Excision: Preliminary Report. World J Surg [Internet]. 2020 Apr 9;44(4):1086–90. Available from: http://ovidsp.ovid.com/ovidweb.cgi?T=JS&PAGE=reference&D=emexb&NEWS=N&AN=630140779

599. Panigrahi H, Rana RK, Rao M. KSHARASUTRA THERAPY - A MINIMAL INVASIVE PARASURGICAL METHOD IN THE TREATMENT OF SACROCOCCYGEAL PILONIDAL SINUS (NADI VRANA): RESULT OF A PILOT STUDY. Int J Res Ayurveda Pharm [Internet]. 2012 Oct 12;3(5):668–70. Available from: http://www.ijrap.net/admin/php/uploads/874_pdf.pdf

600. A. T, I. K. Sinusectomy results in treatment of pilonidal sinus. Color Dis [Internet]. 2014;16(SUPPL. 3):95–6. Available from: http://ovidsp.ovid.com/ovidweb.cgi?T=JS&PAGE=reference&D=emed15&NEWS=N&AN=71655366

601. Rogers P, Platell C, Levitt M. Minimal tissue excision in the treatment of pilonidal sinus disease: results from a single surgical unit. ANZ J Surg [Internet]. 2020 Apr 12;90(4):529–32. Available from: http://ovidsp.ovid.com/ovidweb.cgi?T=JS&PAGE=reference&D=emexb&NEWS=N&AN=630597816

602. M. N, W. M, M. I, R. W. Pilonidal sinus excision under local anesthesia: A day case experience. J Med Sci [Internet]. 2020;28(1):21–4. Available from: https://www.jmedsci.com/index.php/Jmedsci/article/download/879/627

603. A. S, A. T, N. K, M. Y, S. C, O.F. T. The results of phenol application in sinus pilonidalis treatment. Gulhane Med J [Internet]. 1999;41(1):51–6. Available from: http://www.gulhanemedicaljournal.org

604. A. G, O. S. Endoscopic pilonidal sinus treatment, a minimally invasive approach. Surg Endosc Other Interv Tech [Internet]. 2017;31(2 Supplement 1):S84. Available from: http://ovidsp.ovid.com/ovidweb.cgi?T=JS&PAGE=reference&D=emed18&NEWS=N&AN=617039306

605. I. I. PIT picking surgery for pilonidal disease: Mid-term results. Dis Colon Rectum [Internet]. 2014;57(5):e151--e152. Available from: http://ovidsp.ovid.com/ovidweb.cgi?T=JS&PAGE=reference&D=emed15&NEWS=N&AN=71493676

606. C. S, D. S, D. D, P.-A. C. Long-term results of limited excision for pilonidal sinus: Less is more. Color Dis [Internet]. 2010;12(SUPPL. 3):5. Available from: http://ovidsp.ovid.com/ovidweb.cgi?T=JS&PAGE=reference&D=emed11&NEWS=N&AN=70324986

607. A. DC. Minimally invasive pilonidal excision. Color Dis [Internet]. 2019;21(Supplement 3):116. Available from: http://ovidsp.ovid.com/ovidweb.cgi?T=JS&PAGE=reference&D=emexb&NEWS=N&AN=631604265

608. L. A-O, A. A-A, W. H, H. S, F. B. Modified Lord Miller procedure: Effective day case surgery for pilonidal sinus. Color Dis [Internet]. 2016;18(Supplement 1):113. Available from: http://ovidsp.ovid.com/ovidweb.cgi?T=JS&PAGE=reference&D=emed17&NEWS=N&AN=615291489

609. I. I, H.J. S. Posters. Color Dis [Internet]. 2010 Aug 12;12(SUPPL. 3):28–52. Available from: http://ovidsp.ovid.com/ovidweb.cgi?T=JS&PAGE=reference&D=emed11&NEWS=N&AN=70325314

610. B. A, T. A. Comparison of three methods in surgical treatment of pilonidal disease. Color Dis [Internet]. 2015;17(SUPPL. 2):89. Available from: http://ovidsp.ovid.com/ovidweb.cgi?T=JS&PAGE=reference&D=emed16&NEWS=N&AN=72057726

611. A. B, L. PS, R. S, G. O, E. L. Minimally invasive treatment of pilonidal disease whith the PIT picking technique. Tech Coloproctol [Internet]. 2020;24(4):386. Available from: http://ovidsp.ovid.com/ovidweb.cgi?T=JS&PAGE=reference&D=emexb&NEWS=N&AN=632060789

612. Pit Picking with additional laser treatment is superior to Pit Picking alone in PNS. Color Dis [Internet]. 2015;17(SUPPL. 2):100. Available from: http://ovidsp.ovid.com/ovidweb.cgi?T=JS&PAGE=reference&D=emed16&NEWS=N&AN=72057792

613. “SiLaC” - Pilonidal Sinus Laser Closure, preliminary results of a new procedure to treat sacro-coccygeal pilonidal cysts. Color Dis [Internet]. 2016;18(Supplement 1):39. Available from: http://ovidsp.ovid.com/ovidweb.cgi?T=JS&PAGE=reference&D=emed17&NEWS=N&AN=615291400

614. F. P, C. H, K. W. Pit Picking is a safe and successful therapeutic option in non-acute Pilonidal Sinus Disease. Color Dis [Internet]. 2016;18(Supplement 1):120. Available from: http://ovidsp.ovid.com/ovidweb.cgi?T=JS&PAGE=reference&D=emed17&NEWS=N&AN=615291335

615. F. P, C. H. Pit Picking is a safe and successful therapeutic option in non-acute Pilonidal Sinus disease. Color Dis [Internet]. 2018;20(Supplement 4):133. Available from: http://ovidsp.ovid.com/ovidweb.cgi?T=JS&PAGE=reference&D=emed19&NEWS=N&AN=624186141

616. A. D, D. T, J. R-Y, G. Z, K. S, M. E-K, et al. Sinusectomy for primary pilonidal sinus, a single centre’s ten years’ experience. Color Dis [Internet]. 2016;18(Supplement 1):15. Available from: http://ovidsp.ovid.com/ovidweb.cgi?T=JS&PAGE=reference&D=emed17&NEWS=N&AN=615291782

617. Gupta PJ. Radiofrequency sinus excision: better alternative to marsupialization technique in sacrococcygeal pilonidal sinus disease. J Natl Med Assoc [Internet]. 2005;97(7):998–1002. Available from: http://ovidsp.ovid.com/ovidweb.cgi?T=JS&PAGE=reference&D=med6&NEWS=N&AN=16080670

618. Gupta PJ. A randomized study between excision and marsupialization and radiofrequency sinus excision in sacro-coccygeal pilonidal disease. Curr Surg [Internet]. 2004;61(3):307–12. Available from: http://ovidsp.ovid.com/ovidweb.cgi?T=JS&PAGE=reference&D=med5&NEWS=N&AN=15165772

619. Rouch JD, Keeley JA, Scott A, Sydorak R, DeUgarte D, Lee SL. Short- and Long-term Results of Unroofing and Marsupialization for Adolescent Pilonidal Disease. JAMA Surg [Internet]. 2016;151(9):877–9. Available from: http://ovidsp.ovid.com/ovidweb.cgi?T=JS&PAGE=reference&D=med13&NEWS=N&AN=27224857

620. Smith CM, Jones A, Dass D, Murthi G, Lindley R. Early experience of the use of fibrin sealant in the management of children with pilonidal sinus disease. J Pediatr Surg [Internet]. 2015;50(2):320–2. Available from: http://ovidsp.ovid.com/ovidweb.cgi?T=JS&PAGE=reference&D=med12&NEWS=N&AN=25638628

621. Licheri S, Pisano G, Erdas E, Farci S, Pomata M, Daniele GM. Radical treatment of acute pilonidal abscess by marsupialization. G Chir [Internet]. 2004;25(11–12):414–6. Available from: http://ovidsp.ovid.com/ovidweb.cgi?T=JS&PAGE=reference&D=med5&NEWS=N&AN=15803819

622. Meban S, Hunter E. Outpatient treatment of pilonidal disease. Can Med Assoc J [Internet]. 1982;126(8):941. Available from: http://ovidsp.ovid.com/ovidweb.cgi?T=JS&PAGE=reference&D=med2&NEWS=N&AN=7074492

623. ABRAMSON DJ. A simple marsupialization technic for treatment of pilonidal sinus: long-term follow up. Ann Surg [Internet]. 1960;151:261–7. Available from: http://ovidsp.ovid.com/ovidweb.cgi?T=JS&PAGE=reference&D=med1&NEWS=N&AN=13791573

624. WATTERS N, MACDONALD IB. Marsupialization of pilonidal sinus and abscess: a report of 50 cases. Can Med Assoc J [Internet]. 1958;79(4):236–40. Available from: http://ovidsp.ovid.com/ovidweb.cgi?T=JS&PAGE=reference&D=med1&NEWS=N&AN=13573266

625. V. A, V. S, S. S, I. K, V. I, I. U. The using of electric welding in the minimally invasive treatment of the pilonidal disease. Color Dis [Internet]. 2018;20(Supplement 4):129. Available from: http://ovidsp.ovid.com/ovidweb.cgi?T=JS&PAGE=reference&D=emed19&NEWS=N&AN=624185834

626. P. G, V. T. Comparative study between two operative techniques of pilonidal sinus disease. Dis Colon Rectum [Internet]. 2013;56(4):e157. Available from: http://ovidsp.ovid.com/ovidweb.cgi?T=JS&PAGE=reference&D=emed14&NEWS=N&AN=71045602

627. F. Y, O. K. The comparison of marsupialization and Limberg flap in the treatment of pilonidal disease. Turkish J Surg [Internet]. 2005;21(4):184–90. Available from: http://www.turkjsurg.com/

628. Milone M, Di Minno MND, Musella M, Maietta P, Ambrosino P, Pisapia A, et al. The role of drainage after excision and primary closure of pilonidal sinus: a meta-analysis. Tech Coloproctol [Internet]. 2013;17(6):625–30. Available from: http://ovidsp.ovid.com/ovidweb.cgi?T=JS&PAGE=reference&D=med10&NEWS=N&AN=23754346

629. Milone M, Musella M, Salvatore G, Leongito M, Milone F. Effectiveness of a drain in surgical treatment of sacrococcygeal pilonidal disease. Results of a randomized and controlled clinical trial on 803 consecutive patients. Int J Colorectal Dis [Internet]. 2011;26(12):1601–7. Available from: http://ovidsp.ovid.com/ovidweb.cgi?T=JS&PAGE=reference&D=med8&NEWS=N&AN=21573899

630. Vahedian J, Nabavizadeh F, Nakhaee N, Vahedian M, Sadeghpour A. Comparison between drainage and curettage in the treatment of acute pilonidal abscess. Saudi Med J [Internet]. 2005;26(4):553–5. Available from: http://ovidsp.ovid.com/ovidweb.cgi?T=JS&PAGE=reference&D=med6&NEWS=N&AN=15900358

631. Tien T, Athem R, Arulampalam T. Outcomes of endoscopic pilonidal sinus treatment (EPSiT): a systematic review. Tech Coloproctol [Internet]. 2018;22(5):325–31. Available from: http://ovidsp.ovid.com/ovidweb.cgi?T=JS&PAGE=reference&D=med15&NEWS=N&AN=29850944

632. Emile SH, Elfeki H, Shalaby M, Sakr A, Giaccaglia V, Sileri P, et al. Endoscopic pilonidal sinus treatment: a systematic review and meta-analysis. Surg Endosc [Internet]. 2018;32(9):3754–62. Available from: http://ovidsp.ovid.com/ovidweb.cgi?T=JS&PAGE=reference&D=med15&NEWS=N&AN=29603009

633. H. R, R. K. Minimally invasive techniques in the management of pilonidal disease. Color Dis [Internet]. 2019;21(Supplement 2):35–6. Available from: http://ovidsp.ovid.com/ovidweb.cgi?T=JS&PAGE=reference&D=emexb&NEWS=N&AN=631603474

634. Milone M, Fernandez LMS, Musella M, Milone F. Safety and Efficacy of Minimally Invasive Video-Assisted Ablation of Pilonidal Sinus: A Randomized Clinical Trial. JAMA Surg [Internet]. 2016;151(6):547–53. Available from: http://ovidsp.ovid.com/ovidweb.cgi?T=JS&PAGE=reference&D=med13&NEWS=N&AN=26819186

635. Kalaiselvan R, Liyanage A, Rajaganeshan R. Short-term outcomes of endoscopic pilonidal sinus treatment. Ann R Coll Surg Engl [Internet]. 2020;102(2):94–7. Available from: http://ovidsp.ovid.com/ovidweb.cgi?T=JS&PAGE=reference&D=medl&NEWS=N&AN=31379189

636. Khafagy A, Al Haddad E, AlSabah S. The endoscopic treatment of pilonidal sinus disease: a short-term case-series study. Ann Saudi Med [Internet]. 2019;39(3):192–6. Available from: http://ovidsp.ovid.com/ovidweb.cgi?T=JS&PAGE=reference&D=medl&NEWS=N&AN=31215233

637. Giarratano G, Toscana C, Shalaby M, Buonomo O, Petrella G, Sileri P. Endoscopic Pilonidal Sinus Treatment: Long-Term Results of a Prospective Series. JSLS J Soc Laparoendosc Surg [Internet]. 2017;21(3). Available from: http://ovidsp.ovid.com/ovidweb.cgi?T=JS&PAGE=reference&D=med14&NEWS=N&AN=28904522

638. Meinero P, Mori L, Gasloli G. Endoscopic pilonidal sinus treatment (E.P.Si.T.). Tech Coloproctol [Internet]. 2014;18(4):389–92. Available from: http://ovidsp.ovid.com/ovidweb.cgi?T=JS&PAGE=reference&D=med11&NEWS=N&AN=23681300

639. Mendes CRS, Ferreira LS de M, Salim L. BRAZILIAN AND ARGENTINEAN MULTICENTRIC STUDY IN THE SURGICAL MINIMALLY INVASIVE TREATMENT OF PILONIDAL CYST. Arq Bras Cir Dig [Internet]. 2019;32(3):e1447. Available from: http://ovidsp.ovid.com/ovidweb.cgi?T=JS&PAGE=reference&D=med16&NEWS=N&AN=31644667

640. Meinero P, La Torre M, Lisi G, Stazi A, Carbone A, Regusci L, et al. Endoscopic pilonidal sinus treatment (EPSiT) in recurrent pilonidal disease: a prospective international multicenter study. Int J Colorectal Dis [Internet]. 2019;34(4):741–6. Available from: http://ovidsp.ovid.com/ovidweb.cgi?T=JS&PAGE=reference&D=med16&NEWS=N&AN=30719564

641. Pini Prato A, Mazzola C, Mattioli G, Escolino M, Esposito C, D’Alessio A, et al. Preliminary report on endoscopic pilonidal sinus treatment in children: results of a multicentric series. Pediatr Surg Int [Internet]. 2018;34(6):687–92. Available from: http://ovidsp.ovid.com/ovidweb.cgi?T=JS&PAGE=reference&D=med15&NEWS=N&AN=29675752

642. Jain Y, Javed MA, Singh S, Rout S, Joshi H, Rajaganeshan R. Endoscopic pilonidal abscess treatment: a novel approach for the treatment of pilonidal abscess. Ann R Coll Surg Engl [Internet]. 2017;99(2):134–6. Available from: http://ovidsp.ovid.com/ovidweb.cgi?T=JS&PAGE=reference&D=med14&NEWS=N&AN=27551895

643. Chia CLK, Tay VWY, Mantoo SK. Endoscopic pilonidal sinus treatment in the Asian population. Surg Laparosc Endosc Percutan Tech [Internet]. 2015;25(3):e95-7. Available from: http://ovidsp.ovid.com/ovidweb.cgi?T=JS&PAGE=reference&D=med12&NEWS=N&AN=26018049

644. Meinero P, Stazi A, Carbone A, Fasolini F, Regusci L, La Torre M. Endoscopic pilonidal sinus treatment: a prospective multicentre trial. Colorectal Dis [Internet]. 2016;18(5):O164-70. Available from: http://ovidsp.ovid.com/ovidweb.cgi?T=JS&PAGE=reference&D=med13&NEWS=N&AN=26946340

645. Korenkov M. Video-Assisted Subcutaneous Destruction of the Sinus Tract with Vessel-Loop Drainage as Minimally-Invasive Surgical Treatment for Pilonidal Sinus Disease. Surg Technol Int [Internet]. 2018;32:105–8. Available from: http://ovidsp.ovid.com/ovidweb.cgi?T=JS&PAGE=reference&D=med15&NEWS=N&AN=29529705

646. Esposito C, Gargiulo F, Izzo S, Cerulo M, Del Conte F, Severino G, et al. Pediatric Endoscopic Pilonidal Sinus Treatment: An Effective Procedure for Children with Recurrent Pilonidal Sinus Disease After Failed Open Surgery. J Laparoendosc Adv Surg Tech A [Internet]. 2019;29(7):981–6. Available from: http://ovidsp.ovid.com/ovidweb.cgi?T=JS&PAGE=reference&D=med16&NEWS=N&AN=30985239

647. Giarratano G, Mascagni P, Cianfarani A, Mosconi C, Saraceno F, Capuano I, et al. Su1787 - Muzi’s Tension Free Primary Closure Technique Versus the Endoscopic Pilonidal Sinus Treatment: A Retrospective Study. Gastroenterology [Internet]. 2018;154(6):S-1317. Available from: http://ovidsp.ovid.com/ovidweb.cgi?T=JS&PAGE=reference&D=emed19&NEWS=N&AN=2000864112

648. M. Z, D. K, D. K. Endoscopic pilonidal sinus treatment - EPSiT: Our initial experience. Color Dis [Internet]. 2018;20(Supplement 4):134. Available from: http://ovidsp.ovid.com/ovidweb.cgi?T=JS&PAGE=reference&D=emed19&NEWS=N&AN=624186248

649. C. E, M. E, S. I, F. T, M. C, G. S, et al. Pediatric endoscopic pilonidal sinus treatment (pepsit), a revolutionary technique to adopt in children with pilonidal sinus fistulas: A comparative study with classic open repair. J Laparoendosc Adv Surg Tech [Internet]. 2018;28(7):A28--A29. Available from: http://ovidsp.ovid.com/ovidweb.cgi?T=JS&PAGE=reference&D=emed19&NEWS=N&AN=623960592

650. Eastment J, Slater K. Outcomes of minimally invasive endoscopic pilonidal sinus surgery. Asian J Endosc Surg [Internet]. 2020 Jul 15;13(3):324–8. Available from: http://ovidsp.ovid.com/ovidweb.cgi?T=JS&PAGE=reference&D=emexb&NEWS=N&AN=629078994

651. G. G, S. F, V. T, L. M, S. B, F. F, et al. Endoscopic pilonidal sinus treatment (EPSiT): A multicentre study. Tech Coloproctol [Internet]. 2020;24(4):343. Available from: http://ovidsp.ovid.com/ovidweb.cgi?T=JS&PAGE=reference&D=emexb&NEWS=N&AN=632060580

652. G. G, E. T, E. G. Results of a prospective study for treatment of pilonidal sinus disease with a new video-assisted minimally invasive technique. Color Dis [Internet]. 2017;19(Supplement 2):129. Available from: http://ovidsp.ovid.com/ovidweb.cgi?T=JS&PAGE=reference&D=emed18&NEWS=N&AN=618607624

653. Manigrasso M, Velotti N, Sosa Fernandez LM, Vertaldi S, Maione F, Gennarelli N, et al. Endoscopic Approach to Recurrent Pilonidal Sinus: A Retrospective Analysis. J Laparoendosc Adv Surg Tech [Internet]. 2020 Jul 14;lap.2020.0252. Available from: http://ovidsp.ovid.com/ovidweb.cgi?T=JS&PAGE=reference&D=emexb&NEWS=N&AN=632385469

654. N. F. The minimally invasive treatment of Pilonidal disease with E.P.SI.T.: A retrospective analysis of long-term results in a single center. Tech Coloproctol [Internet]. 2020;24(4):382. Available from: http://ovidsp.ovid.com/ovidweb.cgi?T=JS&PAGE=reference&D=emexb&NEWS=N&AN=632060585

655. U.U. G, H. C, I.E. G, A. E. Crystalized phenol yields lower recurrence rates when used after endoscopic pilonidal sinus treatment. Color Dis [Internet]. 2015;17(SUPPL. 2):93. Available from: http://ovidsp.ovid.com/ovidweb.cgi?T=JS&PAGE=reference&D=emed16&NEWS=N&AN=72057753

656. Esposito C, Turrà F, Cerulo M, Del Conte F, Esposito G, Prato AP, et al. Technical standardization of MIS management of children with pilonidal sinus disease using pediatric endoscopic pilonidal sinus treatment (PEPSiT) and laser epilation. J Pediatr Surg [Internet]. 2020 Apr;55(4):761–6. Available from: http://www.elsevier.com/inca/publications/store/6/2/3/1/4/8/index.htt

657. L. M, P. M, A. S. EPSiT: Endoscopic pilonidal sinus treatment. Color Dis [Internet]. 2013;15(SUPPL. 3):10. Available from: http://ovidsp.ovid.com/ovidweb.cgi?T=JS&PAGE=reference&D=emed14&NEWS=N&AN=71211645

658. Fadgyas B, Garai GI, Ringwald Z. Paediatric endoscopic pilonidal sinus treatment: it is time to forget the open procedure? J Pediatr Endosc Surg [Internet]. 2020 Aug 1; Available from: https://link.springer.com/journal/42804

659. M.A. J, Y. J, S. S, S. R, H. J. Lunchtime Posters. Color Dis [Internet]. 2016 Sep;18(Supplement 1):27–43. Available from: http://ovidsp.ovid.com/ovidweb.cgi?T=JS&PAGE=reference&D=emed17&NEWS=N&AN=615291343

660. G. G, E. T, G. M, L. F, M. S, P. S. Endoscopic Pilonidal Sinus Treatment (E.P.Si.T): A new minimally invasive technique for the treatment of sacrococcygeal pilonidal sinus. Color Dis [Internet]. 2015;17(SUPPL. 2):11–2. Available from: http://ovidsp.ovid.com/ovidweb.cgi?T=JS&PAGE=reference&D=emed16&NEWS=N&AN=72057293

661. Kunduz E, Idiz UO, Aysan E, Guzel M, Yapalak Y, Baskoy L. Videoendoscopic pilonidal sinus surgery: early results with a new technique. Eur Surg [Internet]. 2017 Dec 3;49(6):275–8. Available from: http://www.springerlink.com/content/1682-8631

662. Meinero P, Mori L. Endoscopic pilonidal sinus treatment (E.P.Si.T): a new mininvasive procedure. J Am Coll Surg [Internet]. 2014 Oct;219(4):e75. Available from: http://ovidsp.ovid.com/ovidweb.cgi?T=JS&PAGE=reference&D=emed15&NEWS=N&AN=71676307

663. G. H, S.-L. O, H.-L. S, K.-Y. T. Comparison of endoscopic pilonidal sinus treatment versus pilonidal sinus flap surgery: A single institution experience within an Asian population. Color Dis [Internet]. 2018;20(Supplement 4):133. Available from: http://ovidsp.ovid.com/ovidweb.cgi?T=JS&PAGE=reference&D=emed19&NEWS=N&AN=624186154

664. G. G, M. M, C. G, P. M, C. T. New video-assisted minimally invasive technique for the treatment of pilonidal sinus: A prospective study. Color Dis [Internet]. 2014;16(SUPPL. 3):11. Available from: http://ovidsp.ovid.com/ovidweb.cgi?T=JS&PAGE=reference&D=emed15&NEWS=N&AN=71654901

665. Angerer C, Königsrainer I. Endoscopic pilonidal sinus treatment (EPSiT). coloproctology [Internet]. 2020 Aug 15;42(4):345–52. Available from: http://www.springerlink.com/content/101784/

666. C. M, M. S, A. M. Endoscopic pilonidal sinus treatment, for chronic symptomatic sacrococcygeal pilonidal sinus disease: Is it the right option? Tech Coloproctol [Internet]. 2017;21(10):839–40. Available from: http://ovidsp.ovid.com/ovidweb.cgi?T=JS&PAGE=reference&D=emed18&NEWS=N&AN=619389700

667. Jain Y, Singh S, Scott M, Rajaganeshan R. Endoscopic pilonidal abscess treatment (EPAT): A novel approach to manage acute pilonidal abscess. Int J Surg [Internet]. 2016 Nov;36(Supplement 1):S65. Available from: http://ovidsp.ovid.com/ovidweb.cgi?T=JS&PAGE=reference&D=emed17&NEWS=N&AN=619296984

668. Esposito C, Mendoza-Sagaon M, Del Conte F, Cerulo M, Coppola V, Esposito G, et al. Pediatric Endoscopic Pilonidal Sinus Treatment (PEPSiT) in Children With Pilonidal Sinus Disease: Tips and Tricks and New Structurated Protocol. Front Pediatr [Internet]. 2020 Jun 24;8:345. Available from: https://www.frontiersin.org/journals/pediatrics

669. M.G. P, E. K, S. S, E. K, S. A, K. T. 27th International Congress of the European Association for Endoscopic Surgery (EAES) Sevilla, Spain, 12–15 June 2019. Surg Endosc [Internet]. 2019 Oct 13;33(S2):485–781. Available from: http://ovidsp.ovid.com/ovidweb.cgi?T=JS&PAGE=reference&D=emexb&NEWS=N&AN=632125925

670. C. D, H.A. M, S. V, M. I, M. V, S. P. Video-assisted ablation of pilonidal sinus: New standard of care. Surg Endosc Other Interv Tech [Internet]. 2017;31(2 Supplement 1):S112. Available from: http://ovidsp.ovid.com/ovidweb.cgi?T=JS&PAGE=reference&D=emed18&NEWS=N&AN=617038854

671. Milone M, Bianco P, Musella M, Milone F. A technical modification of video-assisted ablation for recurrent pilonidal sinus. Colorectal Dis [Internet]. 2014;16(11):O404-6. Available from: http://ovidsp.ovid.com/ovidweb.cgi?T=JS&PAGE=reference&D=med11&NEWS=N&AN=25212710

672. Milone M, Musella M, Di Spiezio Sardo A, Bifulco G, Salvatore G, Sosa Fernandez LM, et al. Video-assisted ablation of pilonidal sinus: a new minimally invasive treatment--a pilot study. Surgery [Internet]. 2014;155(3):562–6. Available from: http://ovidsp.ovid.com/ovidweb.cgi?T=JS&PAGE=reference&D=med11&NEWS=N&AN=24300343

673. Gecim IE, Goktug UU, Celasin H. Endoscopic Pilonidal Sinus Treatment Combined With Crystalized Phenol Application May Prevent Recurrence. Dis Colon Rectum [Internet]. 2017;60(4):405–7. Available from: http://ovidsp.ovid.com/ovidweb.cgi?T=JS&PAGE=reference&D=med14&NEWS=N&AN=28267008

674. Esposito C, Izzo S, Turra F, Cerulo M, Severino G, Settimi A, et al. Pediatric Endoscopic Pilonidal Sinus Treatment, a Revolutionary Technique to Adopt in Children with Pilonidal Sinus Fistulas: Our Preliminary Experience. J Laparoendosc Adv Surg Tech A [Internet]. 2018;28(3):359–63. Available from: http://ovidsp.ovid.com/ovidweb.cgi?T=JS&PAGE=reference&D=med15&NEWS=N&AN=29232530

675. Suarez Valladares MJ, Rodriguez Prieto MA. Neodymium-doped yttrium aluminium garnet laser to treat primary pilonidal cysts: an alternative treatment. Br J Dermatol [Internet]. 2018;178(2):e127–8. Available from: http://ovidsp.ovid.com/ovidweb.cgi?T=JS&PAGE=reference&D=med15&NEWS=N&AN=28865111

676. Dragoni F, Moretti S, Cannarozzo G, Campolmi P. Treatment of recurrent pilonidal cysts with nd-YAG laser: report of our experience. J Dermatolog Treat [Internet]. 2018;29(1):65–7. Available from: http://ovidsp.ovid.com/ovidweb.cgi?T=JS&PAGE=reference&D=med15&NEWS=N&AN=28521574

677. Chupryna V V, Gatsenko AI. [The radical treatment of a suppurating epithelial coccygeal cyst and of a cyst of the sacrococcygeal area]. Radikal’noe lechenie nagnoivshegosia Ep kopchikovogo khoda i kisty kresttsovo-kopchikovoi Obl [Internet]. 1993;(1):25–6. Available from: http://ovidsp.ovid.com/ovidweb.cgi?T=JS&PAGE=reference&D=med3&NEWS=N&AN=10912023

678. Grubnik V V, Bakhar GA. [The use of a laser in the surgical treatment of an epithelial coccygeal cyst]. Primen lazera pri khirurgicheskom lechenii Ep kopchikovogo khoda [Internet]. 1993;(1):23–5. Available from: http://ovidsp.ovid.com/ovidweb.cgi?T=JS&PAGE=reference&D=med3&NEWS=N&AN=10912022

679. Klin B, Heller ON, Kaplan I. The use of the CO2 laser in pilonidal sinus disease: preliminary results of an ambulatory prospective study. J Clin Laser Med Surg [Internet]. 1990;8(1):31–7. Available from: http://ovidsp.ovid.com/ovidweb.cgi?T=JS&PAGE=reference&D=med3&NEWS=N&AN=10160877

680. Skobelkin OK, Tolstykh PI, Derbenev VA, Ste’nko VG, Kochurkov N V. [Carbon dioxide laser in the surgical treatment of proctologic diseases]. Uglekislotnyi lazer v khirurgicheskom lechenii proktologicheskikh Zabol [Internet]. 1989;143(9):3–5. Available from: http://ovidsp.ovid.com/ovidweb.cgi?T=JS&PAGE=reference&D=med3&NEWS=N&AN=2515648

681. Sadick NS, Yee-Levin J. Laser and light treatments for pilonidal cysts. Cutis [Internet]. 2006;78(2):125–8. Available from: http://ovidsp.ovid.com/ovidweb.cgi?T=JS&PAGE=reference&D=med6&NEWS=N&AN=16983902

682. Kurt F, Sözen S, Kanat BH, Kutluer N, Sakalli O, Gençtürk M, et al. Effect of platelet-rich plasma on healing in laser pilonidoplasty for pilonidal sinus disease. Lasers Med Sci [Internet]. 2020 Aug 29; Available from: http://link.springer.de/link/service/journals/10103/index.htm

683. Walfisch S, Mnitentag H, Baruchin AM, Sagi A. Nd:YAG and CO2 Lasers for the Treatment of Pilonidal Sinuses: Advantages over Traditional Techniques. Med Laser Appl [Internet]. 2004 Jan;19(3):155–9. Available from: http://ovidsp.ovid.com/ovidweb.cgi?T=JS&PAGE=reference&D=emed8&NEWS=N&AN=39665406

684. M. O-C, E. L, R. F. Pilonidal sinus laser ablation: Technique and experience in a single UK centre. Color Dis [Internet]. 2018;20(Supplement 7):57. Available from: http://ovidsp.ovid.com/ovidweb.cgi?T=JS&PAGE=reference&D=emed19&NEWS=N&AN=626683258

685. Dessily M, Dziubeck M, Chahidi E, Simonelli V. The SiLaC procedure for pilonidal sinus disease: long-term outcomes of a single institution prospective study. Tech Coloproctol [Internet]. 2019;23(12):1133–40. Available from: http://ovidsp.ovid.com/ovidweb.cgi?T=JS&PAGE=reference&D=medl&NEWS=N&AN=31773347

686. M. DA, A. M, V. A, P. G. Sinus laser ablation of pilonidal cysts (SiLac): Short-term clinical results of a new minimally invasive treatment of pylonidal cyst disease. Tech Coloproctol [Internet]. 2020;24(4):343. Available from: http://ovidsp.ovid.com/ovidweb.cgi?T=JS&PAGE=reference&D=emexb&NEWS=N&AN=632060596

687. R. G, E. L, M. C. Pilonidal sinus laser assisted closure (PiLAC)-A simple alternative to excision. Br J Surg [Internet]. 2019;106(Supplement 5):111. Available from: http://ovidsp.ovid.com/ovidweb.cgi?T=JS&PAGE=reference&D=emexb&NEWS=N&AN=631722138

688. Palesty JA, Zahir KS, Dudrick SJ, Ferri S, Tripodi G. Nd:YAG laser surgery for the excision of pilonidal cysts: a comparison with traditional techniques. Lasers Surg Med [Internet]. 2000;26(4):380–5. Available from: http://ovidsp.ovid.com/ovidweb.cgi?T=JS&PAGE=reference&D=med4&NEWS=N&AN=10805943

689. Dessily M, Charara F, Ralea S, Alle J-L. Pilonidal sinus destruction with a radial laser probe: technique and first Belgian experience. Acta Chir Belg [Internet]. 2017;117(3):164–8. Available from: http://ovidsp.ovid.com/ovidweb.cgi?T=JS&PAGE=reference&D=med14&NEWS=N&AN=28056720

690. Lindholt-Jensen CS, Lindholt JS, Beyer M, Lindholt JS. Nd-YAG laser treatment of primary and recurrent pilonidal sinus. Lasers Med Sci [Internet]. 2012;27(2):505–8. Available from: http://ovidsp.ovid.com/ovidweb.cgi?T=JS&PAGE=reference&D=med9&NEWS=N&AN=21927795

691. Georgiou GK. Outpatient laser treatment of primary pilonidal disease : the PiLaT technique. Tech Coloproctol [Internet]. 2018;22(10):773–8. Available from: http://ovidsp.ovid.com/ovidweb.cgi?T=JS&PAGE=reference&D=med15&NEWS=N&AN=30306277

692. Pappas AF, Christodoulou DK. A new minimally invasive treatment of pilonidal sinus disease with the use of a diode laser: a prospective large series of patients. Colorectal Dis [Internet]. 2018;20(8):O207–14. Available from: http://ovidsp.ovid.com/ovidweb.cgi?T=JS&PAGE=reference&D=med15&NEWS=N&AN=29878584

693. Gupta PJ. Radiofrequency incision and lay open technique of pilonidal sinus (clinical practice paper on modified technique). Kobe J Med Sci [Internet]. 2003;49(3–4):75–82. Available from: http://ovidsp.ovid.com/ovidweb.cgi?T=JS&PAGE=reference&D=med5&NEWS=N&AN=14970750

694. Gupta PJ. Radio surgery in pilonidal sinus: a new approach for the old problem. Acta Chir Belg [Internet]. 2005;105(2):183–6. Available from: http://ovidsp.ovid.com/ovidweb.cgi?T=JS&PAGE=reference&D=med6&NEWS=N&AN=15906911

695. Guyuron B, Dinner MI, Dowden R V. Excision and grafting in treatment of recurrent pilonidal sinus disease. Surg Gynecol Obstet [Internet]. 1983;156(2):201–4. Available from: http://ovidsp.ovid.com/ovidweb.cgi?T=JS&PAGE=reference&D=med2&NEWS=N&AN=6337419

696. Matino JJ, Banerjee S, Brown SH, Waldron JE. Human dermal tissue allograft use in treating chronic pilonidal sinus. Conn Med [Internet]. 2010;74(7):393–8. Available from: http://ovidsp.ovid.com/ovidweb.cgi?T=JS&PAGE=reference&D=med8&NEWS=N&AN=20806617

697. WENGER DS. Pilonidal cysts; their origin and treatment. Am J Surg [Internet]. 1950;80(2):242–3. Available from: http://ovidsp.ovid.com/ovidweb.cgi?T=JS&PAGE=reference&D=med1&NEWS=N&AN=15425719

698. Mosquera DA, Quayle JB. Bascom’s operation for pilonidal sinus. J R Soc Med [Internet]. 1995;88(1):45P-46P. Available from: http://ovidsp.ovid.com/ovidweb.cgi?T=JS&PAGE=reference&D=med3&NEWS=N&AN=7884771

699. Colov EP, Bertelsen CA. Short convalescence and minimal pain after out-patient Bascom’s pit-pick operation. Dan Med Bull [Internet]. 2011;58(12):A4348. Available from: http://ovidsp.ovid.com/ovidweb.cgi?T=JS&PAGE=reference&D=med8&NEWS=N&AN=22142576

700. Zorcolo L, Capra F, Scintu F, Casula G. [Surgical treatment of pilonidal disease. Results with the Bascom’s technique]. Tratt del sinus pilonidalis Esper con la Tec di Bascom [Internet]. 2004;59(4):387–95. Available from: http://ovidsp.ovid.com/ovidweb.cgi?T=JS&PAGE=reference&D=med5&NEWS=N&AN=15278034

701. C. B, N.F.S. W, S.A. L, J.N. L. A randomised trial of fibrin glue versus surgery for pilonidal disease: Results and long term follow up. Color Dis [Internet]. 2010;12(SUPPL. 3):20. Available from: http://ovidsp.ovid.com/ovidweb.cgi?T=JS&PAGE=reference&D=emed11&NEWS=N&AN=70325098

702. Bascom J. Pilonidal disease: long-term results of follicle removal. Dis Colon Rectum [Internet]. 1983;26(12):800–7. Available from: http://ovidsp.ovid.com/ovidweb.cgi?T=JS&PAGE=reference&D=med2&NEWS=N&AN=6641463

703. Senapati A, Cripps NP, Thompson MR. Bascom’s operation in the day-surgical management of symptomatic pilonidal sinus. Br J Surg [Internet]. 2000;87(8):1067–70. Available from: http://ovidsp.ovid.com/ovidweb.cgi?T=JS&PAGE=reference&D=med4&NEWS=N&AN=10931052

704. Doll D, Matevossian E, Hoenemann C, Hoffmann S. Incision and drainage preceding definite surgery achieves lower 20-year long-term recurrence rate in 583 primary pilonidal sinus surgery patients. J Dtsch Dermatol Ges [Internet]. 2013;11(1):60–4. Available from: http://ovidsp.ovid.com/ovidweb.cgi?T=JS&PAGE=reference&D=med10&NEWS=N&AN=23078365

705. Doll D, Evers T, Krapohl B, Matevossian E. Is there a difference in outcome (long-term recurrence rate) between emergency and elective pilonidal sinus surgery?. Minerva Chir [Internet]. 2013;68(2):199–205. Available from: http://ovidsp.ovid.com/ovidweb.cgi?T=JS&PAGE=reference&D=med10&NEWS=N&AN=23612234

706. Riedler L, Weimann S, Steiner E. [Operative therapy of the pilonidal sinus; 115 controlled patients (author’s transl)]. Beitrag zur Oper Ther des Sakraldermoids Bericht uber 115 kontrollierte Patienten [Internet]. 1978;103(21):1410–5. Available from: http://ovidsp.ovid.com/ovidweb.cgi?T=JS&PAGE=reference&D=med1&NEWS=N&AN=735518

707. O’Connor JJ. Surgery plus freezing as a technique for treating pilonidal disease. Dis Colon Rectum [Internet]. 1979;22(5):306–7. Available from: http://ovidsp.ovid.com/ovidweb.cgi?T=JS&PAGE=reference&D=med1&NEWS=N&AN=467193

708. Gage AA, Dutta P. Cryosurgery for pilonidal disease. Am J Surg [Internet]. 1977;133(2):249–54. Available from: http://ovidsp.ovid.com/ovidweb.cgi?T=JS&PAGE=reference&D=med1&NEWS=N&AN=835803

709. Rao AC. Cutting seton for pilonidal disease: a new approach. Tech Coloproctol [Internet]. 2006;10(3):242–4. Available from: http://ovidsp.ovid.com/ovidweb.cgi?T=JS&PAGE=reference&D=med6&NEWS=N&AN=16969609

710. Al-Mulhim AS, Sultan MA, Ahmed HH. Pilonidal sinus in males: to preserve or to obliterate the natal cleft. Saudi Med J [Internet]. 2002;23(7):875–6. Available from: http://ovidsp.ovid.com/ovidweb.cgi?T=JS&PAGE=reference&D=med4&NEWS=N&AN=12174247

711. Lynch JB, Laing AJ, Regan PJ. Vacuum-assisted closure therapy: a new treatment option for recurrent pilonidal sinus disease. Report of three cases. Dis Colon Rectum [Internet]. 2004;47(6):929–32. Available from: http://ovidsp.ovid.com/ovidweb.cgi?T=JS&PAGE=reference&D=med5&NEWS=N&AN=15129309

712. Duxbury MS, Blake SM, Dashfield A, Lambert AW. A randomised trial of knife versus diathermy in pilonidal disease. Ann R Coll Surg Engl [Internet]. 2003;85(6):405–7. Available from: http://ovidsp.ovid.com/ovidweb.cgi?T=JS&PAGE=reference&D=med5&NEWS=N&AN=14629883

713. Smid D, Novak P, Liska V, Treska V. [Pilonidal sinus--surgical management at our surgical clinic]. Pilonidalni sinus--chirurgicka lecba na nasem Pr [Internet]. 2011;90(5):301–5. Available from: http://ovidsp.ovid.com/ovidweb.cgi?T=JS&PAGE=reference&D=med8&NEWS=N&AN=21838135

714. Arslan NC, Atasoy G, Altintas T, Terzi C. Effect of triclosan-coated sutures on surgical site infections in pilonidal disease: prospective randomized study. Int J Colorectal Dis [Internet]. 2018 Oct 30;33(10):1445–52. Available from: http://link.springer.de/link/service/journals/00384/index.htm

715. K. Z, S. Z, S. B, S. S. Photodynamic therapy-a way to improve outcomes of pilonidal cyst and fistula surgery. Color Dis [Internet]. 2012;14(SUPPL. 2):67. Available from: http://ovidsp.ovid.com/ovidweb.cgi?T=JS&PAGE=reference&D=emed13&NEWS=N&AN=70926572

716. Zieger K. [Complications after surgery for pilonidal cyst. An introduction to a new debate on a “costly” disease]. Komplikationer efter Oper cystis pilonidalis Oplaeg til ny debat om en “kostbar” sygdom [Internet]. 1999;161(44):6056–8. Available from: http://ovidsp.ovid.com/ovidweb.cgi?T=JS&PAGE=reference&D=med4&NEWS=N&AN=10778342

717. Hagiga A, Aly M, Gultiaeva M, Murphy H. Using phenol for treating pilonidal sinus: a systematic review and meta-analysis. Eur J Plast Surg [Internet]. 2019 Jun 10;42(3):223–30. Available from: http://link.springer.de/link/service/journals/00238/index.htm

718. L. M, X. Q, B. M. Extensive surgery for pilonidal sinus with lumbar or perianal extension and associated sepsis. Color Dis [Internet]. 2009;11(SUPPL. 2):59. Available from: http://ovidsp.ovid.com/ovidweb.cgi?T=JS&PAGE=reference&D=emed11&NEWS=N&AN=70204781

719. L. P, G.M. L, A. J, C. D-V, D.M. B. Pilonidal sinus surgery in children: A novel approach. Color Dis [Internet]. 2019;21(Supplement 3):122. Available from: http://ovidsp.ovid.com/ovidweb.cgi?T=JS&PAGE=reference&D=emexb&NEWS=N&AN=631603901

720. Yuksel ME. Pilonidal sinus disease can be treated with crystallized phenol using a simple three-step technique. Acta dermatovenerologica Alpina, Pannonica, Adriat [Internet]. 2017;26(1):15–7. Available from: http://ovidsp.ovid.com/ovidweb.cgi?T=JS&PAGE=reference&D=med14&NEWS=N&AN=28352930

721. Girgin M, Kanat BH, Ayten R, Cetinkaya Z, Kanat Z, Bozdag A, et al. Minimally invasive treatment of pilonidal disease: crystallized phenol and laser depilation. Int Surg [Internet]. 2012;97(4):288–92. Available from: http://ovidsp.ovid.com/ovidweb.cgi?T=JS&PAGE=reference&D=med9&NEWS=N&AN=23294066

722. Kayaalp C, Aydin C. Review of phenol treatment in sacrococcygeal pilonidal disease. Tech Coloproctol [Internet]. 2009;13(3):189–93. Available from: http://ovidsp.ovid.com/ovidweb.cgi?T=JS&PAGE=reference&D=med7&NEWS=N&AN=19655223

723. Dogru O, Camci C, Aygen E, Girgin M, Topuz O. Pilonidal sinus treated with crystallized phenol: an eight-year experience. Dis Colon Rectum [Internet]. 2004;47(11):1934–8. Available from: http://ovidsp.ovid.com/ovidweb.cgi?T=JS&PAGE=reference&D=med5&NEWS=N&AN=15622588

724. Kelly SB, Graham WJ. Treatment of pilonidal sinus by phenol injection. Ulster Med J [Internet]. 1989;58(1):56–9. Available from: http://ovidsp.ovid.com/ovidweb.cgi?T=JS&PAGE=reference&D=med3&NEWS=N&AN=2773172

725. Olmez A, Kayaalp C, Aydin C. Treatment of pilonidal disease by combination of pit excision and phenol application. Tech Coloproctol [Internet]. 2013;17(2):201–6. Available from: http://ovidsp.ovid.com/ovidweb.cgi?T=JS&PAGE=reference&D=med10&NEWS=N&AN=23053444

726. Demirel AHPD, Polat M. Long-Term Outcomes of Crystallized Phenol Application after Punch Excision for the Treatment of Pilonidal Sinus Disease. Am Surg [Internet]. 2019;85(11):1219–23. Available from: http://ovidsp.ovid.com/ovidweb.cgi?T=JS&PAGE=reference&D=medl&NEWS=N&AN=31775962

727. Aygen E, Arslan K, Dogru O, Basbug M, Camci C. Crystallized phenol in nonoperative treatment of previously operated, recurrent pilonidal disease. Dis Colon Rectum [Internet]. 2010;53(6):932–5. Available from: http://ovidsp.ovid.com/ovidweb.cgi?T=JS&PAGE=reference&D=med8&NEWS=N&AN=20485008

728. Kayaalp C, Olmez A, Aydin C, Piskin T, Kahraman L. Investigation of a one-time phenol application for pilonidal disease. Med Princ Pract [Internet]. 2010;19(3):212–5. Available from: http://ovidsp.ovid.com/ovidweb.cgi?T=JS&PAGE=reference&D=med8&NEWS=N&AN=20357505

729. Kaymakcioglu N, Yagci G, Simsek A, Unlu A, Tekin OF, Cetiner S, et al. Treatment of pilonidal sinus by phenol application and factors affecting the recurrence. Tech Coloproctol [Internet]. 2005;9(1):21–4. Available from: http://ovidsp.ovid.com/ovidweb.cgi?T=JS&PAGE=reference&D=med6&NEWS=N&AN=15868494

730. Schneider IH, Thaler K, Kockerling F. Treatment of pilonidal sinuses by phenol injections. Int J Colorectal Dis [Internet]. 1994;9(4):200–2. Available from: http://ovidsp.ovid.com/ovidweb.cgi?T=JS&PAGE=reference&D=med3&NEWS=N&AN=7876724

731. Vara-Thorbeck R, Mekinassi K, Berchid S. Phenol treatment of pilonidal sinuses. Zentralbl Chir [Internet]. 1990;115(12):777–80. Available from: http://ovidsp.ovid.com/ovidweb.cgi?T=JS&PAGE=reference&D=med3&NEWS=N&AN=2385975

732. Hegge HG, Vos GA, Patka P, Hoitsma HF. Treatment of complicated or infected pilonidal sinus disease by local application of phenol. Surgery [Internet]. 1987;102(1):52–4. Available from: http://ovidsp.ovid.com/ovidweb.cgi?T=JS&PAGE=reference&D=med2&NEWS=N&AN=3589976

733. Shorey BA. Pilonidal sinus treated by phenol injection. Br J Surg [Internet]. 1975;62(5):407–8. Available from: http://ovidsp.ovid.com/ovidweb.cgi?T=JS&PAGE=reference&D=med1&NEWS=N&AN=1139139

734. MAURICE BA, GREENWOOD RK. A CONSERVATIVE TREATMENT OF PILONIDAL SINUS. Br J Surg [Internet]. 1964;51:510–2. Available from: http://ovidsp.ovid.com/ovidweb.cgi?T=JS&PAGE=reference&D=med1&NEWS=N&AN=14199061

735. M.K. A. Phenol application combined with minimally invasive surgery for pilonidal sinus treatment. Anatol J Clin Investig [Internet]. 2011;5(4):165–8. Available from: http://www.ajcionline.org/index.php/ajci/article/view/529/441

736. Sakçak İ, Avşar FM, Coşgun E. Comparison of the Application of Low Concentration and 80% Phenol Solution in Pilonidal Sinus Disease. JRSM Short Rep [Internet]. 2010 Jun 30;1(1):1–5. Available from: http://shr.sagepub.com/

737. Girgin M, Kanat BH. The Results of a One-Time Crystallized Phenol Application for Pilonidal Sinus Disease. Indian J Surg [Internet]. 2014 Feb 16;76(1):17–20. Available from: http://search.ebscohost.com/login.aspx?direct=true&db=cin20&AN=103936244&site=ehost-live

738. Owen-Smith MS. Phenol Irrigation for Pilonidal Sinus. J R Army Med Corps [Internet]. 1975 Apr 1;121(2):79–86. Available from: http://ovidsp.ovid.com/ovidweb.cgi?T=JS&PAGE=reference&D=emed2&NEWS=N&AN=6091453

739. Stray O. [Pilonidal sinus. Treatment by phenol injection]. Tidsskr Nor Laegeforen [Internet]. 1976 Jan 30;96(3):151–3. Available from: http://ovidsp.ovid.com/ovidweb.cgi?T=JS&PAGE=reference&D=emed2&NEWS=N&AN=7003850

740. Dag A, Colak T, Turkmenoglu O, Sozutek A, Gundogdu R. Phenol procedure for pilonidal sinus disease and risk factors for treatment failure. Surgery [Internet]. 2012;151(1):113–7. Available from: http://ovidsp.ovid.com/ovidweb.cgi?T=JS&PAGE=reference&D=med9&NEWS=N&AN=21982072

741. Ates U, Ergun E, Gollu G, Sozduyar S, Kologlu M, Cakmak M, et al. Pilonidal sinus disease surgery in children: the first study to compare crystallized phenol application to primary excision and closure. J Pediatr Surg [Internet]. 2018;53(3):452–5. Available from: http://ovidsp.ovid.com/ovidweb.cgi?T=JS&PAGE=reference&D=med15&NEWS=N&AN=28549686

742. Handmer M. Sticking to the facts: a systematic review of fibrin glue for pilonidal disease. ANZ J Surg [Internet]. 2012;82(4):221–4. Available from: http://ovidsp.ovid.com/ovidweb.cgi?T=JS&PAGE=reference&D=med9&NEWS=N&AN=22510177

743. Lund J, Tou S, Doleman B, Williams JP. Fibrin glue for pilonidal sinus disease. Cochrane database Syst Rev [Internet]. 2017;1:CD011923. Available from: http://ovidsp.ovid.com/ovidweb.cgi?T=JS&PAGE=reference&D=med14&NEWS=N&AN=28085995

744. Alamdari DH, Motie MR, Kamalahmadi N, Aliakbarian M. Autologous Platelet-Rich Plasma and Fibrin Glue Decrease Pain Following Excision and Primary Closure of Pilonidal Sinus. Adv Skin Wound Care [Internet]. 2019;32(5):234–7. Available from: http://ovidsp.ovid.com/ovidweb.cgi?T=JS&PAGE=reference&D=med16&NEWS=N&AN=31008759

745. T. S. Fibrin glue is a simple, quick and effective treatment for pilonidal sinus disease. Color Dis [Internet]. 2017;19(Supplement 2):133. Available from: http://ovidsp.ovid.com/ovidweb.cgi?T=JS&PAGE=reference&D=emed18&NEWS=N&AN=618608169

746. Hardy E, Herrod P, Sian T, Boyd-Carson H, Blackwell J, Lund JN, et al. Fibrin glue obliteration is safe, effective and minimally invasive as first line treatment for pilonidal sinus disease in children. J Pediatr Surg [Internet]. 2019;54(8):1668–70. Available from: http://ovidsp.ovid.com/ovidweb.cgi?T=JS&PAGE=reference&D=med16&NEWS=N&AN=30268489

747. Sian TS, Herrod PJJ, Blackwell JEM, Hardy EJO, Lund JN. Fibrin glue is a quick and effective treatment for primary and recurrent pilonidal sinus disease. Tech Coloproctol [Internet]. 2018;22(10):779–84. Available from: http://ovidsp.ovid.com/ovidweb.cgi?T=JS&PAGE=reference&D=med15&NEWS=N&AN=30413996

748. Elsey E, Lund JN. Fibrin glue in the treatment for pilonidal sinus: high patient satisfaction and rapid return to normal activities. Tech Coloproctol [Internet]. 2013;17(1):101–4. Available from: http://ovidsp.ovid.com/ovidweb.cgi?T=JS&PAGE=reference&D=med10&NEWS=N&AN=23224857

749. Seleem MI, Al-Hashemy AM. Management of pilonidal sinus using fibrin glue: a new concept and preliminary experience. Colorectal Dis [Internet]. 2005;7(4):319–22. Available from: http://ovidsp.ovid.com/ovidweb.cgi?T=JS&PAGE=reference&D=med6&NEWS=N&AN=15932551

750. Saedon M, Chin A, Alfa-Wali M, Khoo CK, Varma A. Minimally Invasive Fibrin Sealant Application in Pilonidal Sinus: A Comparative Study. Prague Med Rep [Internet]. 2018;119(2–3):107–12. Available from: http://ovidsp.ovid.com/ovidweb.cgi?T=JS&PAGE=reference&D=med15&NEWS=N&AN=30414361

751. Patti R, Angileri M, Migliore G, Sparancello M, Termine S, Crivello F, et al. Use of fibrin glue in the treatment of pilonidal sinus disease: a pilot study. G Chir [Internet]. 2006;27(8–9):331–4. Available from: http://ovidsp.ovid.com/ovidweb.cgi?T=JS&PAGE=reference&D=med6&NEWS=N&AN=17064495

752. Lund JN, Leveson SH. Fibrin glue in the treatment of pilonidal sinus: results of a pilot study. Dis Colon Rectum [Internet]. 2005;48(5):1094–6. Available from: http://ovidsp.ovid.com/ovidweb.cgi?T=JS&PAGE=reference&D=med6&NEWS=N&AN=15868239

753. Isik A, Eryılmaz R, Okan I, Dasiran F, Firat D, Idiz O, et al. The use of fibrin glue without surgery in the treatment of pilonidal sinus disease. Int J Clin Exp Med [Internet]. 2014;7(4):1047–51. Available from: http://www.ijcem.com/files/ijcem1402021.pdf

754. Nguyen AL, Pronk AA, Furnee EJB, Pronk A, Davids PHP, Smakman N. Local administration of gentamicin collagen sponge in surgical excision of sacrococcygeal pilonidal sinus disease: a systematic review and meta-analysis of the literature. Tech Coloproctol [Internet]. 2016;20(2):91–100. Available from: http://ovidsp.ovid.com/ovidweb.cgi?T=JS&PAGE=reference&D=med13&NEWS=N&AN=26546004

755. E. F, L. N, P. D, A. P. Local administration of gentamicin collagen sponge in surgical excision of sacrococcygeal pilonidal sinus disease: A systematic review and meta-analysis of the literature. Color Dis [Internet]. 2015;17(SUPPL. 2):93. Available from: http://ovidsp.ovid.com/ovidweb.cgi?T=JS&PAGE=reference&D=emed16&NEWS=N&AN=72057748

756. Brieler HS. [Infected pilonidal sinus]. Der infizierte Sinus pilonidalis [Internet]. 1997;114:497–500. Available from: http://ovidsp.ovid.com/ovidweb.cgi?T=JS&PAGE=reference&D=med4&NEWS=N&AN=9574192

757. Sondenaa K, Nesvik I, Gullaksen FP, Furnes A, Harbo SO, Weyessa S, et al. The role of cefoxitin prophylaxis in chronic pilonidal sinus treated with excision and primary suture. J Am Coll Surg [Internet]. 1995;180(2):157–60. Available from: http://ovidsp.ovid.com/ovidweb.cgi?T=JS&PAGE=reference&D=med3&NEWS=N&AN=7850048

758. Sondenaa K, Nesvik I, Andersen E, Natas O, Soreide JA. Bacteriology and complications of chronic pilonidal sinus treated with excision and primary suture. Int J Colorectal Dis [Internet]. 1995;10(3):161–6. Available from: http://ovidsp.ovid.com/ovidweb.cgi?T=JS&PAGE=reference&D=med3&NEWS=N&AN=7561435

759. Lundhus E, Gottrup F. Outcome at three to five years of primary closure of perianal and pilonidal abscess. A randomised, double-blind clinical trial with a complete three-year followup of one compared with four days’ treatment with ampicillin and metronidazole. Eur J Surg [Internet]. 1993;159(10):555–8. Available from: http://ovidsp.ovid.com/ovidweb.cgi?T=JS&PAGE=reference&D=med3&NEWS=N&AN=8286514

760. Vogel P, Lenz J. [Treatment of pilonidal sinus with excision and primary suture using a local, resorbable antibiotic carrier. Results of a prospective randomized study]. Die Behandlung des Sinus pilonidalis mittels Excision und Primarnaht unter Verwendung eines Lokal resorbierbaren Antibiot Ergebnisse einer prospektiven, randomisierten Untersuchung [Internet]. 1992;63(9):748–53. Available from: http://ovidsp.ovid.com/ovidweb.cgi?T=JS&PAGE=reference&D=med3&NEWS=N&AN=1395879

761. Lundhus E, Gjode P, Gottrup F, Holm CN, Terpling S. Bactericidal antimicrobial cover in primary suture of perianal or pilonidal abscess. A prospective, randomized, double-blind clinical trial. Acta Chir Scand [Internet]. 1989;155(6–7):351–4. Available from: http://ovidsp.ovid.com/ovidweb.cgi?T=JS&PAGE=reference&D=med3&NEWS=N&AN=2683535

762. Karip AB, Celik K, Aydin T, Yazicilar H, Iscan Y, Agalar C, et al. Effect of Triclosan-Coated Suture and Antibiotic Prophylaxis on Infection and Recurrence after Karydakis Flap Repair for Pilonidal Disease: A Randomized Parallel-Arm Double-Blinded Clinical Trial. Surg Infect (Larchmt) [Internet]. 2016;17(5):583–8. Available from: http://ovidsp.ovid.com/ovidweb.cgi?T=JS&PAGE=reference&D=med13&NEWS=N&AN=27383814

763. Kundes MF, Cetin K, Kement M, Kaptanoglu L, Civil O, Haksal M, et al. Does prophylactic antibiotic reduce surgical site infections after rhomboid excision and Limberg flap for pilonidal disease: a prospective randomized double blind study. Int J Colorectal Dis [Internet]. 2016;31(5):1089–91. Available from: http://ovidsp.ovid.com/ovidweb.cgi?T=JS&PAGE=reference&D=med13&NEWS=N&AN=26525054

764. Ozbalci GS, Tuncal S, Bayraktar K, Tasova V, Ali Akkus M. Is gentamicin-impregnated collagen sponge to be recommended in pilonidal sinus patient treated with marsupialization? A prospective randomized study. Ann Ital Chir [Internet]. 2014;85(6):576–82. Available from: http://ovidsp.ovid.com/ovidweb.cgi?T=JS&PAGE=reference&D=med11&NEWS=N&AN=25711716

765. Yetim I, Ozkan O V, Dervisoglu A, Erzurumlu K, Canbolant E. Effect of gentamicin-absorbed collagen in wound healing in pilonidal sinus surgery: a prospective randomized study. J Int Med Res [Internet]. 2010;38(3):1029–33. Available from: http://ovidsp.ovid.com/ovidweb.cgi?T=JS&PAGE=reference&D=med8&NEWS=N&AN=20819439

766. Andersson RE, Lukas G, Skullman S, Hugander A. Local administration of antibiotics by gentamicin-collagen sponge does not improve wound healing or reduce recurrence rate after pilonidal excision with primary suture: a prospective randomized controlled trial. World J Surg [Internet]. 2010;34(12):3042–8. Available from: http://ovidsp.ovid.com/ovidweb.cgi?T=JS&PAGE=reference&D=med8&NEWS=N&AN=20734046

767. Chaudhuri A, Bekdash BA, Taylor AL. Single-dose metronidazole vs 5-day multi-drug antibiotic regimen in excision of pilonidal sinuses with primary closure: a prospective, randomized, double-blinded pilot study. Int J Colorectal Dis [Internet]. 2006;21(7):688–92. Available from: http://ovidsp.ovid.com/ovidweb.cgi?T=JS&PAGE=reference&D=med6&NEWS=N&AN=16362397

768. Sondenaa K, Diab R, Nesvik I, Gullaksen FP, Kristiansen RM, Saebo A, et al. Influence of failure of primary wound healing on subsequent recurrence of pilonidal sinus. combined prospective study and randomised controlled trial. Eur J Surg [Internet]. 2002;168(11):614–8. Available from: http://ovidsp.ovid.com/ovidweb.cgi?T=JS&PAGE=reference&D=med4&NEWS=N&AN=12699097

769. Chaudhuri A, Bekdash BA. Single-dose metronidazole versus 5-day multi-drug antibiotic regimen in excision of pilonidal sinuses with primary closure: a prospective randomised controlled double-blinded study. Int J Colorectal Dis [Internet]. 2002;17(5):355–8. Available from: http://ovidsp.ovid.com/ovidweb.cgi?T=JS&PAGE=reference&D=med4&NEWS=N&AN=12420730

770. Calis H, Guler Y, Sengul S, Karabulut Z. The effects of perioperative antibiotherapy on surgical site infections in sacrococcygeal pilonidal sinus treated with rhomboid excision and Limberg transposition procedure. Int Wound J [Internet]. 2019;16(4):974–8. Available from: http://ovidsp.ovid.com/ovidweb.cgi?T=JS&PAGE=reference&D=medl&NEWS=N&AN=30938077

771. Bunke HJ, Schultheis A, Meyer G, Dusel W. [Surgical revision of the pilonidal sinus with single shot antibiosis]. Die Oper Sanierung des Sinus pilonidalis unter Single-shot-Antibiose [Internet]. 1995;66(3):220–3. Available from: http://ovidsp.ovid.com/ovidweb.cgi?T=JS&PAGE=reference&D=med3&NEWS=N&AN=7750394

772. HAMILTON JE, CATTANACH LM. Comparative study of chemotherapies in the surgery of pilonidal sinus. Am J Surg [Internet]. 1947;74(4):449–54. Available from: http://ovidsp.ovid.com/ovidweb.cgi?T=JS&PAGE=reference&D=med1&NEWS=N&AN=20266025

773. Doll D, Evers T, Matevossian E, Hoffmann S, Krapohl B, Bartsch D. Does gentamycin affect long term recurrence rate in pilonidal sinus surgery? Eur Surg [Internet]. 2011 Aug 3;43(4):236–43. Available from: http://ovidsp.ovid.com/ovidweb.cgi?T=JS&PAGE=reference&D=emed12&NEWS=N&AN=51547569

774. Doll D, Novotny A, Rothe R, Kristiansen JE, Wietelmann K, Boulesteix A-L, et al. Methylene Blue halves the long-term recurrence rate in acute pilonidal sinus disease. Int J Colorectal Dis [Internet]. 2008;23(2):181–7. Available from: http://ovidsp.ovid.com/ovidweb.cgi?T=JS&PAGE=reference&D=med7&NEWS=N&AN=17960395

775. Ardelt M, Kocijan R, Dittmar Y, Fahrner R, Rauchfuss F, Scheuerlein H, et al. Effects of methylene-blue staining on the extent of pilonidal sinus excision. J Wound Care [Internet]. 2016;25(6):342–7. Available from: http://ovidsp.ovid.com/ovidweb.cgi?T=JS&PAGE=reference&D=med13&NEWS=N&AN=27286667

776. Kuvvetli A, Çetinkunar S, Parlakgümüş A. Utility of Methylene Blue Guided Limberg Flap on Longterm Recurrence in Adult Chronic Pilonidal Disease. Turkish J Color Dis [Internet]. 2019 Sep 1;29(3):118–20. Available from: http://search.ebscohost.com/login.aspx?direct=true&db=cin20&AN=138972914&site=ehost-live

777. Sahin A, Olcucuoglu E, Seker D, Kulacoglu H. The effect of using methylene blue in surgical treatments of pilonidal disease: a prospective randomized study. Eur Surg [Internet]. 2014 Aug 19;46(4):148–54. Available from: http://ovidsp.ovid.com/ovidweb.cgi?T=JS&PAGE=reference&D=emexb&NEWS=N&AN=53249293

778. Idiz UO, Aysan E, Firat D, Bozkurt S, Buyukpinarbasili N, Muslumanoglu M. Safety and/or effectiveness of methylene blue-guided pilonidal sinus surgery. Int J Clin Exp Med [Internet]. 2014;7(4):927–31. Available from: http://www.ijcem.com/files/ijcem0000214.pdf

779. Gipponi M, Reboa G, Testa T, Giannini G, Strada P. Tension-free primary closure with autologous platelet gel versus Vivostat- for the definitive treatment of chronic sacrococcygeal pilonidal disease. In Vivo [Internet]. 2010;24(4):583–9. Available from: http://ovidsp.ovid.com/ovidweb.cgi?T=JS&PAGE=reference&D=med8&NEWS=N&AN=20668329

780. Elbanna HG, Emile SH, Youssef M, Thabet W, El-Hamed TMA, Ghnnam WM. Novel Approach of Treatment of Pilonidal Sinus Disease With Thrombin Gelatin Matrix as a Sealant. Dis Colon Rectum [Internet]. 2016;59(8):775–80. Available from: http://ovidsp.ovid.com/ovidweb.cgi?T=JS&PAGE=reference&D=med13&NEWS=N&AN=27384096

781. Baldelli CMF, Ruella M, Scuderi S, Monni M, Passera R, Omede P, et al. A short course of granulocyte-colony-stimulating factor to accelerate wound repair in patients undergoing surgery for sacrococcygeal pilonidal cyst: proof of concept. Cytotherapy [Internet]. 2012;14(9):1101–9. Available from: http://ovidsp.ovid.com/ovidweb.cgi?T=JS&PAGE=reference&D=med9&NEWS=N&AN=22783986

782. Arslan NC, Degirmenci AK, Ozdenkaya Y, Terzi C. Wound Irrigation with Chlorhexidine Gluconate Reduces Surgical Site Infection in Pilonidal Disease: Single-Blind Prospective Study. Surg Infect (Larchmt) [Internet]. 2020 Mar 1;21(2):143–9. Available from: http://www.liebertonline.com/sur

783. Halleran DR, Onwuka AJ, Lawrence AE, Fischer BC, Deans KJ, Minneci PC. Laser Hair Depilation in the Treatment of Pilonidal Disease: A Systematic Review. Surg Infect (Larchmt) [Internet]. 2018;19(6):566–72. Available from: http://ovidsp.ovid.com/ovidweb.cgi?T=JS&PAGE=reference&D=med15&NEWS=N&AN=30095368

784. Pronk AA, Eppink L, Smakman N, Furnee EJB. The effect of hair removal after surgery for sacrococcygeal pilonidal sinus disease: a systematic review of the literature. Tech Coloproctol [Internet]. 2018;22(1):7–14. Available from: http://ovidsp.ovid.com/ovidweb.cgi?T=JS&PAGE=reference&D=med15&NEWS=N&AN=29185064

785. A. P, L. E, N. S. The effect of hair removal after surgery for sacrococcygeal pilonidal sinus disease: A systematic review of the literature. Color Dis [Internet]. 2015;17(SUPPL. 2):92. Available from: http://ovidsp.ovid.com/ovidweb.cgi?T=JS&PAGE=reference&D=emed16&NEWS=N&AN=72057747

786. Minneci PC, Halleran DR, Lawrence AE, Fischer BA, Cooper JN, Deans KJ. Laser hair depilation for the prevention of disease recurrence in adolescents and young adults with pilonidal disease: study protocol for a randomized controlled trial. Trials [Internet]. 2018;19(1):599. Available from: http://ovidsp.ovid.com/ovidweb.cgi?T=JS&PAGE=reference&D=med15&NEWS=N&AN=30382903

787. Kelati A, Lagrange S, Le Duff F, Lacour J-P, Benasaid R, Breaud J, et al. Laser hair removal after surgery vs. surgery alone for the treatment of pilonidal cysts: a retrospective case-control study. J Eur Acad Dermatol Venereol [Internet]. 2018;32(11):2031–3. Available from: http://ovidsp.ovid.com/ovidweb.cgi?T=JS&PAGE=reference&D=med15&NEWS=N&AN=29633368

788. Abbas O, Sidani M, Rubeiz N, Ghosn S, Kibbi AG. Letter: 755-nm Alexandrite laser epilation as an adjuvant and primary treatment for pilonidal sinus disease. Dermatol Surg [Internet]. 2010;36(3):430–2. Available from: http://ovidsp.ovid.com/ovidweb.cgi?T=JS&PAGE=reference&D=med8&NEWS=N&AN=20402950

789. Conroy FJ, Kandamany N, Mahaffey PJ. Laser depilation and hygiene: preventing recurrent pilonidal sinus disease. J Plast Reconstr Aesthet Surg [Internet]. 2008;61(9):1069–72. Available from: http://ovidsp.ovid.com/ovidweb.cgi?T=JS&PAGE=reference&D=med7&NEWS=N&AN=17664085

790. Badawy EA, Kanawati MN. Effect of hair removal by Nd:YAG laser on the recurrence of pilonidal sinus. J Eur Acad Dermatol Venereol [Internet]. 2009;23(8):883–6. Available from: http://ovidsp.ovid.com/ovidweb.cgi?T=JS&PAGE=reference&D=med7&NEWS=N&AN=19586514

791. Odili J, Gault D. Laser depilation of the natal cleft--an aid to healing the pilonidal sinus. Ann R Coll Surg Engl [Internet]. 2002;84(1):29–32. Available from: http://ovidsp.ovid.com/ovidweb.cgi?T=JS&PAGE=reference&D=med4&NEWS=N&AN=11890622

792. Stirnemann H, Blasimann B. [Is preventive epilation following sacral dermoid operation useful or ineffective?]. Ist die Epilationsprophylaxe nach Sacraldermoidoperation Nutzl oder wirkungslos? [Internet]. 1983;54(8):548–9. Available from: http://ovidsp.ovid.com/ovidweb.cgi?T=JS&PAGE=reference&D=med2&NEWS=N&AN=6617342

793. Petersen S, Wietelmann K, Evers T, Huser N, Matevossian E, Doll D. Long-term effects of postoperative razor epilation in pilonidal sinus disease. Dis Colon Rectum [Internet]. 2009;52(1):131–4. Available from: http://ovidsp.ovid.com/ovidweb.cgi?T=JS&PAGE=reference&D=med7&NEWS=N&AN=19273968

794. Shafigh Y, Beheshti A, Charkhchian M, Rad FS. Successful treatment of pilonidal disease by intense pulsed light device. Adv Clin Exp Med [Internet]. 2014;23(2):277–82. Available from: http://ovidsp.ovid.com/ovidweb.cgi?T=JS&PAGE=reference&D=med11&NEWS=N&AN=24913119

795. Schulze SM, Patel N, Hertzog D, Fares LG 2nd. Treatment of pilonidal disease with laser epilation. Am Surg [Internet]. 2006;72(6):534–7. Available from: http://ovidsp.ovid.com/ovidweb.cgi?T=JS&PAGE=reference&D=med6&NEWS=N&AN=16808209

796. Landa N, Aller O, Landa-Gundin N, Torrontegui J, Azpiazu JL. Successful treatment of recurrent pilonidal sinus with laser epilation. Dermatol Surg [Internet]. 2005;31(6):726–8. Available from: http://ovidsp.ovid.com/ovidweb.cgi?T=JS&PAGE=reference&D=med6&NEWS=N&AN=15996432

797. Benedetto A V, Lewis AT. Pilonidal sinus disease treated by depilation using an 800 nm diode laser and review of the literature. Dermatol Surg [Internet]. 2005;31(5):587–91. Available from: http://ovidsp.ovid.com/ovidweb.cgi?T=JS&PAGE=reference&D=med6&NEWS=N&AN=15962749

798. G. F, K. K, H. M. Laser hair removal for recurrent pilonidal sinus disease. Does it really work? Br J Dermatol [Internet]. 2015;173(SUPPL. 1):103–4. Available from: http://ovidsp.ovid.com/ovidweb.cgi?T=JS&PAGE=reference&D=emed16&NEWS=N&AN=71969585

799. T. D, C. S. Intense pulsed light (IPL) hair removal to prevent pilonidal sinus disease (PND). J Am Acad Dermatol [Internet]. 2016;74(5 SUPPL. 1):AB289. Available from: http://ovidsp.ovid.com/ovidweb.cgi?T=JS&PAGE=reference&D=emed17&NEWS=N&AN=72276090

800. Alvear DT. Non-surgical management of pilonidal disease - is it feasible? J Am Coll Surg [Internet]. 2014 Oct;219(4):e129. Available from: http://ovidsp.ovid.com/ovidweb.cgi?T=JS&PAGE=reference&D=emed15&NEWS=N&AN=71676445

801. Kok K, Osmani OA, Odeke M, Eltigani EA. Recurrent pilonidal sinus disease: Do lasers have the answer? Med Laser Appl [Internet]. 2011 May;26(2):62–6. Available from: http://ovidsp.ovid.com/ovidweb.cgi?T=JS&PAGE=reference&D=emed12&NEWS=N&AN=51309165

802. S. O, A. A, M. T. Laser hair epilation: Is there a role in pilonidal sinus disease? Br J Dermatol [Internet]. 2018;179(Supplement 1):123. Available from: http://ovidsp.ovid.com/ovidweb.cgi?T=JS&PAGE=reference&D=emed19&NEWS=N&AN=623095977

803. I.I. B, M. N, R. A, K. H, M. S. Use of laser hair removal after pilonidal surgery. Med Forum Mon [Internet]. 2017;28(4):152–4. Available from: http://medforum.pk/images/pdf/2017/april2017.pdf

804. Lukish JR, Kindelan T, Marmon LM, Pennington M, Norwood C. Laser epilation is a safe and effective therapy for teenagers with pilonidal disease. J Pediatr Surg [Internet]. 2009;44(1):282–5. Available from: http://ovidsp.ovid.com/ovidweb.cgi?T=JS&PAGE=reference&D=med7&NEWS=N&AN=19159757

805. Lopez JJ, Cooper JN, Fischer BA, Gonzalez DO, Deans KJ, Minneci PC. Safety and Tolerability of Laser Hair Depilation in Pilonidal Disease: A Pilot Study. Surg Infect (Larchmt) [Internet]. 2017;18(8):890–3. Available from: http://ovidsp.ovid.com/ovidweb.cgi?T=JS&PAGE=reference&D=med14&NEWS=N&AN=29016243

806. Butter A, Hanson M, VanHouwelingen L, Merritt N, Seabrook J. Hair epilation versus surgical excision as primary management of pilonidal disease in the pediatric population. Can J Surg [Internet]. 2015;58(3):209–11. Available from: http://ovidsp.ovid.com/ovidweb.cgi?T=JS&PAGE=reference&D=med12&NEWS=N&AN=26011854

807. Khan MAA, Javed AA, Govindan KS, Rafiq S, Thomas K, Baker L, et al. Control of hair growth using long-pulsed alexandrite laser is an efficient and cost effective therapy for patients suffering from recurrent pilonidal disease. Lasers Med Sci [Internet]. 2016;31(5):857–62. Available from: http://ovidsp.ovid.com/ovidweb.cgi?T=JS&PAGE=reference&D=med13&NEWS=N&AN=27003897

808. Oram Y, Kahraman F, Karincaoglu Y, Koyuncu E. Evaluation of 60 patients with pilonidal sinus treated with laser epilation after surgery. Dermatol Surg [Internet]. 2010;36(1):88–91. Available from: http://ovidsp.ovid.com/ovidweb.cgi?T=JS&PAGE=reference&D=med8&NEWS=N&AN=20002644

809. Cevik M, Dorterler ME, Abbasoglu L. Is conservative treatment an effective option for pilonidal sinus disease in children?. Int Wound J [Internet]. 2018;15(5):840–4. Available from: http://ovidsp.ovid.com/ovidweb.cgi?T=JS&PAGE=reference&D=med15&NEWS=N&AN=29947121

810. Hurst DW. The evolution of management of pilonidal sinus disease. Can J Surg [Internet]. 1984;27(6):603–5. Available from: http://ovidsp.ovid.com/ovidweb.cgi?T=JS&PAGE=reference&D=med2&NEWS=N&AN=6388772

811. K. B, P.N. R, A. S, B.N. M. Comparative clinical study of jatyadi varti and aragwadadi varti in the management of nadi vrana (Pilonidal sinus). Int Res J Pharm [Internet]. 2012;3(3):135–8. Available from: http://www.irjponline.com/admin/php/uploads/918_pdf.pdf

812. Dumville JC, Owens GL, Crosbie EJ, Peinemann F, Liu Z. Negative pressure wound therapy for treating surgical wounds healing by secondary intention. Cochrane database Syst Rev [Internet]. 2015;(6):CD011278. Available from: http://ovidsp.ovid.com/ovidweb.cgi?T=JS&PAGE=reference&D=med12&NEWS=N&AN=26042534

813. Biter LU, Beck GMN, Mannaerts GHH, Stok MM, van der Ham AC, Grotenhuis BA. The use of negative-pressure wound therapy in pilonidal sinus disease: a randomized controlled trial comparing negative-pressure wound therapy versus standard open wound care after surgical excision. Dis Colon Rectum [Internet]. 2014;57(12):1406–11. Available from: http://ovidsp.ovid.com/ovidweb.cgi?T=JS&PAGE=reference&D=med11&NEWS=N&AN=25380007

814. Banasiewicz T, Bobkiewicz A, Borejsza-Wysocki M, Biczysko M, Ratajczak A, Malinger S, et al. Portable VAC therapy improve the results of the treatment of the pilonidal sinus--randomized prospective study. Pol Przegl Chir [Internet]. 2013;85(7):371–6. Available from: http://ovidsp.ovid.com/ovidweb.cgi?T=JS&PAGE=reference&D=med10&NEWS=N&AN=23945113

815. J. D, T. S. Use of negative pressure wound treatment PICO dressing for closure of pilonidal sinus disease. Color Dis [Internet]. 2019;21(Supplement 2):52. Available from: http://ovidsp.ovid.com/ovidweb.cgi?T=JS&PAGE=reference&D=emexb&NEWS=N&AN=631602603

816. Nakamichi M, Ogino A, Onishi K. Less invasive treatment for the pilonidal sinus combined use of negative-pressure wound therapy. Eur J Plast Surg [Internet]. 2020 Feb 2;43(1):75–8. Available from: http://link.springer.de/link/service/journals/00238/index.htm

817. E. B, T. A, S. S, C. Z. Negative pressure wound therapy is beneficial in the treatment of pilonidal disease. Surg Endosc [Internet]. 2019;33(Supplement 1):S272. Available from: http://ovidsp.ovid.com/ovidweb.cgi?T=JS&PAGE=reference&D=emexa&NEWS=N&AN=627143086

818. D. R, J. L, T. A, S. S, M. B. Negative pressure wound therapy is beneficial in the treatment of pilonidal disease with excision and primary closure. Dis Colon Rectum [Internet]. 2018;61(5):e305. Available from: http://ovidsp.ovid.com/ovidweb.cgi?T=JS&PAGE=reference&D=emed19&NEWS=N&AN=622082262

819. M. E. Our first attempt to use PICO after pilonidal cystectomy. Color Dis [Internet]. 2015;17(SUPPL. 2):92PIC. Available from: http://ovidsp.ovid.com/ovidweb.cgi?T=JS&PAGE=reference&D=emed16&NEWS=N&AN=72057742

820. Danne J, Gwini S, McKenzie D, Danne P. A Retrospective Study of Pilonidal Sinus Healing by Secondary Intention Using Negative Pressure Wound Therapy Versus Alginate or Gauze Dressings. Ostomy Wound Manage [Internet]. 2017;63(3):47–53. Available from: http://ovidsp.ovid.com/ovidweb.cgi?T=JS&PAGE=reference&D=med14&NEWS=N&AN=28355137

821. Farrell D, Murphy S. Negative pressure wound therapy for recurrent pilonidal disease: a review of the literature. J wound, ostomy, Cont Nurs Off Publ Wound, Ostomy Cont Nurses Soc [Internet]. 2011;38(4):373–8. Available from: http://ovidsp.ovid.com/ovidweb.cgi?T=JS&PAGE=reference&D=med8&NEWS=N&AN=21606863

822. Vaughn CJ, Lalikos JF. The use of acellular dermal regeneration template for recalcitrant pilonidal disease. J Wound Care [Internet]. 2011;20(6):275–7. Available from: http://ovidsp.ovid.com/ovidweb.cgi?T=JS&PAGE=reference&D=med8&NEWS=N&AN=21727876

823. Bendewald FP, Cima RR, Metcalf DR, Hassan I. Using negative pressure wound therapy following surgery for complex pilonidal disease: a case series. Ostomy Wound Manage [Internet]. 2007;53(5):40–6. Available from: http://ovidsp.ovid.com/ovidweb.cgi?T=JS&PAGE=reference&D=med6&NEWS=N&AN=17551174

824. Szmeja J, Borejsza-Wysocki M, Bobkiewicz A, Krokowicz L, Banasiewicz T, Szmyt K. Tu2053 THE COMPARISON OF QUALITY OF LIFE IN PATIENTS WITH PILONIDAL SINUS DISEASE. NEGATIVE PRESSURE WOUND THERAPY VERSUS STANDARD WOUND DRESSINGS - A RANDOMIZED PILOT STUDY. Gastroenterology [Internet]. 2020 May;158(6):S-1601. Available from: http://ovidsp.ovid.com/ovidweb.cgi?T=JS&PAGE=reference&D=emexb&NEWS=N&AN=2005914273

825. A. J. Use of PICO negative pressure wound therapy device in the management of patients with Pilonidal sinus. Color Dis [Internet]. 2017;19(Supplement 2):61–2. Available from: http://ovidsp.ovid.com/ovidweb.cgi?T=JS&PAGE=reference&D=emed18&NEWS=N&AN=618608616

826. Berry DP, Bale S, Harding KG. Dressings for treating cavity wounds. J Wound Care [Internet]. 1996;5(1):10–7. Available from: http://ovidsp.ovid.com/ovidweb.cgi?T=JS&PAGE=reference&D=med4&NEWS=N&AN=8697123

827. Walker AJ, Shouler PJ, Leicester RJ. Comparison between Eusol and Silastic foam dressing in the postoperative management of pilonidal sinus. J R Coll Surg Edinb [Internet]. 1991;36(2):105–6. Available from: http://ovidsp.ovid.com/ovidweb.cgi?T=JS&PAGE=reference&D=med3&NEWS=N&AN=2051404

828. Smith RC, Flynn PW, Gillett DJ, Guinness MD, Levey JM. Treatment of granulating wounds with silastic foam dressings. Aust N Z J Surg [Internet]. 1981;51(4):354–7. Available from: http://ovidsp.ovid.com/ovidweb.cgi?T=JS&PAGE=reference&D=med2&NEWS=N&AN=6944053

829. Wood RA, Hughes LE. Silicone foam sponge for pilonidal sinus: a new technique for dressing open granulating wounds. Br Med J [Internet]. 1975;4(5989):131–3. Available from: http://ovidsp.ovid.com/ovidweb.cgi?T=JS&PAGE=reference&D=med1&NEWS=N&AN=1191965

830. Koyuncu A, Karada H, Kurt A, Aydin C, Topcu O. Silver-impregnated dressings reduce wound closure time in marsupialized pilonidal sinus. EWMA J [Internet]. 2010 Sep;10(3):25–7. Available from: http://search.ebscohost.com/login.aspx?direct=true&db=cin20&AN=104946288&site=ehost-live

831. Sadati L, Froozesh R, Beyrami A, Khaneghah ZN, Elahi SA, Asl MF, et al. A Comparison of Three Dressing Methods for Pilonidal Sinus Surgery Wound Healing. Adv Skin Wound Care [Internet]. 2019;32(7):1–5. Available from: http://ovidsp.ovid.com/ovidweb.cgi?T=JS&PAGE=reference&D=medl&NEWS=N&AN=31232840

832. Romain B, Mielcarek M, Delhorme JB, Meyer N, Brigand C, Rohr S, et al. Dialkylcarbamoyl chloride‐coated versus alginate dressings after pilonidal sinus excision: a randomized clinical trial (SORKYSA study). BJS Open [Internet]. 2020 Apr;4(2):225–31. Available from: https://onlinelibrary.wiley.com/journal/24749842

833. Stevens J, Chaloner D. Urgosorb dressing: management of acute and chronic wounds. Br J Nurs [Internet]. 2005;14(15):S22-8. Available from: http://ovidsp.ovid.com/ovidweb.cgi?T=JS&PAGE=reference&D=med6&NEWS=N&AN=16144073

834. Viciano V, Castera JE, Medrano J, Aguilo J, Torro J, Botella MG, et al. Effect of hydrocolloid dressings on healing by second intention after excision of pilonidal sinus. Eur J Surg [Internet]. 2000;166(3):229–32. Available from: http://ovidsp.ovid.com/ovidweb.cgi?T=JS&PAGE=reference&D=med4&NEWS=N&AN=10755338

835. Estienne G, Di Bella F. [The use of DuoDerm in the surgical wound after surgical treatment of pilonidal fistulae using the open method]. L’impiego del “DuoDerm” nella ferita Oper dopo Tratt Chir delle fistole pilonidali col Metod aperto [Internet]. 1989;44(19):2089–92. Available from: http://ovidsp.ovid.com/ovidweb.cgi?T=JS&PAGE=reference&D=med3&NEWS=N&AN=2616009

836. H.A. K, N. O, O.F. E. The effect of hydrogel use on wound healing in pilonidal sinus patients undergoing surgical therapy by lay open technique. Turkish J Surg [Internet]. 2006;22(1):26–9. Available from: http://www.turkjsurg.com/

837. Marinovic M, Cicvaric T, Grzalja N, Bacic G, Radovic E. Application of wound dressing Molndal technique in clean and potentially contamined postoperative wounds--initial comparative study. Coll Antropol [Internet]. 2011;35 Suppl 2:103–6. Available from: http://ovidsp.ovid.com/ovidweb.cgi?T=JS&PAGE=reference&D=med8&NEWS=N&AN=22220414

838. H. B, B. M. Compare the effectiveness of using lyophilized collagen type i matrix with conventional treatment in open wound healing after surgery for pilonidal sinus. Nobel Med [Internet]. 2012;8(2):98–101. Available from: http://www.nobelmedicus.com/contents/201282/98-101.pdf

839. Aldemir M, Kara İH, Erten G, Taçyıldız İ. Effectiveness of Collagenase in the Treatment of Sacrococcygeal Pilonidal Sinus Disease. Surg Today [Internet]. 2003 Feb 1;33(2):106–9. Available from: http://ovidsp.ovid.com/ovidweb.cgi?T=JS&PAGE=reference&D=emed8&NEWS=N&AN=36269378

840. M. K, E. E. Bioresorbable woundscaffold (BWS) in pilonidal sinus wounds: A pilot study. Wound Repair Regen [Internet]. 2010;18(6):A85. Available from: http://ovidsp.ovid.com/ovidweb.cgi?T=JS&PAGE=reference&D=emed11&NEWS=N&AN=70483019

841. Mostafaei S, Norooznezhad F, Mohammadi S, Norooznezhad AH. Effectiveness of platelet-rich plasma therapy in wound healing of pilonidal sinus surgery: A comprehensive systematic review and meta-analysis. Wound Repair Regen [Internet]. 2017;25(6):1002–7. Available from: http://ovidsp.ovid.com/ovidweb.cgi?T=JS&PAGE=reference&D=med14&NEWS=N&AN=29215166

842. Mohammadi S, Nasiri S, Mohammadi MH, Malek Mohammadi A, Nikbakht M, Zahed Panah M, et al. Evaluation of platelet-rich plasma gel potential in acceleration of wound healing duration in patients underwent pilonidal sinus surgery: A randomized controlled parallel clinical trial. Transfus Apher Sci [Internet]. 2017;56(2):226–32. Available from: http://ovidsp.ovid.com/ovidweb.cgi?T=JS&PAGE=reference&D=med14&NEWS=N&AN=28119114

843. Achkasov EE, Ul’ianov AA, Bezuglov EN. [The use of autoplasma rich in platelet growth factors (APRPGF) on results of treatment of patients with pilonidal sinus abscess]. Khirurgiia (Sofiia) [Internet]. 2013;(12):43–7. Available from: http://ovidsp.ovid.com/ovidweb.cgi?T=JS&PAGE=reference&D=med10&NEWS=N&AN=24362291

844. Mavros MN, Mitsikostas PK, Alexiou VG, Peppas G, Falagas ME. Antimicrobials as an adjunct to pilonidal disease surgery: a systematic review of the literature. Eur J Clin Microbiol Infect Dis [Internet]. 2013 Jul 5;32(7):851–8. Available from: http://ovidsp.ovid.com/ovidweb.cgi?T=JS&PAGE=reference&D=emed14&NEWS=N&AN=52434260

845. Ypsilantis E, Carapeti E, Chan S. The use of topical 10% metronidazole in the treatment of non-healing pilonidal sinus wounds after surgery. Int J Colorectal Dis [Internet]. 2016;31(3):765–7. Available from: http://ovidsp.ovid.com/ovidweb.cgi?T=JS&PAGE=reference&D=med13&NEWS=N&AN=26003119

846. Hussain ZI, Aghahoseini A, Alexander D. Converting emergency pilonidal abscess into an elective procedure. Dis Colon Rectum [Internet]. 2012;55(6):640–5. Available from: http://ovidsp.ovid.com/ovidweb.cgi?T=JS&PAGE=reference&D=med9&NEWS=N&AN=22595842

847. Lasithiotakis K, Aghahoseini A, Volanaki D, Peter M, Alexander D. Aspiration for acute pilonidal abscess-a cohort study. J Surg Res [Internet]. 2018;223:123–7. Available from: http://ovidsp.ovid.com/ovidweb.cgi?T=JS&PAGE=reference&D=med15&NEWS=N&AN=29433863

848. Seker D, Ugurlu C, Ergul Z, Akinci M, Olcucuoglu E, Kulacoglu H. Single dose prophylactic antibiotics may not be sufficient in elective pilonidal sinus surgery: an early terminated study. Turkiye Klin J Med Sci [Internet]. 2011 Feb;31(1):186–90. Available from: http://search.ebscohost.com/login.aspx?direct=true&db=cin20&AN=104888982&site=ehost-live

849. E. K, L. A, L. P. Efficacy of topical 10% metronidazole in chronic nonhealing pilonidal incisions. Dis Colon Rectum [Internet]. 2014;57(5):e205--e206. Available from: http://ovidsp.ovid.com/ovidweb.cgi?T=JS&PAGE=reference&D=emed15&NEWS=N&AN=71493771

850. Z. H, A. A. Converting emergency pilonidal abscess into an elective procedure. Color Dis [Internet]. 2011;13(SUPPL. 5):27–8. Available from: http://ovidsp.ovid.com/ovidweb.cgi?T=JS&PAGE=reference&D=emed12&NEWS=N&AN=70566208

851. S. C, E. Y. The use of topical metronidazole in the treatment of nonhealing pilonidal sinus wounds. Color Dis [Internet]. 2014;16(SUPPL. 2):46. Available from: http://ovidsp.ovid.com/ovidweb.cgi?T=JS&PAGE=reference&D=emed15&NEWS=N&AN=71604216

852. GROUP SPS, Giannini I, Andreoli R, Bianchi FP, Cavallaro V, Corno F, et al. Effectiveness of topical use of Lietofix R in wound healing after pilonidalis sinus excision: a multicenter study by the Italian Society of Colorectal Surgery (SICCR). Tech Coloproctol [Internet]. 2019;23(4):373–8. Available from: http://ovidsp.ovid.com/ovidweb.cgi?T=JS&PAGE=reference&D=medl&NEWS=N&AN=30997607

853. Fernandez O, Capdevila JZ, Dalla G, Melchor G. Efficacy of Rhizophora mangle aqueous bark extract in the healing of open surgical wounds. Fitoterapia [Internet]. 2002;73(7–8):564–8. Available from: http://ovidsp.ovid.com/ovidweb.cgi?T=JS&PAGE=reference&D=med4&NEWS=N&AN=12490213

854. Agren MS, Ostenfeld U, Kallehave F, Gong Y, Raffn K, Crawford ME, et al. A randomized, double-blind, placebo-controlled multicenter trial evaluating topical zinc oxide for acute open wounds following pilonidal disease excision. Wound Repair Regen [Internet]. 2006;14(5):526–35. Available from: http://ovidsp.ovid.com/ovidweb.cgi?T=JS&PAGE=reference&D=med6&NEWS=N&AN=17014663

855. Pories WJ, Henzel JH, Rob CG, Strain WH. Acceleration of wound healing in man with zinc sulphate given by mouth. Lancet (London, England) [Internet]. 1967;1(7482):121–4. Available from: http://ovidsp.ovid.com/ovidweb.cgi?T=JS&PAGE=reference&D=med1&NEWS=N&AN=4163308

856. I. G, R. A, V. C, F. C, A. G, F. G, et al. Clinical trial on topical use of Lietofix in wound healing after pilonidal sinus excision: Preliminary data of a randomized, doubleblind, multicenter study of the Italian society of colorectal surgery (SICCR) (SICCR pilonidalis study group). Tech Coloproctol [Internet]. 2017;21(10):839. Available from: http://ovidsp.ovid.com/ovidweb.cgi?T=JS&PAGE=reference&D=emed18&NEWS=N&AN=619389668

857. M. L, U. F, P. M, K. K, S. B, L.B. J. The effect of a polyhexanide-betaine solution (Prontosan) on chronic persisting wounds after radical excision for pilonidal sinus disease. Color Dis [Internet]. 2017;19(Supplement 2):132. Available from: http://ovidsp.ovid.com/ovidweb.cgi?T=JS&PAGE=reference&D=emed18&NEWS=N&AN=618608143

858. V.O. G, S. D, E. E, V. C. Comparison of the efficiency of centella asiatica usage with the conventional treatment in the secondary wound healing after pilonidal sinus surgery. Eur Surg - Acta Chir Austriaca [Internet]. 2015;47(SUPPL. 1):S261. Available from: http://ovidsp.ovid.com/ovidweb.cgi?T=JS&PAGE=reference&D=emed16&NEWS=N&AN=71914048

859. J. G, N. D, A. M, P. S, S. O, A. M, et al. Zero percent recurrence rate following surgical treatment for chronic pilonidal disease under local anesthesia. Color Dis [Internet]. 2017;19(Supplement 2):127–8. Available from: http://ovidsp.ovid.com/ovidweb.cgi?T=JS&PAGE=reference&D=emed18&NEWS=N&AN=618607453

860. Haas S, Sørensen MJ, Lundby L, Pedersen AG. Injection of freshly collected autologous adipose tissue into non-healing wounds after closed incision pilonidal surgery. Tech Coloproctol [Internet]. 2020 Jul 9; Available from: http://link.springer.de/link/service/journals/10151/index.htm

861. M. R, S. S, M. M, P. O, C. FB. G-CSF-induced bone marrow-derived stem cells mobilization before sacrococcygeal pilonidal cyst surgery. Haematologica [Internet]. 2010;95(SUPPL. 2):692. Available from: http://www.haematologica.org/cgi/reprint/95/sup

862. Shirah BH, Shirah HA. Effect of surgical wound care methods of the lay open technique on the outcome of chronic sacrococcygeal pilonidal sinus management. Wound Med [Internet]. 2017 Mar;16:1–6. Available from: http://www.elsevier.com/journals/wound-medicine/2213-9095

863. Aksoy HM, Aksoy B, Egemen D. Effectiveness of topical use of natural polyphenols for the treatment of sacrococcygeal pilonidal sinus disease: a retrospective study including 192 patients. Eur J Dermatol [Internet]. 2010;20(4):476–81. Available from: http://ovidsp.ovid.com/ovidweb.cgi?T=JS&PAGE=reference&D=med8&NEWS=N&AN=20406728

864. Cairo SB, Zhao J, Ha M, Bass KD. Porcine bladder extracellular matrix in paediatric pilonidal wound care: healing and patient experience evaluation. J Wound Care [Internet]. 2019;28(Sup5):S12–9. Available from: http://ovidsp.ovid.com/ovidweb.cgi?T=JS&PAGE=reference&D=medl&NEWS=N&AN=31067171

865. Sibbald RG, Persaud Jaimangal R, Coutts PM, Elliott JA. Evaluating a Surfactant-Containing Polymeric Membrane Foam Wound Dressing with Glycerin in Patients with Chronic Pilonidal Sinus Disease. Adv Skin Wound Care [Internet]. 2018;31(7):298–305. Available from: http://ovidsp.ovid.com/ovidweb.cgi?T=JS&PAGE=reference&D=med15&NEWS=N&AN=29923900

866. Dorman RM, Bass KD. Novel use of porcine urinary bladder matrix for pediatric pilonidal wound care: preliminary experience. Pediatr Surg Int [Internet]. 2016;32(10):997–1002. Available from: http://ovidsp.ovid.com/ovidweb.cgi?T=JS&PAGE=reference&D=med13&NEWS=N&AN=27372297

867. FEIT HL. The use of thorium X in treatment of pilonidal cyst: a preliminary report. Dis Colon Rectum [Internet]. 1960;3:61–4. Available from: http://ovidsp.ovid.com/ovidweb.cgi?T=JS&PAGE=reference&D=med1&NEWS=N&AN=13821970

868. Mustafi N, Engels P. Post-surgical wound management of pilonidal cysts with a haemoglobin spray: a case series. J Wound Care [Internet]. 2016;25(4):191–8. Available from: http://ovidsp.ovid.com/ovidweb.cgi?T=JS&PAGE=reference&D=med13&NEWS=N&AN=27064368

869. Thomas M, Hamdan M, Hailes S, Walker M. Manuka honey as an effective treatment for chronic pilonidal sinus wounds. J Wound Care [Internet]. 2011;20(11):523–8. Available from: http://ovidsp.ovid.com/ovidweb.cgi?T=JS&PAGE=reference&D=med8&NEWS=N&AN=22240847

870. Ersoz F, Arikan S, Sari S, Korpinar S, Ozcan O, Poyraz B, et al. Effect of hyperbaric oxygen treatment on pilonidal disease surgery. Undersea Hyperb Med [Internet]. 2016;43(7):821–5. Available from: http://ovidsp.ovid.com/ovidweb.cgi?T=JS&PAGE=reference&D=med13&NEWS=N&AN=28777519

871. Kane FMA, Brodie EE, Coull A, Coyne L, Howd A, Milne A, et al. The analgesic effect of odour and music upon dressing change. Br J Nurs [Internet]. 2004;13(19):S4-12. Available from: http://ovidsp.ovid.com/ovidweb.cgi?T=JS&PAGE=reference&D=med5&NEWS=N&AN=15573017

872. Ozkan Z, Aksoy N, Emir S, Kanat BH, Gonen AN, Yazar FM, et al. Investigation of the relationship between serum hormones and pilonidal sinus disease: a cross-sectional study. Colorectal Dis [Internet]. 2014;16(4):311–4. Available from: http://ovidsp.ovid.com/ovidweb.cgi?T=JS&PAGE=reference&D=med11&NEWS=N&AN=24330514

873. Gikas A. Pilonidal sinus disease in young age is associated with diabetes later in life. J Diabetes [Internet]. 2014;6(1):90–1. Available from: http://ovidsp.ovid.com/ovidweb.cgi?T=JS&PAGE=reference&D=med11&NEWS=N&AN=23848540

874. Harlak A, Mentes O, Kilic S, Coskun K, Duman K, Yilmaz F. Sacrococcygeal pilonidal disease: analysis of previously proposed risk factors. Clinics (Sao Paulo) [Internet]. 2010;65(2):125–31. Available from: http://ovidsp.ovid.com/ovidweb.cgi?T=JS&PAGE=reference&D=med8&NEWS=N&AN=20186294

875. Cubukcu A, Carkman S, Gonullu NN, Alponat A, Kayabasi B, Eyuboglu E. Lack of evidence that obesity is a cause of pilonidal sinus disease. Eur J Surg [Internet]. 2001;167(4):297–8. Available from: http://ovidsp.ovid.com/ovidweb.cgi?T=JS&PAGE=reference&D=med4&NEWS=N&AN=11354323

876. Ardelt M, Dennler U, Fahrner R, Hallof G, Tautenhahn H-M, Dondorf F, et al. [Puberty is a major factor in pilonidal sinus disease : Gender-specific investigations of case number development in Germany from 2007 until 2015]. Die Pubertat ist ein wichtiger Fakt bei der Pilonidalsinuserkrankung Geschlechtsspezifische Untersuchung der Fallzahlentwicklung Deutschl 2007 bis 2015 [Internet]. 2017;88(11):961–7. Available from: http://ovidsp.ovid.com/ovidweb.cgi?T=JS&PAGE=reference&D=med14&NEWS=N&AN=28667368

877. Yildiz T, Elmas B, Yucak A, Turgut HT, Ilce Z. Risk Factors for Pilonidal Sinus Disease in Teenagers. Indian J Pediatr [Internet]. 2017;84(2):134–8. Available from: http://ovidsp.ovid.com/ovidweb.cgi?T=JS&PAGE=reference&D=med14&NEWS=N&AN=27306225

878. Milone M, Di Minno MN, Bianco P, Coretti G, Musella M, Milone F. Pilonidal sinus surgery: could we predict postoperative complications?. Int Wound J [Internet]. 2016;13(3):349–53. Available from: http://ovidsp.ovid.com/ovidweb.cgi?T=JS&PAGE=reference&D=med13&NEWS=N&AN=24894163

879. Sievert H, Evers T, Matevossian E, Hoenemann C, Hoffmann S, Doll D. The influence of lifestyle (smoking and body mass index) on wound healing and long-term recurrence rate in 534 primary pilonidal sinus patients. Int J Colorectal Dis [Internet]. 2013;28(11):1555–62. Available from: http://ovidsp.ovid.com/ovidweb.cgi?T=JS&PAGE=reference&D=med10&NEWS=N&AN=23780586

880. Chijiwa T, Suganuma T, Takigawa T, Edogawa S, Inoue K, Yanagida S, et al. Pilonidal sinus in Japan maritime self-defense force at Yokosuka. Mil Med [Internet]. 2006;171(7):650–2. Available from: http://ovidsp.ovid.com/ovidweb.cgi?T=JS&PAGE=reference&D=med6&NEWS=N&AN=16895134

881. Arda IS, Guney LH, Sevmis S, Hicsonmez A. High body mass index as a possible risk factor for pilonidal sinus disease in adolescents. World J Surg [Internet]. 2005;29(4):469–71. Available from: http://ovidsp.ovid.com/ovidweb.cgi?T=JS&PAGE=reference&D=med6&NEWS=N&AN=15770382

882. Sondenaa K, Andersen E, Nesvik I, Soreide JA. Patient characteristics and symptoms in chronic pilonidal sinus disease. Int J Colorectal Dis [Internet]. 1995;10(1):39–42. Available from: http://ovidsp.ovid.com/ovidweb.cgi?T=JS&PAGE=reference&D=med3&NEWS=N&AN=7745322

883. Doll D, Friederichs J, Dettmann H, Boulesteix A-L, Duesel W, Petersen S. Time and rate of sinus formation in pilonidal sinus disease. Int J Colorectal Dis [Internet]. 2008;23(4):359–64. Available from: http://ovidsp.ovid.com/ovidweb.cgi?T=JS&PAGE=reference&D=med7&NEWS=N&AN=18043929

884. Almajid FM, Alabdrabalnabi AA, Almulhim KA. The risk of recurrence of Pilonidal disease after surgical management. Saudi Med J [Internet]. 2017;38(1):70–4. Available from: http://ovidsp.ovid.com/ovidweb.cgi?T=JS&PAGE=reference&D=med14&NEWS=N&AN=28042633

885. Bolandparvaz S, Moghadam Dizaj P, Salahi R, Paydar S, Bananzadeh M, Abbasi HR, et al. Evaluation of the risk factors of pilonidal sinus: A single center experience. Turkish J Gastroenterol. 2012;

886. Faraj FH, Baba HO, Salih AM, Kakamad FH. Risk factors of pilonidal sinus disease in preparatory school students; a case control study. Ann Med Surg [Internet]. 2020 Sep;57:46–8. Available from: http://www.elsevier.com/journals/annals-of-medicine-and-surgery/2049-0801

887. Ince M, Atilgan H, Ozcelik F, Arslan E. Smoking and Lipid Profile in Pilonidal Sinus Patients. Eur J Gen Med [Internet]. 2014 Jun;11(2):90–3. Available from: http://search.ebscohost.com/login.aspx?direct=true&db=cin20&AN=103971686&site=ehost-live

888. Kuvvetli A, Çetinkunar S, Parlakgümüş A. Evaluation of Etiological Risk Factors in the Development of Adult Chronic Pilonidal Disease. Turkish J Color Dis [Internet]. 2019 Jun 1;29(2):75–7. Available from: http://search.ebscohost.com/login.aspx?direct=true&db=cin20&AN=136795167&site=ehost-live

889. R. E, M. S, O. A. Predisposing factors in chronic pilonidal sinus development. Turkish J Surg [Internet]. 2003;19(1):49–53. Available from: http://www.turkjsurg.com/

890. Yoldas T, Karaca C, Unalp O, Uguz A, Caliskan C, Akgun E, et al. Recurrent pilonidal sinus: lay open or flap closure, does it differ?. Int Surg [Internet]. 2013;98(4):319–23. Available from: http://ovidsp.ovid.com/ovidweb.cgi?T=JS&PAGE=reference&D=med10&NEWS=N&AN=24229017

891. Iesalnieks I, Deimel S, Zulke C, Schlitt HJ. Smoking increases the risk of pre- and postoperative complications in patients with pilonidal disease. J Dtsch Dermatol Ges [Internet]. 2013;11(10):1001–5. Available from: http://ovidsp.ovid.com/ovidweb.cgi?T=JS&PAGE=reference&D=med10&NEWS=N&AN=23945165

892. Doll D, Matevossian E, Wietelmann K, Evers T, Kriner M, Petersen S. Family history of pilonidal sinus predisposes to earlier onset of disease and a 50% long-term recurrence rate. Dis Colon Rectum [Internet]. 2009;52(9):1610–5. Available from: http://ovidsp.ovid.com/ovidweb.cgi?T=JS&PAGE=reference&D=med7&NEWS=N&AN=19690490

893. Clothier PR, Haywood IR. The natural history of the post anal (pilonidal) sinus. Ann R Coll Surg Engl [Internet]. 1984;66(3):201–3. Available from: http://ovidsp.ovid.com/ovidweb.cgi?T=JS&PAGE=reference&D=med2&NEWS=N&AN=6721409

894. Kasim K, Abdlhamid NM, Badwan BR, Allowbany A. Is There a Relation Between Natal Cleft Depth and Post-Operative Morbidity After Different Methods of Excision of Sacro-Coccygeal Pilonidal Sinus? Indian J Surg [Internet]. 2015 Dec 29;77(S2):201–5. Available from: http://search.ebscohost.com/login.aspx?direct=true&db=cin20&AN=111984593&site=ehost-live

895. Ferhatoglu MF, Kartal A, Ekici U, Kebudi A. Effects of Bathing Habits on Postoperative Wound Complications Following Sacrococcygeal Pilonidal Sinus Surgery: A Retrospective Analysis of 67 Adolescent Patients. Wounds a Compend Clin Res Pract [Internet]. 2019;31(11):292–6. Available from: http://ovidsp.ovid.com/ovidweb.cgi?T=JS&PAGE=reference&D=medl&NEWS=N&AN=31747369

896. M.F. F, U. E. Retrospective analysis of bathing habits of 67 adolescent pilonidal sinus disease cases. Color Dis [Internet]. 2018;20(Supplement 4):132. Available from: http://ovidsp.ovid.com/ovidweb.cgi?T=JS&PAGE=reference&D=emed19&NEWS=N&AN=624186071

897. Doll D, Bosche FD, Stauffer VK, Sinicina I, Hoffmann S, van der Zypen D, et al. Strength of Occipital Hair as an Explanation for Pilonidal Sinus Disease Caused by Intruding Hair. Dis Colon Rectum [Internet]. 2017;60(9):979–86. Available from: http://ovidsp.ovid.com/ovidweb.cgi?T=JS&PAGE=reference&D=med14&NEWS=N&AN=28796737

898. Doll D, Bosche F, Hauser A, Moersdorf P, Sinicina I, Grunwald J, et al. The presence of occipital hair in the pilonidal sinus cavity-a triple approach to proof. Int J Colorectal Dis [Internet]. 2018;33(5):567–76. Available from: http://ovidsp.ovid.com/ovidweb.cgi?T=JS&PAGE=reference&D=med15&NEWS=N&AN=29488088

899. Bosche F, Luedi MM, van der Zypen D, Moersdorf P, Krapohl B, Doll D. The Hair in the Sinus: Sharp-Ended Rootless Head Hair Fragments can be Found in Large Amounts in Pilonidal Sinus Nests. World J Surg [Internet]. 2018;42(2):567–73. Available from: http://ovidsp.ovid.com/ovidweb.cgi?T=JS&PAGE=reference&D=med15&NEWS=N&AN=28639004

900. Sondenaa K, Pollard ML. Histology of chronic pilonidal sinus. APMIS [Internet]. 1995;103(4):267–72. Available from: http://ovidsp.ovid.com/ovidweb.cgi?T=JS&PAGE=reference&D=med3&NEWS=N&AN=7542011

901. FRANCKOWIAK JJ, JACKMAN RJ. The etiology of pilonidal sinus. Dis Colon Rectum [Internet]. 1962;5:28–36. Available from: http://ovidsp.ovid.com/ovidweb.cgi?T=JS&PAGE=reference&D=med1&NEWS=N&AN=13894530

902. Karakus E, Kacar A, Karakus R, Mambet E, Senayli A. Expression of Epstein-Barr virus in children with sacrococcygeal pilonidal sinus determined by immunohistochemical methods. Int Wound J [Internet]. 2016;13(2):265–7. Available from: http://ovidsp.ovid.com/ovidweb.cgi?T=JS&PAGE=reference&D=med13&NEWS=N&AN=24758314

903. Ardelt M, Dittmar Y, Kocijan R, Rodel J, Schulz B, Scheuerlein H, et al. Microbiology of the infected recurrent sacrococcygeal pilonidal sinus. Int Wound J [Internet]. 2016;13(2):231–7. Available from: http://ovidsp.ovid.com/ovidweb.cgi?T=JS&PAGE=reference&D=med13&NEWS=N&AN=24758263

904. Russano de Paiva G, Adolfo da Silva NJ, March M, Brousset P, Laurent C. High frequency of Epstein-Barr virus-infected lymphocytes in pilonidal cysts. Hum Pathol [Internet]. 2012;43(12):2241–6. Available from: http://ovidsp.ovid.com/ovidweb.cgi?T=JS&PAGE=reference&D=med9&NEWS=N&AN=22748471

905. Brook I, Anderson KD, Controni G, Rodriguez WJ. Aerobic and anaerobic bacteriology of pilonidal cyst abscess in children. Am J Dis Child [Internet]. 1980;134(7):679–80. Available from: http://ovidsp.ovid.com/ovidweb.cgi?T=JS&PAGE=reference&D=med2&NEWS=N&AN=7395830

906. K. S, I. N, E. A, M.L. P. Recurrent pilonidal sinus: Etiology and treatment. Dig Surg [Internet]. 1995;12(2):117–20. Available from: http://ovidsp.ovid.com/ovidweb.cgi?T=JS&PAGE=reference&D=emed5&NEWS=N&AN=25264702

907. Sahin S. Endogenous Thymosin beta4 Expression in Sacrococcygeal Pilonidal Sinus Disease: A Retrospective, Immunohistochemical Analysis of Excisional Skin Biopsy Samples. Ostomy Wound Manage [Internet]. 2017;63(4):30–40. Available from: http://ovidsp.ovid.com/ovidweb.cgi?T=JS&PAGE=reference&D=med14&NEWS=N&AN=28448267

908. Hopkinson I, Evans W, Chant D, Hiscox S, Berry D, Harding K. Reverse transcription-polymerase chain reaction detection of collagen transcripts in healing human wounds. Eur J Clin Invest [Internet]. 1995;25(7):539–42. Available from: http://ovidsp.ovid.com/ovidweb.cgi?T=JS&PAGE=reference&D=med3&NEWS=N&AN=7556374

909. Fukumura Y, Takase M, Mitani K, Suda K, Imamhasan A, Nobukawa B, et al. Amount of CD4+CD25+ regulatory T cells in autoimmune pancreatitis and pilonidal sinus. Pancreas [Internet]. 2012;41(6):910–5. Available from: http://ovidsp.ovid.com/ovidweb.cgi?T=JS&PAGE=reference&D=med9&NEWS=N&AN=22466163

910. von Laffert M, Stadie V, Ulrich J, Marsch WC, Wohlrab J. Morphology of pilonidal sinus disease: some evidence of its being a unilocalized type of hidradenitis suppurativa. Dermatology [Internet]. 2011;223(4):349–55. Available from: http://ovidsp.ovid.com/ovidweb.cgi?T=JS&PAGE=reference&D=med8&NEWS=N&AN=22269798

911. Kurokawa I, Nishijima S, Suzuki K, Kusumoto K, Sensaki H, Shikata N, et al. Cytokeratin expression in pilonidal sinus. Br J Dermatol [Internet]. 2002;146(3):409–13. Available from: http://ovidsp.ovid.com/ovidweb.cgi?T=JS&PAGE=reference&D=med4&NEWS=N&AN=11952540

912. Boyce DE, Jones WD, Ruge F, Harding KG, Moore K. The role of lymphocytes in human dermal wound healing. Br J Dermatol [Internet]. 2000;143(1):59–65. Available from: http://ovidsp.ovid.com/ovidweb.cgi?T=JS&PAGE=reference&D=med4&NEWS=N&AN=10886136

913. Doll D, Friederichs J, Boulesteix A-L, Dusel W, Fend F, Petersen S. Surgery for asymptomatic pilonidal sinus disease. Int J Colorectal Dis [Internet]. 2008;23(9):839–44. Available from: http://ovidsp.ovid.com/ovidweb.cgi?T=JS&PAGE=reference&D=med7&NEWS=N&AN=18491116

914. H. OH, V. N. Pilonidal sinus: Histological course of the healing process and its relationship with the treatment. Cir Esp [Internet]. 1979;33(2):137–42. Available from: http://ovidsp.ovid.com/ovidweb.cgi?T=JS&PAGE=reference&D=emed2&NEWS=N&AN=9216166

915. Garg P. Anal fistula and pilonidal sinus disease coexisting simultaneously: An audit in a cohort of 1284 patients. Int Wound J [Internet]. 2019;16(5):1199–205. Available from: http://ovidsp.ovid.com/ovidweb.cgi?T=JS&PAGE=reference&D=medl&NEWS=N&AN=31412425

916. Soy M, Atagunduz P, Bes C, Aksoy A, Kocakaya O. High prevalence of sacrococcygeal pilonidal sinus disease in patients with ankylosing spondylitis. Jt bone spine [Internet]. 2018;85(4):505–6. Available from: http://ovidsp.ovid.com/ovidweb.cgi?T=JS&PAGE=reference&D=medc&NEWS=N&AN=29032249

917. Binnebosel M, Junge K, Schwab R, Antony A, Schumpelick V, Klinge U. Delayed wound healing in sacrococcygeal pilonidal sinus coincides with an altered collagen composition. World J Surg [Internet]. 2009;33(1):130–7. Available from: http://ovidsp.ovid.com/ovidweb.cgi?T=JS&PAGE=reference&D=med7&NEWS=N&AN=18839241

918. P. G. Patient with hidradenitis suppurativa (HS)-associated pilonidal sinus disease (PSD) are different from patients with HS-free PSD. Exp Dermatol [Internet]. 2017;26(Supplement 1):33–4. Available from: http://ovidsp.ovid.com/ovidweb.cgi?T=JS&PAGE=reference&D=emed18&NEWS=N&AN=614602491

919. Eryilmaz R, Isik A, Okan I, Bilecik T, Yekeler E, Sahin M. Does Sacrococcygeal Angle Play a Role on Pilonidal Sinus Etiology?. Prague Med Rep [Internet]. 2015;116(3):219–24. Available from: http://ovidsp.ovid.com/ovidweb.cgi?T=JS&PAGE=reference&D=med12&NEWS=N&AN=26445393

920. Akinci OF, Kurt M, Terzi A, Atak I, Subasi IE, Akbilgic O. Natal cleft deeper in patients with pilonidal sinus: implications for choice of surgical procedure. Dis Colon Rectum [Internet]. 2009;52(5):1000–2. Available from: http://ovidsp.ovid.com/ovidweb.cgi?T=JS&PAGE=reference&D=med7&NEWS=N&AN=19502869

921. Balik O, Balik AA, Polat KY, Aydinli B, Kantarci M, Aliagaoglu C, et al. The importance of local subcutaneous fat thickness in pilonidal disease. Dis Colon Rectum [Internet]. 2006;49(11):1755–7. Available from: http://ovidsp.ovid.com/ovidweb.cgi?T=JS&PAGE=reference&D=med6&NEWS=N&AN=17036206

922. SEBRECHTS PH. A significant diagnostic sign of pilonidal disease. Dis Colon Rectum [Internet]. 1961;4:56–9. Available from: http://ovidsp.ovid.com/ovidweb.cgi?T=JS&PAGE=reference&D=med1&NEWS=N&AN=13749539

923. Kaplan M, Ozturk S, Cakin H, Akgun B, Onur MR, Erol FS. Sacrococcygeal sinus angle: as a new anatomic landmark for the posterior approach of presacral lesions. Eur Spine J [Internet]. 2014 Feb 17;23(2):337–40. Available from: http://link.springer.de/link/service/journals/00586/index.htm

924. Duman K, Girgin M, Harlak A. Prevalence of sacrococcygeal pilonidal disease in Turkey. Asian J Surg [Internet]. 2017;40(6):434–7. Available from: http://ovidsp.ovid.com/ovidweb.cgi?T=JS&PAGE=reference&D=med14&NEWS=N&AN=27188235

925. (AFHSC) AFHSC. Pilonidal cysts, active component, U.S. Armed Forces, 2000-2012. MSMR [Internet]. 2013;20(12):8–11. Available from: http://ovidsp.ovid.com/ovidweb.cgi?T=JS&PAGE=reference&D=med10&NEWS=N&AN=24428537

926. Aysan E, Ilhan M, Bektas H, Kaya EA, Sam B, Buyukpinarbasili N, et al. Prevalence of sacrococcygeal pilonidal sinus as a silent disease. Surg Today [Internet]. 2013;43(11):1286–9. Available from: http://ovidsp.ovid.com/ovidweb.cgi?T=JS&PAGE=reference&D=med10&NEWS=N&AN=23184326

927. Evers T, Doll D, Matevossian E, Noe S, Neumann K, Li H, et al. [Trends in incidence and long-term recurrence rate of pilonidal sinus disease and analysis of associated influencing factors]. Zhonghua Wai Ke Za Zhi [Internet]. 2011;49(9):799–803. Available from: http://ovidsp.ovid.com/ovidweb.cgi?T=JS&PAGE=reference&D=med8&NEWS=N&AN=22177433

928. Akinci OF, Bozer M, Uzunkoy A, Duzgun SA, Coskun A. Incidence and aetiological factors in pilonidal sinus among Turkish soldiers. Eur J Surg [Internet]. 1999;165(4):339–42. Available from: http://ovidsp.ovid.com/ovidweb.cgi?T=JS&PAGE=reference&D=med4&NEWS=N&AN=10365835

929. Chamberlain JW, Vawter GF. The congenital origin of pilonidal sinus. J Pediatr Surg [Internet]. 1974;9(4):441–4. Available from: http://ovidsp.ovid.com/ovidweb.cgi?T=JS&PAGE=reference&D=med1&NEWS=N&AN=4846299

930. Benhadou F, Van der Zee HH, Pascual JC, Rigopoulos D, Katoulis A, Liakou AI, et al. Pilonidal sinus disease: an intergluteal localization of hidradenitis suppurativa/acne inversa: a cross‐sectional study among 2465 patients. Br J Dermatol [Internet]. 2019 Dec 6;181(6):1198–206. Available from: http://onlinelibrary.wiley.com/journal/10.1111/(ISSN)1365-2133

931. Gaiser MR, Lee SB, Enk A, Schrott P, Weisser H. Surgical intervention of pilonidal sinus: impact on patients’ postoperative satisfaction and return to work time. Eur J Dermatol [Internet]. 2013;23(4):487–90. Available from: http://ovidsp.ovid.com/ovidweb.cgi?T=JS&PAGE=reference&D=med10&NEWS=N&AN=23816710

932. Pronk A, Kastelijns L, Smakman N, Furnee E. Sexual Function in Patients Suffering from Sacrococcygeal Pilonidal Sinus Disease. Cureus [Internet]. 2020 Mar 2;12(3):e7159. Available from: http://ovidsp.ovid.com/ovidweb.cgi?T=JS&PAGE=reference&D=emed19&NEWS=N&AN=624186431

933. Stewart AM, Baker JD, Elliott D. The effects of a sacrococcygeal pilonidal sinus wound on activities of living: thematic analysis of participant interviews. J Clin Nurs [Internet]. 2011;20(21–22):3174–82. Available from: http://ovidsp.ovid.com/ovidweb.cgi?T=JS&PAGE=reference&D=med8&NEWS=N&AN=21831106

934. McCaughan D, Sheard L, Cullum N, Dumville J, Chetter I. Nurses’ and surgeons’ views and experiences of surgical wounds healing by secondary intention: A qualitative study. J Clin Nurs. 2020;

935. Bradley L. Pilonidal sinus disease: a review. Part two. J Wound Care [Internet]. 2010;19(12):522–30. Available from: http://ovidsp.ovid.com/ovidweb.cgi?T=JS&PAGE=reference&D=med8&NEWS=N&AN=21160443

936. N. S, O. A. Patient expectations and patient reported outcome of pilonidal disease treatment. Color Dis [Internet]. 2015;17(SUPPL. 2):98. Available from: http://ovidsp.ovid.com/ovidweb.cgi?T=JS&PAGE=reference&D=emed16&NEWS=N&AN=72057781

937. Zwarenstein M, Shariff S, Mittmann N, Stern A, Dainty KN. A large cluster randomized trial of outcome-based pathways to improve home-based wound care. Trials [Internet]. 2017;18(1):393. Available from: http://ovidsp.ovid.com/ovidweb.cgi?T=JS&PAGE=reference&D=med14&NEWS=N&AN=28851413

938. Augestad KM, Revhaug A, Vonen B, Johnsen R, Lindsetmo R-O. The one-stop trial: does electronic referral and booking by the general practitioner (GPs) to outpatient day case surgery reduce waiting time and costs? A randomized controlled trial protocol. BMC Surg [Internet]. 2008;8:14. Available from: http://ovidsp.ovid.com/ovidweb.cgi?T=JS&PAGE=reference&D=med7&NEWS=N&AN=18694477

939. Harris C, Sibbald RG, Mufti A, Somayaji R. Pilonidal Sinus Disease: 10 Steps to Optimize Care. Adv Skin Wound Care [Internet]. 2016;29(10):469–78. Available from: http://ovidsp.ovid.com/ovidweb.cgi?T=JS&PAGE=reference&D=med13&NEWS=N&AN=27632444

940. Doll D, Krueger CM, Schrank S, Dettmann H, Petersen S, Duesel W. Timeline of recurrence after primary and secondary pilonidal sinus surgery. Dis Colon Rectum [Internet]. 2007;50(11):1928–34. Available from: http://ovidsp.ovid.com/ovidweb.cgi?T=JS&PAGE=reference&D=med6&NEWS=N&AN=17874268

941. Gümüşoğlu AY, Ertürk S. The Effects of Pilonidal Sinus Morphology on Surgical Selection. Bakirkoy Tip Derg / Med J Bakirkoy [Internet]. 2019 Sep 1;15(3):259–64. Available from: http://search.ebscohost.com/login.aspx?direct=true&db=cin20&AN=138812621&site=ehost-live

942. T. T. Pilonidal sinus: Pathology reports. Tech Coloproctol [Internet]. 2014;18(4):416. Available from: http://ovidsp.ovid.com/ovidweb.cgi?T=JS&PAGE=reference&D=emed15&NEWS=N&AN=71437569

943. Ki-Shing Miu V, Tang JYM, Matteucci P. Plastic surgeons &amp; pilonidal disease – The East Yorkshire experience. Int J Surg [Internet]. 2013 Oct;11(8):684. Available from: http://ovidsp.ovid.com/ovidweb.cgi?T=JS&PAGE=reference&D=emed14&NEWS=N&AN=71272050

944. Begaj A. An audit on post-surgical complication rates of elective excision of pilonidal sinus disease and the need of post-operative appointments. Int J Surg [Internet]. 2016 Nov;36(Supplement 1):S100. Available from: http://ovidsp.ovid.com/ovidweb.cgi?T=JS&PAGE=reference&D=emed17&NEWS=N&AN=619296178

945. Lamdark T, Vuille-dit-Bille RN, Bielicki IN, Guglielmetti LC, Choudhury RA, Peters N, et al. Treatment Strategies for Pilonidal Sinus Disease in Switzerland and Austria. Medicina (B Aires) [Internet]. 2020 Jul 9;56(7):341. Available from: http://ovidsp.ovid.com/ovidweb.cgi?T=JS&PAGE=reference&D=emexb&NEWS=N&AN=632358500

946. Fabricius R, Petersen LW, Bertelsen CA. Treatment of pilonidal sinuses in Denmark is not optimal. Dan Med Bull [Internet]. 2010;57(12):A4200. Available from: http://ovidsp.ovid.com/ovidweb.cgi?T=JS&PAGE=reference&D=med8&NEWS=N&AN=21122458

947. Beal EM, Lee MJ, Hind D, Wysocki AP, Yang F, Brown SR. A systematic review of classification systems for pilonidal sinus. Tech Coloproctol [Internet]. 2019;23(5):435–43. Available from: http://ovidsp.ovid.com/ovidweb.cgi?T=JS&PAGE=reference&D=medc&NEWS=N&AN=31098861

948. Guner A, Cekic AB, Boz A, Turkyilmaz S, Kucuktulu U. A proposed staging system for chronic symptomatic pilonidal sinus disease and results in patients treated with stage-based approach. BMC Surg [Internet]. 2016;16:18. Available from: http://ovidsp.ovid.com/ovidweb.cgi?T=JS&PAGE=reference&D=med13&NEWS=N&AN=27084534

949. Schreckenbach T, El Youzouri H, Bechstein WO, Habbe N. Proctologic surgery done by residents - Complications preprogrammed?. J Visc Surg [Internet]. 2016;153(3):167–72. Available from: http://ovidsp.ovid.com/ovidweb.cgi?T=JS&PAGE=reference&D=med13&NEWS=N&AN=26822665

950. Milone M, Musella M, Maietta P, Bianco P, Taffuri C, Salvatore G, et al. Outpatient surgical procedures: which is the ideal teaching procedure for a resident surgeon?. G Chir [Internet]. 2013;34(11–12):311–4. Available from: http://ovidsp.ovid.com/ovidweb.cgi?T=JS&PAGE=reference&D=med10&NEWS=N&AN=24342157

951. Wortsman X, Castro A, Morales C, Franco C, Figueroa A. Sonographic Comparison of Morphologic Characteristics Between Pilonidal Cysts and Hidradenitis Suppurativa. J Ultrasound Med [Internet]. 2017;36(12):2403–18. Available from: http://ovidsp.ovid.com/ovidweb.cgi?T=JS&PAGE=reference&D=med14&NEWS=N&AN=28649748

952. Craven T, Webster PJ, Burke DA. Microbiological swabs have no role in the management of acute pilonidal abscesses. Int J Surg [Internet]. 2016 Nov;36(Supplement 1):S36–7. Available from: http://ovidsp.ovid.com/ovidweb.cgi?T=JS&PAGE=reference&D=emed17&NEWS=N&AN=619296406

953. Karakaş DÖ. Role of Ultrasonography in Evaluation of Pilonidal Disease. Turkish J Color Dis [Internet]. 2018 Dec 31;28(4):182–5. Available from: http://search.ebscohost.com/login.aspx?direct=true&db=cin20&AN=134177407&site=ehost-live

954. Taylor SA, Halligan S, Bartram CI. Pilonidal sinus disease: MR imaging distinction from fistula in ano. Radiology [Internet]. 2003;226(3):662–7. Available from: http://ovidsp.ovid.com/ovidweb.cgi?T=JS&PAGE=reference&D=med5&NEWS=N&AN=12601210

955. Yuksel ME, Tamer F. All pilonidal sinus surgery specimens should be histopathologically evaluated in order to rule out malignancy. J Visc Surg [Internet]. 2019;156(5):469–70. Available from: http://ovidsp.ovid.com/ovidweb.cgi?T=JS&PAGE=reference&D=medl&NEWS=N&AN=31085138

956. Wortsman X, Wortsman J. Clinical usefulness of variable-frequency ultrasound in localized lesions of the skin. J Am Acad Dermatol [Internet]. 2010;62(2):247–56. Available from: http://ovidsp.ovid.com/ovidweb.cgi?T=JS&PAGE=reference&D=med8&NEWS=N&AN=19962214

957. Maeda T, Yanagi T, Imafuku K, Kitamura S, Hata H, Shimizu H. Photodynamic eye precisely reveals pilonidal sinus borders. Int J Dermatol [Internet]. 2017;56(12):1514–5. Available from: http://ovidsp.ovid.com/ovidweb.cgi?T=JS&PAGE=reference&D=med14&NEWS=N&AN=28960273

958. Solivetti FM, Elia F, Panetta C, Teoli M, Bucher S, Di Carlo A. Preoperative advantages of HF sonography of pilonidal sinus. G Ital Dermatol Venereol [Internet]. 2012;147(4):407–11. Available from: http://ovidsp.ovid.com/ovidweb.cgi?T=JS&PAGE=reference&D=med9&NEWS=N&AN=23007215

959. Avni EF, Matos C, Grassart A, Christophe C, Pardou A, Baleriaux D. [Neonatal pilonidal sinuses and screening by medullary ultrasonography: preliminary results]. Sinus pilonidaux neonatals Echogr medullaire Depist Result Prelim [Internet]. 1991;46(8–9):607–11. Available from: http://ovidsp.ovid.com/ovidweb.cgi?T=JS&PAGE=reference&D=med3&NEWS=N&AN=1660120

960. Brook I. Microbiology of infected pilonidal sinuses. J Clin Pathol [Internet]. 1989;42(11):1140–2. Available from: http://ovidsp.ovid.com/ovidweb.cgi?T=JS&PAGE=reference&D=med3&NEWS=N&AN=2584424

961. Youssef AT. The value of superficial parts and endoanal ultrasonography in evaluating pilonidal disease and exclusion of perianal sepsis. J Ultrasound [Internet]. 2015 Sep 17;18(3):237–43. Available from: http://www.springer.com/medicine/radiology/journal/40477

962. Schmittner MD, Dieterich S, Gebhardt V, Weiss C, Burmeister MA, Bussen DG, et al. Randomised clinical trial of pilonidal sinus operations performed in the prone position under spinal anaesthesia with hyperbaric bupivacaine 0.5 % versus total intravenous anaesthesia. Int J Colorectal Dis [Internet]. 2013;28(6):873–80. Available from: http://ovidsp.ovid.com/ovidweb.cgi?T=JS&PAGE=reference&D=med10&NEWS=N&AN=23196892

963. Cuvas O, Gulec H, Karaaslan M, Basar H. The use of low dose plain solutions of local anaesthetic agents for spinal anaesthesia in the prone position: bupivacaine compared with levobupivacaine. Anaesthesia [Internet]. 2009;64(1):14–8. Available from: http://ovidsp.ovid.com/ovidweb.cgi?T=JS&PAGE=reference&D=med7&NEWS=N&AN=19087000

964. Terzi C, Canda AE, Unek T, Dalgic E, Fuzun M. What is the role of mechanical bowel preparation in patients with pilonidal sinus undergoing surgery? Prospective, randomized, surgeon-blinded trial. World J Surg [Internet]. 2005;29(11):1465–71. Available from: http://ovidsp.ovid.com/ovidweb.cgi?T=JS&PAGE=reference&D=med6&NEWS=N&AN=16240065

965. Aysan E, Basak F, Kinaci E, Sevinc M. Efficacy of local adrenalin injection during sacrococcygeal pilonidal sinus excision. Eur Surg Res [Internet]. 2004;36(4):256–8. Available from: http://ovidsp.ovid.com/ovidweb.cgi?T=JS&PAGE=reference&D=med5&NEWS=N&AN=15263832

966. Naja MZ, Ziade MF, El Rajab M. Sacrococcygeal local anaesthesia versus general anaesthesia for pilonidal sinus surgery: a prospective randomised trial. Anaesthesia [Internet]. 2003;58(10):1007–12. Available from: http://ovidsp.ovid.com/ovidweb.cgi?T=JS&PAGE=reference&D=med5&NEWS=N&AN=12969043

967. Sungurtekin H, Sungurtekin U, Erdem E. Local anesthesia and midazolam versus spinal anesthesia in ambulatory pilonidal surgery. J Clin Anesth [Internet]. 2003;15(3):201–5. Available from: http://ovidsp.ovid.com/ovidweb.cgi?T=JS&PAGE=reference&D=med5&NEWS=N&AN=12770656

968. M.D. S, S. D, C. W, D. B. Randomized clinical trial of pilonidal sinus operations performed in the prone position under spinal saddle block versus total intravenous anaesthesia. Eur J Anaesthesiol [Internet]. 2011;28(SUPPL. 48):24. Available from: http://ovidsp.ovid.com/ovidweb.cgi?T=JS&PAGE=reference&D=emed12&NEWS=N&AN=70681044

969. Luedi MM, Kauf P, Evers T, Sievert H, Doll D. Impact of spinal versus general anesthesia on postoperative pain and long term recurrence after surgery for pilonidal disease. J Clin Anesth [Internet]. 2016;33:236–42. Available from: http://ovidsp.ovid.com/ovidweb.cgi?T=JS&PAGE=reference&D=med13&NEWS=N&AN=27555172

970. Bertelsen CA. Cleft-lift operation for pilonidal sinuses under tumescent local anesthesia: a prospective cohort study of peri- and postoperative pain. Dis Colon Rectum [Internet]. 2011;54(7):895–900. Available from: http://ovidsp.ovid.com/ovidweb.cgi?T=JS&PAGE=reference&D=med8&NEWS=N&AN=21654258

971. Biffoni M, Scipioni P, Macrina N, Amabile MI, Garritano S, Maturo A, et al. [Pilonidal sinus. Outpatient treatment with local anesthesia]. Sinus pilonidalis Tratt ambulatoriale Anest locale [Internet]. 2009;30(4):173–6. Available from: http://ovidsp.ovid.com/ovidweb.cgi?T=JS&PAGE=reference&D=med7&NEWS=N&AN=19419621

972. Bussen D, Sailer M, Fuchs K-H, Thiede A. [Tumescent local anesthesia in proctologic surgery]. Tumeszenz-Lokalanasthesie bei proktologischen Eingriffen [Internet]. 2003;74(9):839–43. Available from: http://ovidsp.ovid.com/ovidweb.cgi?T=JS&PAGE=reference&D=med5&NEWS=N&AN=14504797

973. Kayaalp C, Olmez A, Aydin C, Piskin T. Tumescent local anesthesia for excision and flap procedures in treatment of pilonidal disease. Dis Colon Rectum [Internet]. 2009;52(10):1780–3. Available from: http://ovidsp.ovid.com/ovidweb.cgi?T=JS&PAGE=reference&D=med7&NEWS=N&AN=19966613

974. The effect of anesthetic techniques in pilonidal sinus surgery. Bahrain Med Bull [Internet]. 2004;26(3):102–4. Available from: http://ovidsp.ovid.com/ovidweb.cgi?T=JS&PAGE=reference&D=emed8&NEWS=N&AN=39208171

975. Orhon ZN, Koltka EN, Devrim S, Tüfekçi S, Doğru S, Çelik M. Epidural anesthesia for pilonidal sinus surgery: ropivacaine versus levobupivacaine. Korean J Anesthesiol [Internet]. 2015;68(2):141. Available from: http://ekja.org/Synapse/Data/PDFData/0011KJAE/kjae-68-141.pdf

976. Rastegarian A, Abedi HA, Sepidkar AA, Kheyrkhah N, Jahromi HK, Farzam M. The Use of Laryngeal Mask Airway in Pilonidal Cyst Excision after Muscle Relaxant (Atracurium) Injection in Prone Position. Biosci Biotechnol Res Asia [Internet]. 2014 Sep 30;11(2):875–8. Available from: http://www.biotech-asia.org/archive.php

977. I. I, T. M, S. D. Spinal versus general anesthesia in patients undergoing karydakis flap for pilonidal disease: Results of a prospective pilot study. Dis Colon Rectum [Internet]. 2013;56(4):e295--e296. Available from: http://ovidsp.ovid.com/ovidweb.cgi?T=JS&PAGE=reference&D=emed14&NEWS=N&AN=71045859

978. Almeida-Goncalves JC. A curative cryosurgical technique for advanced cancer of sacrococcygeal pilonidal sinuses. J Surg Oncol [Internet]. 2012;106(4):504–8. Available from: http://ovidsp.ovid.com/ovidweb.cgi?T=JS&PAGE=reference&D=med9&NEWS=N&AN=22488151

979. de Bree E, Zoetmulder FA, Christodoulakis M, Aleman BM, Tsiftsis DD. Treatment of malignancy arising in pilonidal disease. Ann Surg Oncol [Internet]. 2001;8(1):60–4. Available from: http://ovidsp.ovid.com/ovidweb.cgi?T=JS&PAGE=reference&D=med4&NEWS=N&AN=11206226

980. Pilipshen SJ, Gray G, Goldsmith E, Dineen P. Carcinoma arising in pilonidal sinuses. Ann Surg [Internet]. 1981;193(4):506–12. Available from: http://ovidsp.ovid.com/ovidweb.cgi?T=JS&PAGE=reference&D=med2&NEWS=N&AN=7212813

981. Alecha Gil J, Echenique-Elizondo M, Antonio Amondarain J, Górriz Arias G. Carcinoma epidermoide sobre seno pilonidal. Cirugía Española [Internet]. 2006 Jul;80(1):56. Available from: http://ovidsp.ovid.com/ovidweb.cgi?T=JS&PAGE=reference&D=emed9&NEWS=N&AN=44685806

982. A. M, E. D, A. A. Socioeconomical cost of acute pilonidal sinus surgery in district general hospital in UK. Int J Surg [Internet]. 2017;47(Supplement 1):S42. Available from: http://ovidsp.ovid.com/ovidweb.cgi?T=JS&PAGE=reference&D=emed18&NEWS=N&AN=619899726
